# Multi-Gram Synthesis of Enantiopure 1,5-Disubstituted Tetrazoles Via Ugi-Azide 3-Component Reaction

**DOI:** 10.3390/molecules23112758

**Published:** 2018-10-25

**Authors:** Pietro Capurro, Lisa Moni, Andrea Galatini, Christian Mang, Andrea Basso

**Affiliations:** 1 Dipartimento di Chimica e Chimica Industriale, Università degli Studi di Genova, Via Dodecaneso 31, 16146 Genoa, Italy; pietro.capurro@outlook.it (P.C.); lisa.moni@unige.it (L.M.); andrea@chimica.unige.it (A.G.); 2 AnalytiCon Discovery GmbH, Hermannswerder Haus 17, 14473 Potsdam, Germany; c.mang@ac-discovery.com

**Keywords:** multicomponent reactions, Ugi-azide, chiral imines, tetrazoles, diversity-oriented synthesis

## Abstract

Tetrazoles have been widely studied for their biological properties. An efficient route for large-scale synthesis of 1,5-disubstituted tetrazoles (1,5-DTs) is presented. The strategy exploits a reductive approach to synthetize a cyclic chiral imine substrate which is then converted into the target product through an Ugi-azide three-component reaction (UA-3CR). The final products are equipped with additional functionalities which can be further elaborated for the generation of combinatorial libraries of enantiopure heterocycles.

## 1. Introduction

**Figure 1:**
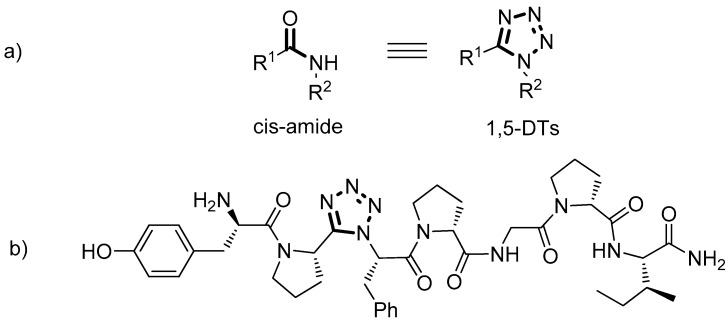
(**a**) 1,5-DTs as isosteres of *cis*-amide bonds. (**b**) Example of an opiate-like peptidomimetic containing the 1,5-DT structural moiety.

Since their discovery in 1885, tetrazoles have been widely studied, and their chemistry and biological properties have been deeply explored. In particular, syntheses of 1,5-disubstituted tetrazoles (1,5-DTs) have been thoroughly investigated as these compounds, being bioisosteres of *cis*-amide bond in peptides, play a relevant role in modern bioresearch (Figure 1a) [1]. 1,5-DTs have shown biological activity as HIV-protease inhibitors (e.g., analogues of Saquinavir are currently being studied), but they have also been proved to be good candidates for new antiseptic, anti-inflammatory, and analgesic drugs. Highly active nucleoside analogues and peptidomimetics (Figure 1b) containing the tetrazole ring have also been developed [2]. However, drug research is nowadays facing a slowdown, and the approval of new active compounds is stalling because of the low availability of adequate starting points for library generation. In fact, classical synthetic methods hardly permit differently functionalized products featuring a common scaffold to be obtained, and therefore a method to straightforwardly access complex structures with different decorations is highly desirable. Within this field, multicomponent reactions (MCRs) are a powerful tool that can be exploited to synthetize a plethora of potentially active structures with few steps and relatively simple building blocks [3,4,5]. A convenient synthesis of 1,5-DTs for combinatorial libraries and diversity-oriented synthesis (DOS) employs the Ugi-azide three- or four-component reaction (UA-3CR/UA-4CR), a modification of the classical and well-known Ugi reaction. Despite these considerations, very few examples of UA-3CRs on cyclic or chiral imines are reported [6,7] On the contrary, the Ugi-Jullié reaction with such substrates has been more widely exploited by us and others [8,9,10]. In order to fill this gap, we wish to report a novel synthetic route able to afford large amounts of enantiopure 1,5-DTs through a simple two-step protocol under very mild conditions starting from a chiral lactam formally derived from glutamic acid. These products can then be further processed for the generation of combinatorial libraries in accordance to synthetic approaches already applied by us to other substrates [11].

## 2. Results and Discussion

**Scheme 1:**
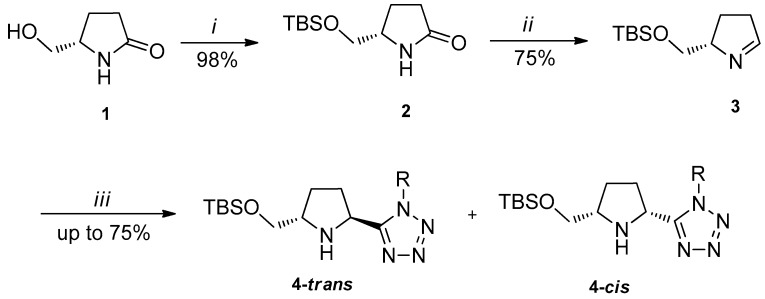
Synthetic route. Conditions: (i) TBS-Cl, imidazole, DCM, N_2_ atm., r.t., overnight; (ii) Cp_2_ZrHCl, THF, −20 °C to r.t, 1 h 30 min; (iii) R-NC, TMS-N_3_, MeOH, 0 °C, 30 min.

We have recently reported that cyclic chiral imines, which are easily synthetized via reduction of the corresponding lactam with Schwartz’s reagent, can be employed in the Betti reaction with phenol derivatives [12]. In order to expand this approach, we reasoned that the same imines could be ideal substrates for the UA-3CR. Starting from commercially available chiral lactam **1**, chiral imine **3** was synthetized in two steps via protection of the primary alcoholic group and subsequent Zr-mediated reduction of lactam **2** (Scheme 1). Protection of the hydroxyl functionality was essential for the reduction to occur smoothly, and the silyl protecting group was preferred to other derivatives (i.e., acetyl) that could interact with Schwartz’s reagent. Despite the fact that this reducing agent is widely used in organic synthesis, it is often used in small-scale reactions [13,14,15,16], and thus we developed a robust methodology that allowed us to react up to 6.0 g of substrate in a one pot process with a simple purification step. This straightforward procedure to prepare chiral imine **3** exceeds the one previously reported by us [8].

With imine **3** in hand, we moved to test the UA-3CR with different isocyanides. We found that the reaction of **3** with TMS-N_3_ and isocyanides occurred fast (30 min) under mild reaction conditions (MeOH, 0 °C). With this approach, the necessity of high reaction temperatures or prolonged times to force the imine formation (as reported by Dömling and co-workers) was avoided [17].

**Scheme 2:**
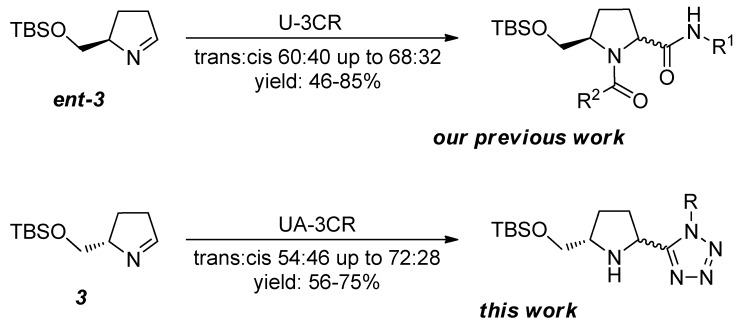
Analogies between yields and asymmetric induction for products of Ugi-Joullié and Ugi-azide reactions using cyclic chiral imine **3** and its enantiomer ***ent-3***.

Under these conditions, the diastereoselectivity of the process proved to be moderate, ranging from a 54:46 ratio up to 72:28, and usually resulting in around 68:32 (*trans*:*cis*). These results are consistent with previous studies by our group where compound ***ent*-3** (enantiomer of chiral imine **3**), when employed in Ugi-Joullié three-component reactions, gave similar results in terms of overall yield and asymmetric induction (Scheme 2) [8]. According to the commonly accepted mechanisms of these reactions, the stereoselection in both cases was determined by the facial selectivity of the incoming isocyanide and only marginally influenced by the substituent on the pyrroline ring.

However, as the two diastereoisomers were separable by column chromatography, the moderate induction did not represent a problem. In fact, both isomers are valid entry points for generating libraries for diversity-oriented synthesis applications.

**Scheme 3:**
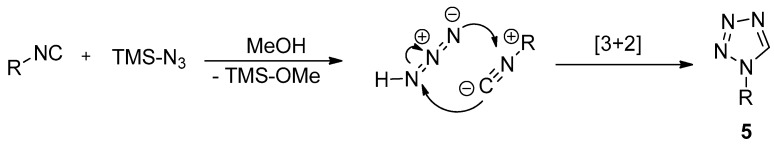
Mechanism of formation of 1-substituted tetrazole **5** via cycloaddition between the HN_3_ formed in situ and the isocyanide.

This synthetic protocol allowed us to synthetize multi-gram quantities (up to 5.0 g) of the targeted 1,5-DTs in just two steps from lactam **2**. Imine formation was fast (1 h 30 min) and its purification by filtration was trivial while the UA-3CR was even faster (30 min) and its products easily separated upon column chromatography. It is worth noting that in some cases (depending on the nature of the isocyanide employed), small quantities of 1-substituted tetrazole **5** was formed (Scheme 3). This product was derived from the cycloaddition reaction between the HN_3_ formed in situ and the isocyanide. However, with stoichiometric amounts of the azide source at low temperatures and in absence of an acid catalyst, the reaction is known to be rather slow, and thus the formation of the side product can be easily kept under 5 mol% [1]. The results of our scope with commercial isocyanides are reported in Table 1. During the scope, the synthetic route proved to be robust and very reliable, apart from isocyanides bearing acidic protons on the α position which reacted poorly (entries *l* and *m*). On the other hand, isocyanides bearing a basic nitrogen, although affording the desired products in comparable yield, reacted more slowly (entry *k*).

**Figure 2:**
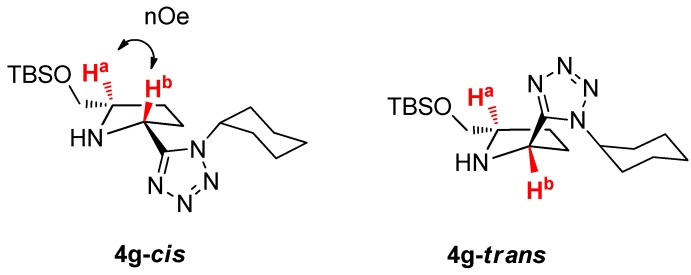
Irradiated protons on the *cis* and *trans* isomer of molecule **4g**. Protons H^a^ and H^b^ gave a smooth nOe only in the first case, allowing us to attribute the correct configuration to each of the two molecules.

The configuration of each diastereoisomer was determined via NMR NOESY-1D and 2D experiments. In particular, products with *cis* configuration showed a marked nOe effect between the protons directly bonded to the chirality centers, while products in *trans* configuration did not. The nOe effect was accurately measured on both isomers of product **4g** with NOESY-1D experiments (Figure 2). Upon irradiation of proton ***a*** in the *cis* isomer (molecule **4g-*cis***), proton ***b*** gave a smooth and clear nOe (2%). In the same molecule, irradiation of proton ***b*** induced a 1.5% nOe on proton ***a***. On the other hand, the same experiment on molecule **4g-*trans*** showed no visible nOe between the same irradiated protons. We attributed the configurations of the two diastereoisomers of molecule **4g** accordingly to these results. In all other couples of diastereoisomers, NOESY-2D experiments were performed. These experiments showed clear nOe effects between the respective H^a^ and H^b^ protons on the less abundant diastereoisomer, while the most abundant one showed no nOe at all between the very same protons. These results proved that, with each isocyanide employed, stereoselectivity was always in favor of the *trans* isomer.

## 3. Materials and Methods

### 3.1. General

Mono- and bidimensional NMR spectra (^1^H, ^13^C, gCOSY, gHSQC, gHMBC, 1D and 2D-NOESY) were obtained on a Varian (Palo Alto, CA, USA) Mercury 300 (300 MHz for ^1^H, 75 MHz for ^13^C) at 27 °C in CDCl_3_ + 0.03% TMS. Chemical shift values are reported in ppm (parts per million) using TMS signal (0.00 ppm) as reference. 

HPLC-UV were performed on a Varian (Palo Alto, CA, USA) Agilent 1100 series using a Phenomenex (Torrance, CA, USA) Gemini C_6_-Phenyl 150 × 3 mm column, VWD = 210 nm. Sample concentration 1 mg/mL (MeOH + 0.1% HCOOH:H_2_O + 0.1% HCOOH 1:1), injection volume 5 µL, flow 0.34 mL/min, T = 30 °C, gradient 0 min = 90% A, 20 min = 0% A (A: H_2_O + 0.1% HCOOH, B: MeOH + 0.1% HCOOH).

ESI-MS analyses were performed on a Microsaic (Woking, Surrey, UK) 4000 MiD (full scan 100-800 *m*/*z*, positive ions, tip voltage 750 V).

HR-MS analyses were carried out on a Waters (Milford, MA, USA) Synapt G2 QToF mass spectrometer. MS signals were acquired from 50 to 1200 *m*/*z* in ESI positive ionization mode.

TLC analyses were performed on Merck (Darmstadt, Germany) 60 F_254_ 0,25 mm silica plates and visualized with ninidrine (900 mg ninidrine, 300 mL *n*-BuOH, 9 mL AcOH) or Hanessian stain (21 g (NH_4_)MoO_4_∙4H_2_O, 1 g Ce(SO_4_)_2_, 31 mL H_2_SO_4_ 98%, 369 mL H_2_O) development (dipping in the corresponding solution and warming). 

Flash chromatographies were performed using SiO_2_ 230-400 mesh (Merck Ge Duran SI60) and solvent bought from Sigma-Aldrich, Tokyo Chemical Industry, Carlo Erba, Honeywell/Riedel-de-Haen, Alfa Aesar, VWR International, Acros Organics, Merck (Darmstadt, Germany). Celite employed for filtration was Acros Organic Celite 545 (Thermo Fischer Scientific, Geel, Belgium).

### 3.2. Chemistry

#### 3.2.1. Protection of Chiral Lactam **1**

(S)-HMP (**1**) (6.00 g, 52.1 mmol, 1 eq.), TBS-Cl (9.42 g, 62.5 mmol, 1.2 eq.) and imidazole (7.08 g, 104 mmol, 2 eq.) were added to a flask under nitrogen atmosphere, dissolved in 120 mL of dry DCM (0.4 M), and left reacting at room temperature overnight. The crude was diluted to 150 mL and was washed three times with deionized water and once with brine. Eventually, the crude was purified by flash chromatography (PE:EtOAc 1:9, EtOAc:MeOH 9:1). Yield: 11.65 g (97%).

#### 3.2.2. Reduction with Schwartz’s Reagent of Chiral Lactam **2** to Imine **3**

A solution of TBS-(S)-HMP (**2**) (5.35 g, 23.3 mmol, 1 eq.) in dry THF (50 mL, 0.5 M) chilled at 0 °C was gently dripped under vigorous stirring and an argon atmosphere into a suspension of Cp_2_ZrHCl (i.e., Schwartz’s reagent, 12.6 g, 48.9 mmol, 2.1 eq.) in dry THF (100 mL, 0.5 M) at −20 °C. After 1 h 30 min, the solvent was removed under reduced pressure and the crude was suspended in iced pentane. The precipitated Cp_2_Zr=O waste was filtered off under vacuum over celite. The eluted solution was dried at the rotary evaporator. Yield: 3.73 g (75%).

#### 3.2.3. UA-3CR of Imine **3** with TMS-N_3_ and R-NC

Imine **3** (4.50 g, 21.1 mmol, 1 eq.) was dissolved in dry MeOH (80 mL, 0.25 M) under nitrogen atmosphere. The solution was chilled at 0 °C in ice bath, then R-NC (23.2 mmol, 1.1 eq.) and TMS-N_3_ (3.06 mL, 2.67 g, 1.1 eq.) were added in sequence. After 30 min, the solution was checked for complete consumption of the imine. The solvent was removed under reduced pressure and the crude was eventually purified by flash chromatography. See Supplementary Materials for characterization of the compounds.

## 4. Conclusions

In conclusion, the synthetic route that we optimized and reported allows easy access to bulk amounts of diverse 1,5-DTs of general formula **4** as mixtures of diastereoisomers. Such mixtures can be easily separated upon column chromatography and the resulting enantiopure diastereoisomers can both be used as valid entry points for the creation of combinatorial libraries. This synthetic path is particularly advantageous for its mild conditions and operational simplicity as well as its robustness and reliability. Moreover, the synthetic pathway we propose proved to be strongly time efficient, as the whole procedure from protection of the substrate to the purification of the final product could be performed in less than two days, yielding usually more than 4 g of final products. Similar libraries have been further diversified by our exploitation of the additional functionalities on the pyrrolidine ring and generating large collections of enantiopure heterocycles to be employed in drug discovery platforms.

## Figures and Tables

**Table 1:** Results for UA-3CR depicted in Scheme 1 under condition *iii*.

Entry	R ^1^	Yield [%] ^2^	d.r. (*trans*:*cis*) ^3^
a	Me	62	62:38
b	cPr	75	64:36
c	cBu	56	62:38
d	tBu	74	54:46
e	nBu	74	67:33
f	iBu	59	69:31
g	cHex	65	67:33
h	Bn	73	62:38
i	(2,6-diMe)Ph	76	62:38
j	(4-MeO)Ph	46	69:31
k ^4^	2-MorphEt	66	72:28
l	EtOOC-CH_2_-	*no reaction*	
m	Tos-CH_2_-	*no reaction*	

^1^ R group of products **4-*trans*** and **4-*cis***; ^2^ isolated yield; ^3^ d.r. on crude products, determined via ^1^H-NMR; ^4^ reaction time 48 h.

## Supplementary Materials

Supplementary Material associated with this article can be found, in the online version. Analytical data for tetrazoles **4a**–**k** and copies of NMR spectra.
