# Supplementary material for: Multi-Gram Synthesis of Enantiopure 1,5-Disubstituted Tetrazoles Via Ugi-Azide 3-Component Reaction
Source: Molecules. 2018 Oct 25;23(11):2758. doi: 10.3390/molecules23112758 (PMC6278549; doi:10.3390/molecules23112758)

Detailed Spectroscopic Data

for the manuscript

Multi-gram synthesis of enantiopure 1,5-disubstituted tetrazoles via Ugi-azide 3-component reaction

Pietro Capurro, Lisa Moni, Andrea Galatini, Christian Mang, Andrea Basso*

List of contents

Analytical data for compounds 4a-k **S2**
Copies of NMR Spectra **S7**

5-((2RS,5S)-5-(((*tert*-butyldimethylsilyl)oxy)methyl)pyrrolidin-2-yl)-1-methyl-1H-tetrazole (4a)

$C_{13}H_{27}N_5OSi$, MW 297.48, yield 62%, d.r. 62:38 (*trans*:*cis*), chromatography PE:EtOAc 7:3 → EtOAc:MeOH 8:2. **trans isomer (2R)**: dark yellow oil; R_f = 0.39 [PE:EtOAc 3:2, ninidrine]; ^{1}H-NMR ($CDCl_3$): δ = 4.67 (t, *J* = 6.4 Hz, 1H), 4.13 (s, 3H), 3.66 (ddd, *J* = 10.2, 4.1, 1.0 Hz, 1H), 3.57 (ddd, *J* = 10.2, 5.2, 1.0 Hz, 1H), 3.46 – 3.32 (m, 1H), 2.36 – 1.97 (m, 4H), 1.78 – 1.63 (m, 1H), 0.91 (s, 9H), 0.08 (s, 6H); ^{13}C-NMR ($CDCl_3$): δ = 157.08, 65.01, 59.93, 52.19, 34.34, 31.34, 27.74, 26.05, 18.45, -5.22; t_r = 11.09 min; ESI-MS [M+H] 298.4; diastereoisomeric purity (HPLC) > 99.9%; HRMS (ESI+): calculated for $C_{13}H_{28}N_5OSi^+$: 298.2058, found 298.2051 **cis isomer (2S)**: brown oil, R_f = 0.24 [PE:EtOAc 3:2, ninidrine], ^{1}H-NMR ($CDCl_3$): δ = 4.62 (t, *J* = 7.6 Hz, 1H), 4.17 (s, 3H), 3.66 (dd, *J* = 10.0, 4.7 Hz, 1H), 3.55 (dd, *J* = 10.0, 5.9 Hz, 1H), 3.47 – 3.33 (m, 1H), 2.37 – 1.89 (m, 4H), 1.80 – 1.67 (m, 1H), 0.88 (s, 9H), 0.05 (s, 6H); ^{13}C-NMR ($CDCl_3$): δ = 156.49, 66.56, 60.89, 52.64, 34.61, 30.80, 27.51, 26.02, 18.45, -5.22, t_r = 12.43 min, ESI-MS [M+H] 298.0, diastereoisomeric purity 96.9%; HRMS (ESI+): calculated for $C_{13}H_{28}N_5OSi^+$: 298.2058, found 298.2063

5-((2RS,5S)-5-(((*tert*-butyldimethylsilyl)oxy)methyl)pyrrolidin-2-yl)-1-cyclopropyl-1H-tetrazole (4b)

$C_{15}H_{29}N_5OSi$, MW 323.52, yield 75%, d.r. 64:36 (*trans*:*cis*), chromatography PE:EtOAc 7:3 → EtOAc:MeOH 8:2. **trans isomer (2R)**: yellow foam; R_f = 0.33 [PE:EtOAc 3:2, ninidrine]; ^{1}H-NMR ($CDCl_3$): δ 4.67 (dd, *J* = 7.4, 6.1 Hz, 1H), 3.78 – 3.64 (m, 1H), 3.61 (dd, *J* = 10.1, 4.5 Hz, 1H), 3.53 (dd, *J* = 10.1, 5.3 Hz, 1H), 3.43 (tt, *J* = 6.8, 4.9 Hz, 1H), 2.48 (broad s, 1H), 2.34 – 1.97 (m, 3H), 1.67 (dddd, *J* = 12.5, 8.9, 7.4, 6.6 Hz, 1H), 1.39 – 1.10 (m, 4H), 0.86 (s, 9H), 0.03 (s, 6H); ^{13}C-NMR ($CDCl_3$): δ = 158.64, 65.35, 59.66, 51.87, 30.93, 28.67, 27.61, 25.87, 18.25, 7.02, -5.39; t_r = 12.86 min; ESI-MS [M+H] 324.3; diastereoisomeric purity (HPLC) > 99.9%; HRMS (ESI+): calculated for $C_{15}H_{30}N_5OSi^+$: 324.2214, found 324.2209 **cis isomer (2S)**: yellow oil; R_f = 0.16 [PE:EtOAc 3:2, ninidrine]; ^{1}H-NMR ($CDCl_3$): δ = 4.59 (t, *J* = 7.5 Hz, 1H), 3.84 (tdd, *J* = 7.3, 4.1, 3.6 Hz, 1H), 3.64 (dd, *J* = 10.0, 4.9 Hz, 1H), 3.54 (dd, *J* = 10.0, 5.8 Hz, 1H), 3.42 – 3.31 (m, 1H), 2.35 (broad s, 1H), 2.32 – 2.06 (m, 2H), 1.94 (dddd, *J* = 12.4, 8.1, 7.3, 6.1 Hz, 1H), 1.76 (ddt, *J* = 12.6, 9.0, 7.2 Hz, 1H), 1.44 – 1.12 (m, 4H), 0.85 (s, 9H), 0.01 (s, 6H); ^{13}C-NMR ($CDCl_3$): δ = 157.97, 66.03, 60.87, 52.37, 30.56, 28.95, 27.59, 25.86, 18.27, 7.32, 7.01, -5.39; t_r = 13.37 min; ESI-MS [M+H] 324.3; diastereoisomeric purity (HPLC) > 99.9%; HRMS (ESI+): calculated for $C_{15}H_{30}N_5OSi^+$: 324.2214, found 324.2201

5-((2RS,5S)-5-(((*tert*-butyldimethylsilyl)oxy)methyl)pyrrolidin-2-yl)-1-cyclobutyl-1H-tetrazole (4c)

$C_{16}H_{31}N_5OSi$, MW 337.54, yield 56%, d.r. 62:38 (*trans*:*cis*), chromatography PE:EtOAc 7:3 → 3:7. **trans isomer (2R)**: white solid, m.p. 78.7 - 80.0 °C; R_f = 0.63 [PE:EtOAc 3:2, ninidrine]; ^{1}H-NMR ($CDCl_3$): δ = 5.21 – 5.06 (m, 1H), 4.57 (dd, *J* = 7.2, 6.2 Hz, 1H), 3.64 (dd, *J* = 10.1, 4.5 Hz, 1H), 3.56 (dd, *J* = 10.1, 5.4 Hz, 1H), 3.44 (p, *J* = 6.6 Hz, 1H), 2.86 – 2.68 (m, 2H), 2.62 – 2.45 (m, 2H), 2.42 – 2.21 (m, 2H), 2.18 – 1.87 (m, 4H), 1.78 – 1.60 (m, 1H), 0.90 (s, 9H), 0.08 (s, 6H); ^{13}C-NMR ($CDCl_3$): δ = 156.31, 65.42, 59.75, 52.09, 51.55, 31.57, 30.28, 30.26, 27.60, 25.99, 18.37, 15.16, -5.27; t_r = 9.39 min; ESI-MS [M+H] 338.3; diastereoisomeric purity (HPLC) > 99.9%; HRMS (ESI+): calculated for $C_{16}H_{32}N_5OSi^+$: 338.2371, found 338.2390 **cis isomer (2S)**: yellowish solid, m.p. 56.9 – 58.1 °C; R_f = 0.37 [PE:EtOAc 3:2, ninidrine]; ^{1}H-NMR ($CDCl_3$): δ = 5.25 (p, *J* = 8.4 Hz, 1H), 4.50 (t, *J* = 7.5 Hz, 1H), 3.67 (dd, *J* = 10.0, 4.7 Hz, 1H), 3.57 (dd, *J* = 10.0, 5.9 Hz, 1H), 3.43 – 3.31 (m, 1H), 2.87 – 2.69 (m, 2H), 2.53 (ddt, *J* = 12.3, 8.0, 4.0 Hz, 2H), 2.30 – 1.85 (m, 5H), 1.80 – 1.66 (m, 2H), 0.89 (s, 9H), 0.05 (s, 6H); ^{13}C-NMR ($CDCl_3$): δ = 155.71, 77.16, 66.25, 60.97, 52.70, 51.67, 31.19,

30.44, 30.30, 27.52, 26.01, 18.44, 15.16, -5.24; t_r = 10.02 min; ESI-MS [M+H] 338.3; diastereoisomeric purity (HPLC) 97.5 %; HRMS (ESI+): calculated for $C_{16}H_{32}N_5OSi^+$: 338.2371, found 324.2384.

1-(*tert*-butyl)-5-((2RS,5S)-5-(((*tert*-butyldimethylsilyl)oxy)methyl)pyrrolidin-2-yl)-1H-tetrazole (4d)
$C_{16}H_{33}N_5OSi$, MW 339.56, yield 74%, d.r. 54:46 (*trans*:*cis*), chromatography PE:EtOAc 7:3 → EtOAc:MeOH 8:2. **trans isomer (2R)**: yellow oil; R_f = 0.58 [PE:EtOAc 3:2, ninidrine]; ^{1}H-NMR ($CDCl_3$): *δ* = 4.78 – 4.67 (m, 1H), 3.69 – 3.53 (m, 3H), 2.37 – 2.09 (m, 4H), 1.76 (s, 9H), 1.75 – 1.67 (m, 1H), 0.90 (s, 9H), 0.07 (s, 6H); ^{13}C-NMR ($CDCl_3$): *δ* = 157.67, 66.49, 61.17, 59.93, 53.64, 33.16, 30.24, 27.99, 26.06, 18.45, -5.19; t_r = 13.79 min; ESI-MS [M+H] 340.3; diastereoisomeric purity (HPLC) > 99.9%; HRMS (ESI+): calculated for $C_{16}H_{34}N_5OSi^+$: 340.2527, found 340.2539 **cis isomer (2S)**: dark yellow oil; R_f = 0.22 [PE:EtOAc 3:2, ninidrine]; ^{1}H-NMR ($CDCl_3$): *δ* = 4.54 (t, *J* = 7.3 Hz, 1H), 3.76 (dd, *J* = 10.1, 4.7 Hz, 1H), 3.69 (dd, *J* = 10.1, 4.9 Hz, 1H), 3.31 (tt, *J* = 7.4, 4.8 Hz, 1H), 2.28 – 2.14 (m, 2H), 2.02 – 1.88 (m, 3H), 1.76 (s, 9H), 0.89 (s, 9H), 0.06 (s, 6H); ^{13}C-NMR ($CDCl_3$): *δ* = 56.87, 64.89, 61.60, 61.21, 54.24, 33.27, 30.24, 28.45, 26.04, 18.44, -5.25; t_r = 14.40 min; ESI-MS [M+H] 340.4; diastereoisomeric purity (HPLC) 96.6%; HRMS (ESI+): calculated for $C_{16}H_{34}N_5OSi^+$: 340.2527, found 340.2520

1-(*n*-butyl)-5-((2RS,5S)-5-(((*tert*-butyldimethylsilyl)oxy)methyl) pyrrolidin-2-yl)-1H-tetrazole (4e)
$C_{16}H_{33}N_5OSi$**,** MW 339.56, yield 74%, d.r. 67:33 (*trans*:*cis*), DCM:Et_2O 10:0 → 5:5. **trans isomer (2R)**: pale yellow oil; R_f = 0.55 [PE:EtOAc 3:2, ninidrine]; ^{1}H-NMR ($CDCl_3$): *δ* = 4.66 – 4.55 (m, 1H), 4.43 (td, *J* = 7.3, 1.9 Hz, 2H), 3.66 (dd, *J* = 10.2, 4.3 Hz, 1H), 3.57 (dd, *J* = 10.1, 5.3 Hz, 1H), 3.46 – 3.37 (m, 1H), 2.46 (s, 1H), 2.35 – 2.14 (m, 2H), 2.13 – 1.98 (m, 1H), 1.91 (p, *J* = 7.5 Hz, 2H), 1.71 (dddd, *J* = 12.6, 9.0, 7.5, 6.7 Hz, 1H), 1.39 (h, *J* = 7.3 Hz, 2H), 0.97 (td, *J* = 7.0, 0.8 Hz, 3H), 0.91 (s, 9H), 0.07 (s, 6H); ^{13}C-NMR ($CDCl_3$): *δ* = 156.90 , 65.29 , 59.89 , 52.24 , 47.46 , 31.87 , 31.80 , 27.74 , 26.01 , 19.86 , 18.41 , 13.60 , -5.27; t_r = 14.56 min; ESI-MS [M+H] 340.4; diastereoisomeric purity (HPLC) > 99.9%; HRMS (ESI+): calculated for $C_{16}H_{34}N_5OSi^+$: 340.2527, found 340.2512 **cis isomer (2S)**: pale yellow oil; R_f = 0.32 [PE:EtOAc 3:2, ninidrine]; ^{1}H-NMR ($CDCl_3$): *δ* = 4.62 – 4.33 (m, 3H), 3.66 (dd, *J* = 10.0, 4.8 Hz, 1H), 3.57 (dd, *J* = 10.0, 5.9 Hz, 1H), 3.38 (tdd, *J* = 7.5, 5.9, 4.8 Hz, 1H), 2.40 – 2.05 (m, 3H), 2.04 – 1.84 (m, 3H), 1.75 (ddt, *J* = 12.6, 9.4, 7.0 Hz, 1H), 1.39 (h, *J* = 7.4 Hz, 2H), 0.97 (t, *J* = 7.4 Hz, 3H), 0.88 (s, 9H), 0.05 (s, 6H); ^{13}C-NMR ($CDCl_3$): *δ* = 156.30, 66.26, 61.07, 52.71, 47.60, 31.99, 31.38 , 27.65, 26.02, 19.91, 18.43, 13.66, -5.23; t_r = 15.23 min; ESI-MS [M+H] 340.2; diastereoisomeric purity (HPLC) 97.6%; HRMS (ESI+): calculated for $C_{16}H_{34}N_5OSi^+$: 340.2527, found 340.2519

5-((2RS,5S)-5-(((tert-butyldimethylsilyl)oxy)methyl)pyrrolidin-2-yl)-1-isobutyl-1H-tetrazole (4f)

$C_{16}H_{33}N_5OSi$, MW 339.56, yield 59%, d.r. 69:31 (*trans*:*cis*), chromatography PE:EtOAc 8:2 → 5:5. **trans isomer (2R)**: yellow oil; R_f = 0.54 [PE:EtOAc 3:2, ninidrine]; ^{1}H-NMR ($CDCl_3$): *δ* = 4.58 (dd, *J* = 7.2, 6.3 Hz, 1H), 4.35 – 4.13 (m, 2H), 3.66 (dd, *J* = 10.2, 4.3 Hz, 1H), 3.57 (dd, *J* = 10.2, 5.2 Hz, 1H), 3.42 (tt, *J* = 7.0, 4.8 Hz, 1H), 2.60 – 2.16 (m, 4H), 2.16 – 1.97 (m, 1H), 1.71 (dddd, *J* = 12.5, 8.9, 7.5, 6.7 Hz, 1H), 0.98 (d, *J* = 2.0 Hz, 3H), 0.96 (d, *J* = 2.0 Hz, 3H), 0.91 (s, 9H), 0.07 (s, 6H); ^{13}C-NMR ($CDCl_3$): *δ* = 157.14, 65.26, 59.74, 54.39, 52.11, 31.79, 29.12, 27.62, 25.88, 19.84, 18.26, -5.38; t_r = 9.89 min; ESI-MS [M+H] 340.5; diastereoisomeric purity (HPLC) > 99.9%%; HRMS (ESI+): calculated for $C_{16}H_{34}N_5OSi^+$: 340.2527, found 340.2520 **cis isomer (2S)**: yellow oil; R_f = 0.41 [PE:EtOAc 3:2, ninidrine]; ^{1}H-NMR ($CDCl_3$): *δ* = 4.46 (t, *J* =

7.5 Hz, 1H), 4.28 (dd, *J* = 13.0, 7.7 Hz, 1H), 4.19 (dd, *J* = 13.9, 7.7 Hz, 1H), 3.63 (ddd, *J* = 10.0, 4.9, 0.6 Hz, 1H), 3.54 (ddd, *J* = 10.0, 5.8, 0.6 Hz, 1H), 3.34 (ddd, *J* = 12.7, 7.4, 5.3 Hz, 1H), 2.61 – 2.03 (m, 4H), 2.00 – 1.85 (m, 1H), 1.80 – 1.65 (m, 1H), 0.97 – 0.89 (m, 6H), 0.84 (s, 9H), 0.03 –0.02 (m, 6H); ^{13}C-NMR ($CDCl_3$): δ = 156.56, 65.95, 60.96, 54.44, 52.54, 31.40, 29.12, 27.59, 25.84, 19.86, 19.80, 18.25, -5.41; t_r = 10.40 min; ESI-MS [M+H] 340.6; diastereoisomeric purity (HPLC) > 99.9%; HRMS (ESI+): calculated for $C_{16}H_{34}N_5OSi^+$: 340.2527, found 340.2537

5-((2RS,5S)-5-(((*tert*-butyldimethylsilyl)oxy)methyl)pyrrolidin-2-yl)-1-cyclohexyl-1H-tetrazole (4g)

$C_{18}H_{35}N_5OSi$, MW 365.60, yield 65%, d.r. 67:33 (*trans*:*cis*), PE:EtOAc 7:3 → 2:8. **trans isomer (2R)**: white solid, m.p. 122.8 – 123.7 °C; R_f = 0.62 [PE:EtOAc 3:2, ninidrine]; ^{1}H-NMR ($CDCl_3$): δ = 4.59 (dd, *J* = 7.3, 6.2 Hz, 1H), 4.54 – 4.40 (m, 1H), 3.66 (dd, *J* = 10.1, 4.4 Hz, 1H), 3.57 (dd, *J* = 10.1, 5.3 Hz, 1H), 3.51 – 3.37 (m, 1H), 2.35 – 1.89 (m, 10H), 1.83 – 1.63 (m, 2H), 1.50 – 1.22 (m, 3H), 0.91 (s, 9H), 0.08 (s, 6H); ^{13}C-NMR ($CDCl_3$): δ = 156.26, 65.60, 59.91, 58.02, 52.26, 33.17, 32.97, 31.92, 27.81, 26.05, 25.54, 25.02, 18.43, -5.23; t_r = 15.32 min; ESI-MS [M+H] 366.4; diastereoisomeric purity (HPLC) > 99.9%; HRMS (ESI+): calculated for $C_{18}H_{36}N_5OSi^+$: 366.2684, found 366.2663 **cis isomer (2S)**: white solid, m.p. 92.3 – 93.9 °C; R_f = 0.45 [PE:EtOAc 3:2, ninidrine]; ^{1}H-NMR ($CDCl_3$): δ = 4.68 – 4.46 (m, 2H), 3.67 (dd, *J* = 9.9, 4.9 Hz, 1H), 3.56 (dd, *J* = 9.9, 6.1 Hz, 1H), 3.46 – 3.30 (m, 1H), 2.33 – 1.85 (m, 10H), 1.82 – 1.67 (m, 2H), 1.50 – 1.29 (m, 3H), 0.88 (s, 9H), 0.05 (s, 6H); ^{13}C-NMR ($CDCl_3$): δ = 155.64, 66.37, 61.12, 58.09, 52.66, 33.23, 33.00, 31.29, 27.78, 26.03, 25.63, 25.59, 25.03, 18.44, -5.22; t_r = 16.00 min; ESI-MS [M+H] 366.4; diastereoisomeric purity (HPLC) 98.5%; HRMS (ESI+): calculated for $C_{18}H_{36}N_5OSi^+$: 366.2684, found 366.2670.

1-benzyl-5-((2RS,5S)-5-(((*tert*-butyldimethylsilyl)oxy)methyl) pyrrolidin-2-yl)-1H-tetrazole (4h)

$C_{19}H_{31}N_5OSi$, MW 373.58, yield 73%, d.r. 62:38 (*trans*:*cis*), chromatography PE:EtOAc 8:2 → 3:7. **trans isomer (2R)**: yellow oil; R_f = 0.58 [PE:EtOAc 3:2, ninidrine]; ^{1}H-NMR ($CDCl_3$): δ = 7.46 – 7.11 (m, 5H), 5.78 (d, *J* = 15.1 Hz, 1H), 5.71 (d, *J* = 15.1 Hz, 1H), 4.49 (dd, *J* = 7.2, 6.3 Hz, 1H), 3.62 (dd, *J* = 10.2, 4.2 Hz, 1H), 3.51 (dd, *J* = 10.2, 5.4 Hz, 1H), 3.33 (tdd, *J* = 6.9, 5.4, 4.2 Hz, 1H), 2.21 – 1.83 (m, 4H), 1.61 (dddd, *J* = 12.4, 8.6, 7.5, 6.6 Hz, 1H), 0.90 (s, 9H), 0.06 (s, 6H); ^{13}C-NMR ($CDCl_3$): δ = 157.04, 134.24, 129.18, 128.79, 127.71, 65.06, 59.86, 52.39, 51.28, 31.35, 27.55, 26.04, 18.42, -5.26; t_r = 14.12 min; ESI-MS [M+H] 374.2; diastereoisomeric purity (HPLC) > 99.9%; HRMS (ESI+): calculated for $C_{19}H_{32}N_5OSi^+$: 374.2371, found 374.2355 **cis isomer (2S)**: yellowish solid, m.p. 46.1 – 47.3 °C; R_f = 0.40 [PE:EtOAc 3:2, ninidrine]; ^{1}H-NMR ($CDCl_3$): δ = 7.41 – 7.15 (m, 5H), 5.79 (d, *J* = 0.8 Hz, 2H), 4.45 (t, *J* = 7.4 Hz, 1H), 3.62 (dd, *J* = 10.0, 4.8 Hz, 1H), 3.54 (dd, *J* = 10.0, 6.0 Hz, 1H), 3.34 (tdd, *J* = 7.4, 6.0, 4.8 Hz, 1H), 2.22 – 1.78 (m, 4H), 1.63 (ddt, *J* = 12.6, 9.2, 7.1 Hz, 1H), 0.88 (s, 9H), 0.04 (s, 6H); ^{13}C-NMR ($CDCl_3$): δ = 156.54, 134.38, 129.16, 128.75, 127.65, 66.51, 61.02, 52.85, 51.24, 31.14, 27.45, 26.05, 18.46, -5.20; t_r = 14.86 min; ESI-MS [M+H] 374.4; diastereoisomeric purity (HPLC) 96.7%; HRMS (ESI+): calculated for $C_{19}H_{32}N_5OSi^+$: 374.2371, found 374.2363.

5-((2RS,5S)-5-(((tert-butyldimethylsilyl)oxy)methyl)pyrrolidin-2-yl)-1-(2,6-dimethyphenyl)-1H-tetrazole (4i)

$C_{20}H_{33}N_5OSi$, MW 387.60, yield 76%, d.r. 62:38 (*trans*:*cis*), chromatography PE:Et_2O 7:3 → 3:7. **trans isomer (2R)**: yellow oil; R_f = 0.70 [PE:EtOAc 3:2, ninidrine]; ^{1}H-NMR ($CDCl_3$): δ = 7.37 – 7.29 (m, 1H), 7.17 (d, *J* = 7.3 Hz, 2H), 4.16 (t, *J* = 6.7 Hz, 1H), 3.56 – 3.37 (m, 3H), 2.21 (broad s, 1H), 2.14 – 1.97 (m, 3H), 1.94 (s, 3H), 1.89 (s, 3H), 1.61 – 1.48 (m, 1H), 0.79 (s, 9H), -0.05 (s, 6H); ^{13}C-NMR ($CDCl_3$): δ = 158.60, 135.98, 135.52, 132.06, 130.81, 128.85, 128.69, 65.59, 59.63, 51.42, 31.28, 27.56, 25.81, 18.16, 17.52, 17.38, -5.49*;* t_r = 15.09 min*;* ESI-MS [M+H] 388.4; diastereoisomeric purity (HPLC) > 99.9%; HRMS (ESI+): calculated for $C_{20}H_{34}N_5OSi^+$: 388.2527, found 388.2521 **cis isomer (2S)**: yellow solid, m.p. 67.0 – 68.2 °C; R_f = 0.58 [PE:EtOAc 3:2, ninidrine]; ^{1}H-NMR ($CDCl_3$): δ = 7.37 – 7.30 (m, 1H), 7.18 (d, *J* = 7.4 Hz, 2H), 4.13 (t, *J* = 7.4 Hz, 1H), 3.46 (qd, *J* = 9.9, 5.6 Hz, 2H), 3.28 – 3.13 (m, 1H), 2.30 – 1.98 (m, 3H), 1.96 (s, 3H), 1.91 (s, 3H), 1.87 – 1.61 (m, 2H), 0.84 (s, 9H), 0.00 (s, 6H); ^{13}C-NMR ($CDCl_3$): δ = 158.18, 136.21, 135.75, 132.32, 130.90, 128.91, 128.75, 66.09, 60.88, 51.89, 30.93, 27.90, 25.97, 18.33, 17.69, 17.56, -5.30*;* t_r = 16.92 min*;* ESI-MS [M+H] 388.4; diastereoisomeric purity (HPLC) 97.0%; HRMS (ESI+): calculated for $C_{20}H_{34}N_5OSi^+$: 388.2527, found 388.2538.

5-((2RS,5S)-5-(((tert-butyldimethylsilyl)oxy)methyl)pyrrolidin-2-yl)-1-(4-methoxyphenyl)-1H-tetrazole (4j)

$C_{19}H_{31}N_5O_2Si$, MW 389.58, yield 46%, d.r. 69:31 (*trans*:*cis*), chromatography PE:Et_2O 5:5 → Et_2O:EtOH 8:2. **trans isomer (2R)**: dark green-black oil; R_f = 0.52 [PE:EtOAc 3:2, ninidrine]; ^{1}H-NMR ($CDCl_3$): δ = 7.43 (d, *J* = 8.9 Hz, 2H), 7.04 (d, *J* = 8.9 Hz, 2H), 4.47 (t, *J* = 6.7 Hz, 1H), 3.88 (s, 3H), 3.62 – 3.46 (m, 3H), 2.21 – 2.00 (m, 3H), 1.83 (s, 1H), 1.71 – 1.56 (m, 1H), 0.85 (s, 9H), 0.02 (s, 6H); ^{13}C-NMR ($CDCl_3$): δ = 161.06, 158.06, 126.92, 126.74, 115.00, 65.71, 59.89, 55.82, 51.81, 31.81, 27.90, 26.03, 18.41, -5.23*;* t_r = 10.56 min*;* ESI-MS [M+H] 390.4; diastereoisomeric purity (HPLC) > 99.9%; HRMS (ESI+): calculated for $C_{19}H_{32}N_5O_2Si^+$: 390.2320, found 390.2309 **cis isomer (2S)**: dark red-black oil; R_f = 0.36 [PE:EtOAc 3:2, ninidrine]; ^{1}H-NMR ($CDCl_3$): δ = 7.45 (d, *J* = 9.0 Hz, 2H), 7.04 (d, *J* = 9.0 Hz, 2H), 4.36 (t, *J* = 7.4 Hz, 1H), 3.89 (s, 3H), 3.64 (dd, *J* = 10.0, 5.0 Hz, 1H), 3.55 (dd, *J* = 10.0, 5.7 Hz, 1H), 3.28 (tt, *J* = 7.3, 5.3 Hz, 1H), 2.26 – 2.03 (m, 2H), 1.94 – 1.67 (m, 3H), 0.88 (s, 9H), 0.05 (s, 6H); ^{13}C-NMR ($CDCl_3$): δ = 161.09, 157.40, 127.00, 126.80, 114.93, 65.86, 61.30, 55.81, 52.22, 31.37, 28.03, 26.05, 18.44, -5.21*;* t_r = 11.15 min*;* ESI-MS [M+H] 390.4; diastereoisomeric purity (HPLC) 98.0%; HRMS (ESI+): calculated for $C_{19}H_{32}N_5O_2Si^+$: 390.2320, found 390.2331.

4-(2-(5-((2RS,5S)-5-(((*tert*-butyldimethylsilyl)oxy)methyl)pyrrolidin-2-yl)-1H-tetrazol-1-yl)ethyl)morpholine (4k)

$C_{18}H_{36}N_6O_2Si$, MW 396.61, yield 66%, d.r. 72:28 (*trans*:*cis*), chromatography Et_2O:EtOH 40:1 then PE:DCM:EtOH 7:2:1 → 6:2:2. **trans isomer (2R)**: yellow-orange oil; R_f = 0.71 [Et_2O:EtOH 20:1, ninidrine]; ^{1}H-NMR ($CDCl_3$): δ = 4.70 – 4.49 (m, 3H), 3.70 – 3.61 (m, 5H), 3.56 (dd, *J* = 10.2, 5.3 Hz, 1H), 3.42 – 3.32 (m, 1H), 2.89 (t, *J* = 6.3 Hz, 2H), 2.53 – 2.46 (m, 4H), 2.36 – 2.17 (m, 3H), 2.13 – 2.00 (m, 1H), 1.78 – 1.62 (m, 1H), 0.90 (s, 9H), 0.07 (s, 6H); ^{13}C-NMR ($CDCl_3$): δ = 157.28, 66.92, 65.28, 59.82, 57.91, 53.85, 52.37, 45.16, 31.57, 27.64, 26.04, 18.45, -5.24*;* t_r = 5.43 min*;* ESI-MS [M+H] 397.4; diastereoisomeric purity (HPLC) > 99.9%; HRMS (ESI+): calculated for $C_{18}H_{37}N_6O_2Si^+$: 397.2742, found 397.2733.

cis isomer (2S): brown oil; R_f = 0.56 [Et_2O:EtOH 20:1, ninidrine]; ^{1}H-NMR ($CDCl_3$): δ = 4.75 – 4.63 (m, 1H), 4.59 – 4.49 (m, 2H), 3.70 – 3.61 (m, 4H), 3.55 (dd, *J* = 10.0, 5.9 Hz, 1H), 3.37 (tdd, *J* = 7.5, 5.8, 4.7 Hz, 1H), 2.89 (t, *J* = 6.5 Hz, 2H), 2.54 (dd, *J* = 6.8, 4.5 Hz, 2H), 2.47 (dd, *J* = 6.8, 4.5 Hz, 2H), 2.35 – 2.13 (m, 3H), 2.13 – 1.85 (m, 2H), 1.82 – 1.65 (m, 1H), 0.88 (s, 9H), 0.04 (s, 6H); ^{13}C-NMR ($CDCl_3$): δ = 156.69, 66.95, 66.33, 61.14, 57.90, 53.87, 52.86, 45.29, 31.31, 27.57, 26.04, 18.46, -5.20; t_r = 6.49 min; ESI-MS [M+H] 397.4; diastereoisomeric purity (HPLC) > 99.9%; HRMS (ESI+): calculated for $C_{18}H_{37}N_6O_2Si^+$: 397.2742, found 397.2718.

4a – *trans* isomer

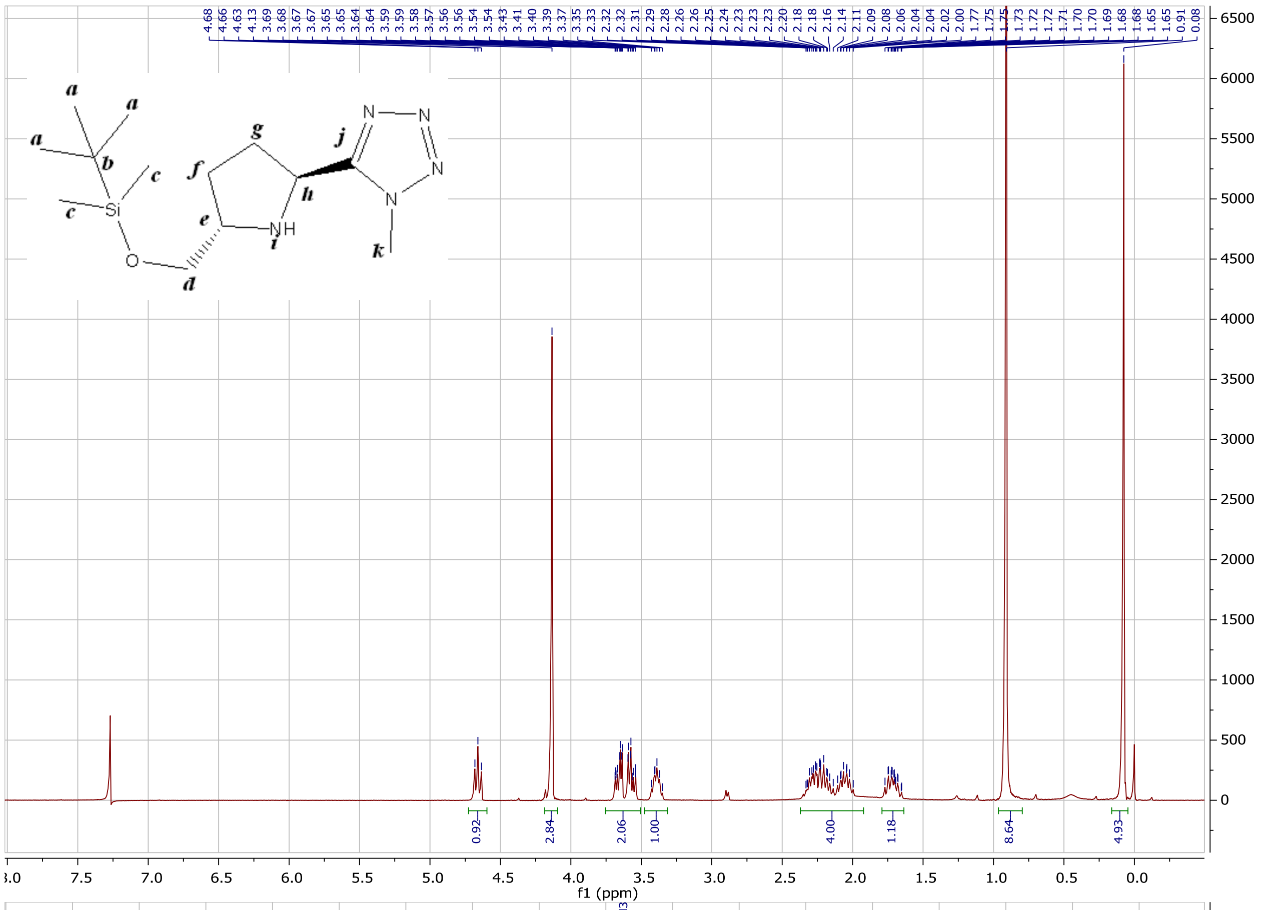

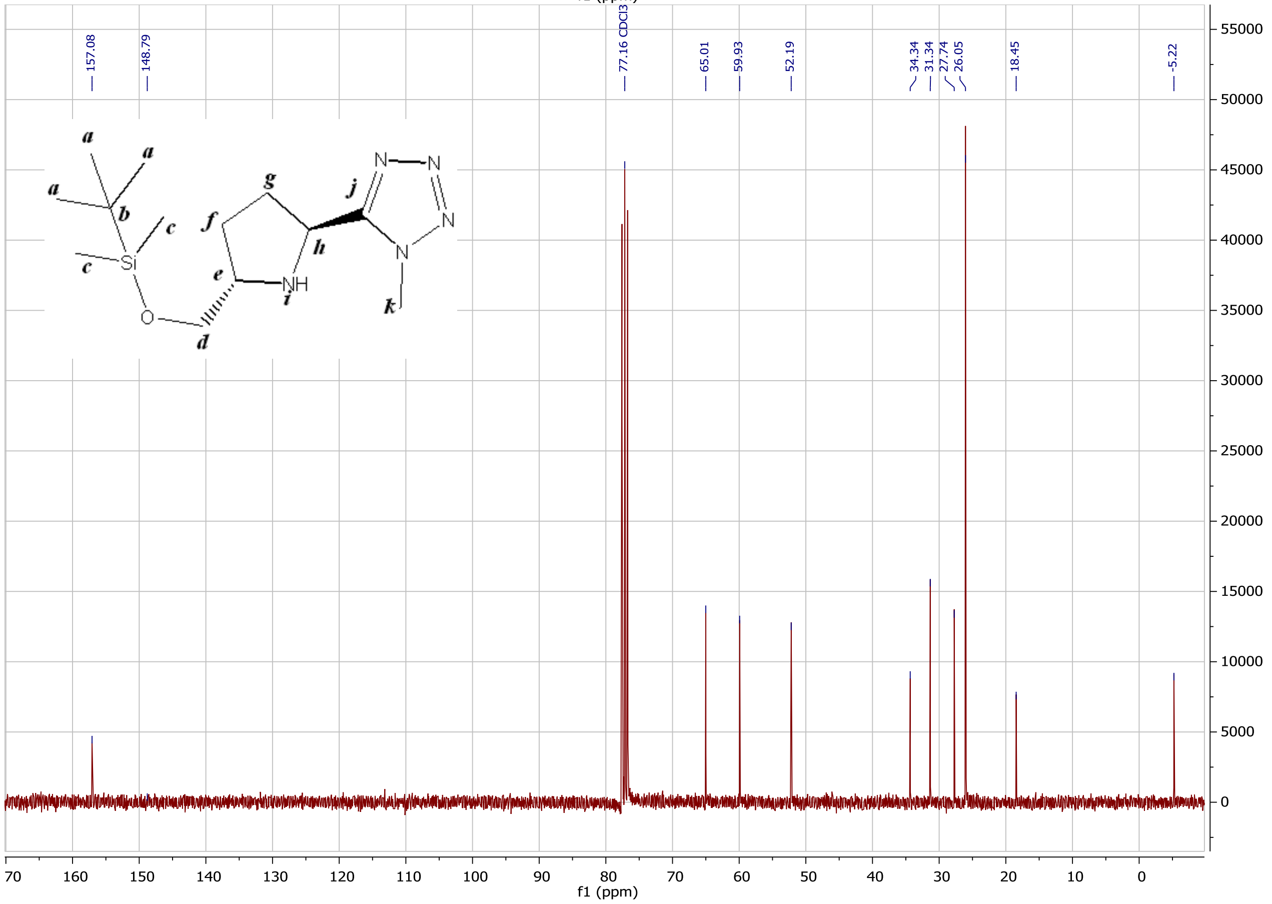

4a – *cis* isomer

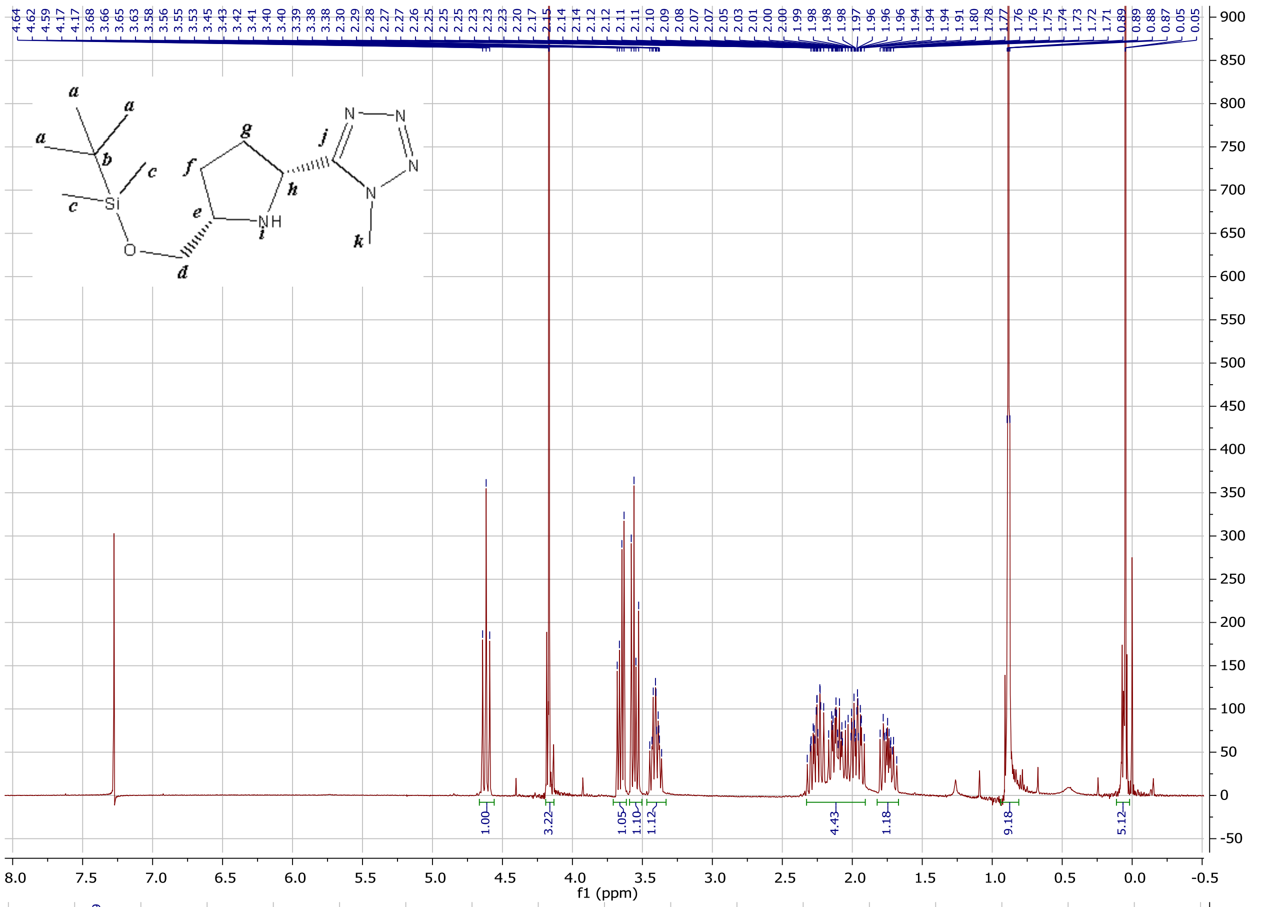

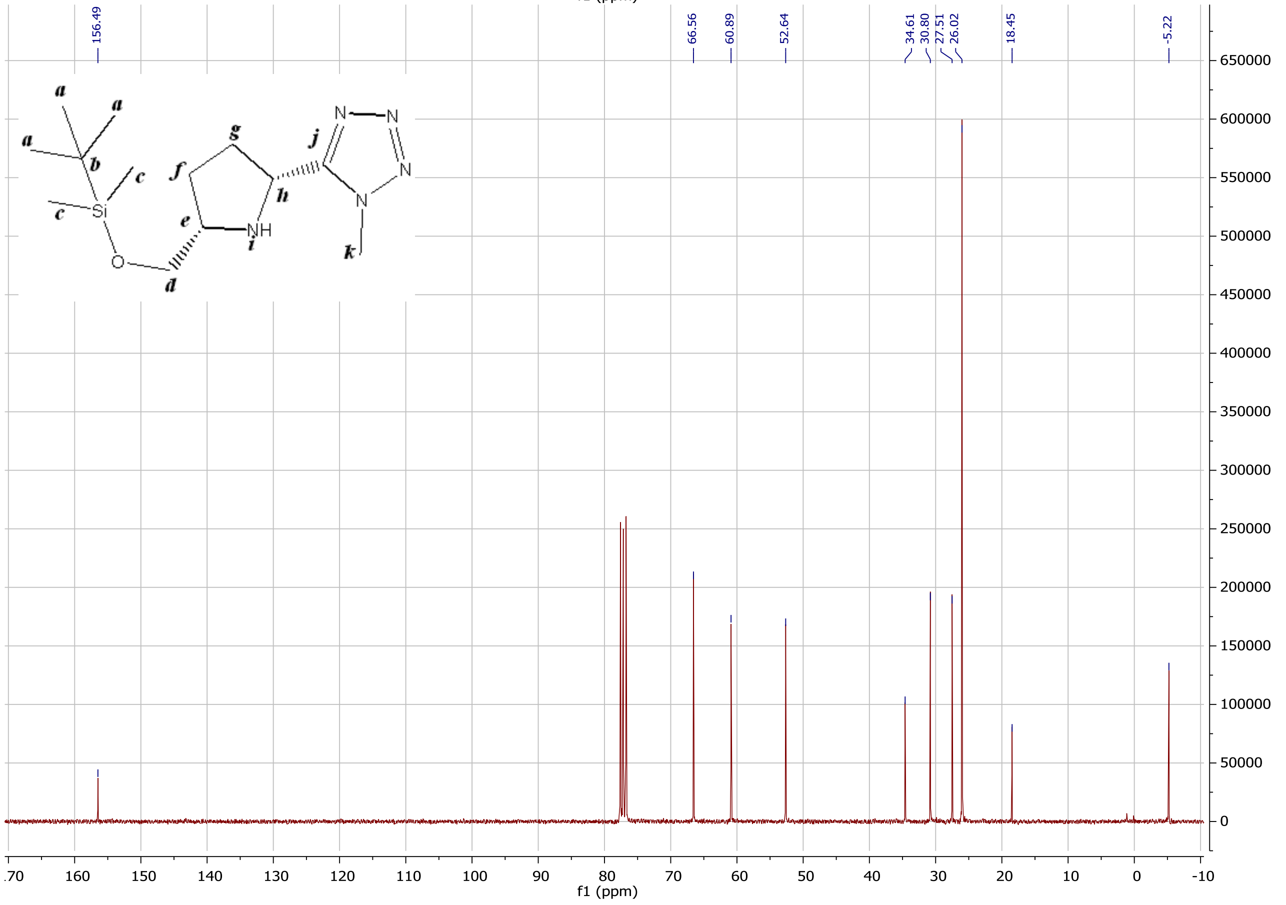

4a – NOESY 2D of *trans* and *cis* isomers

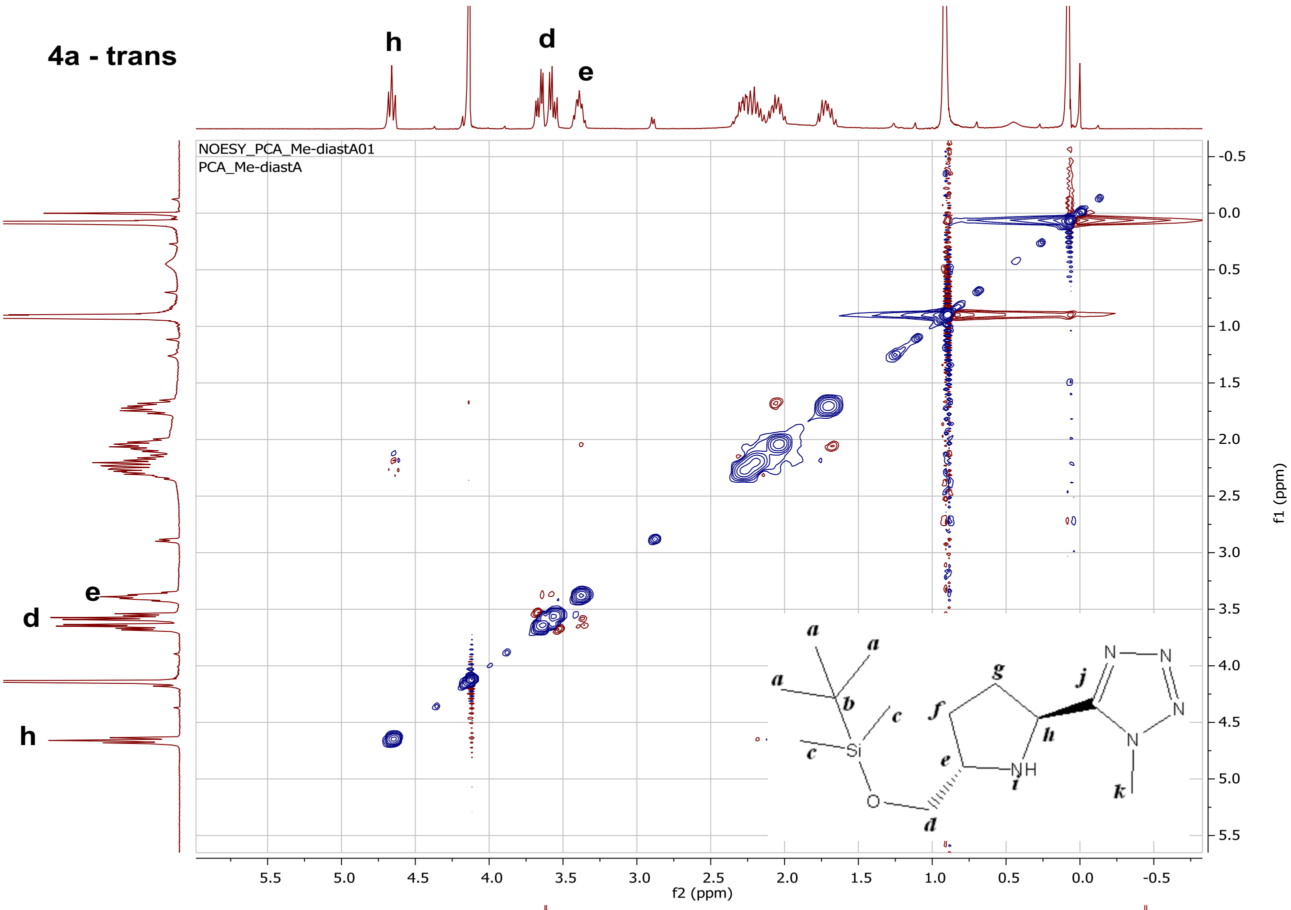

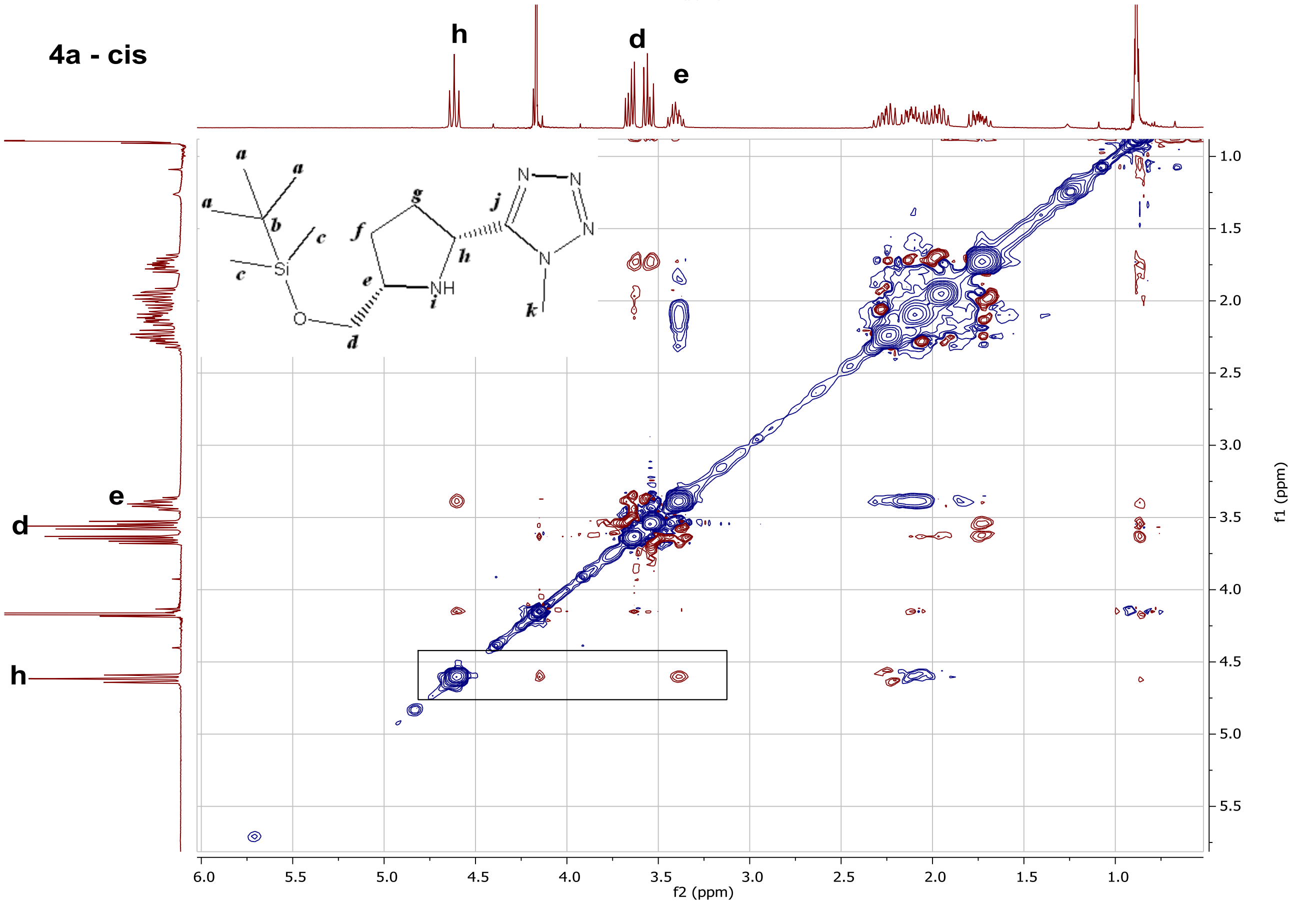

4b – *trans* isomer

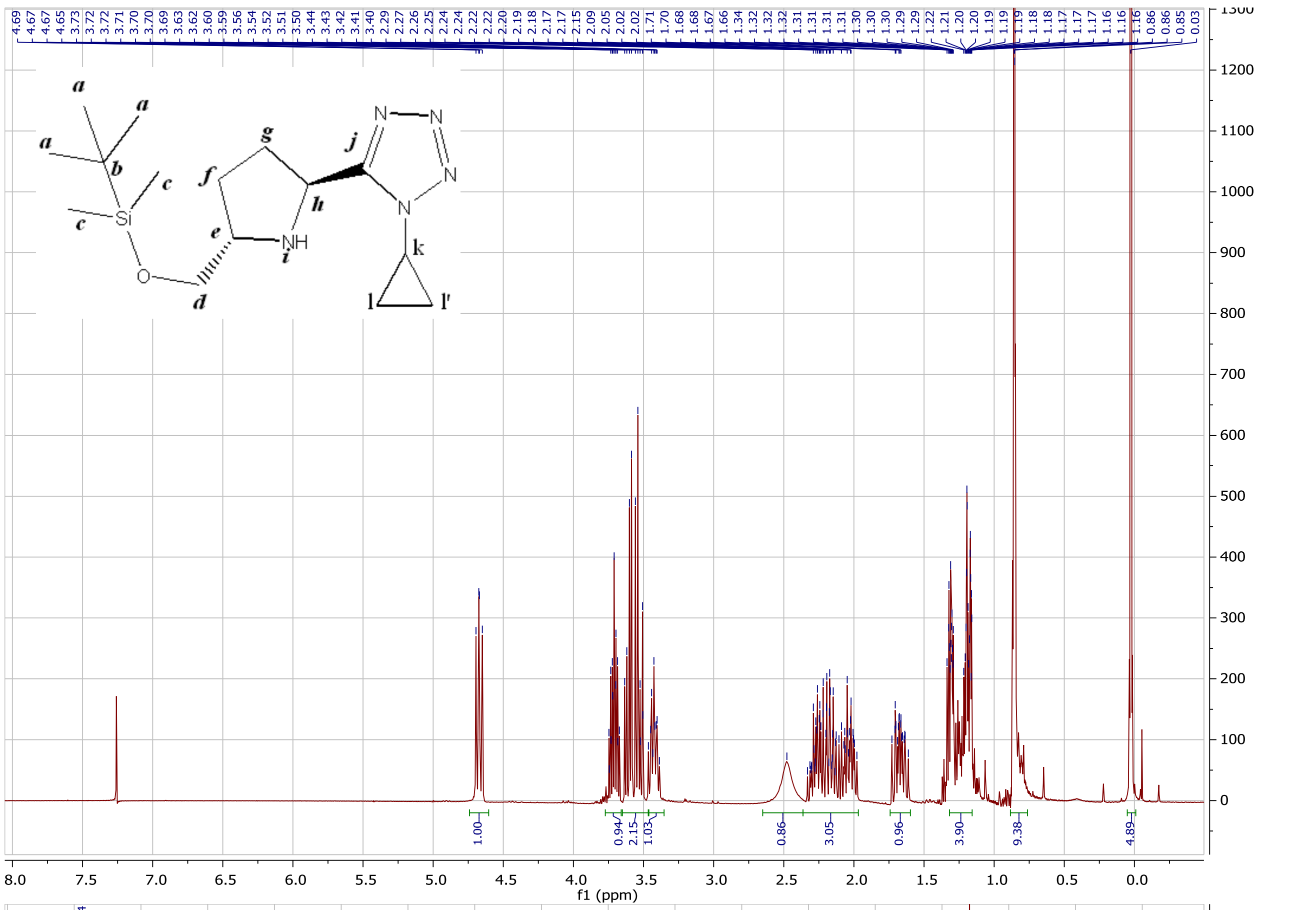

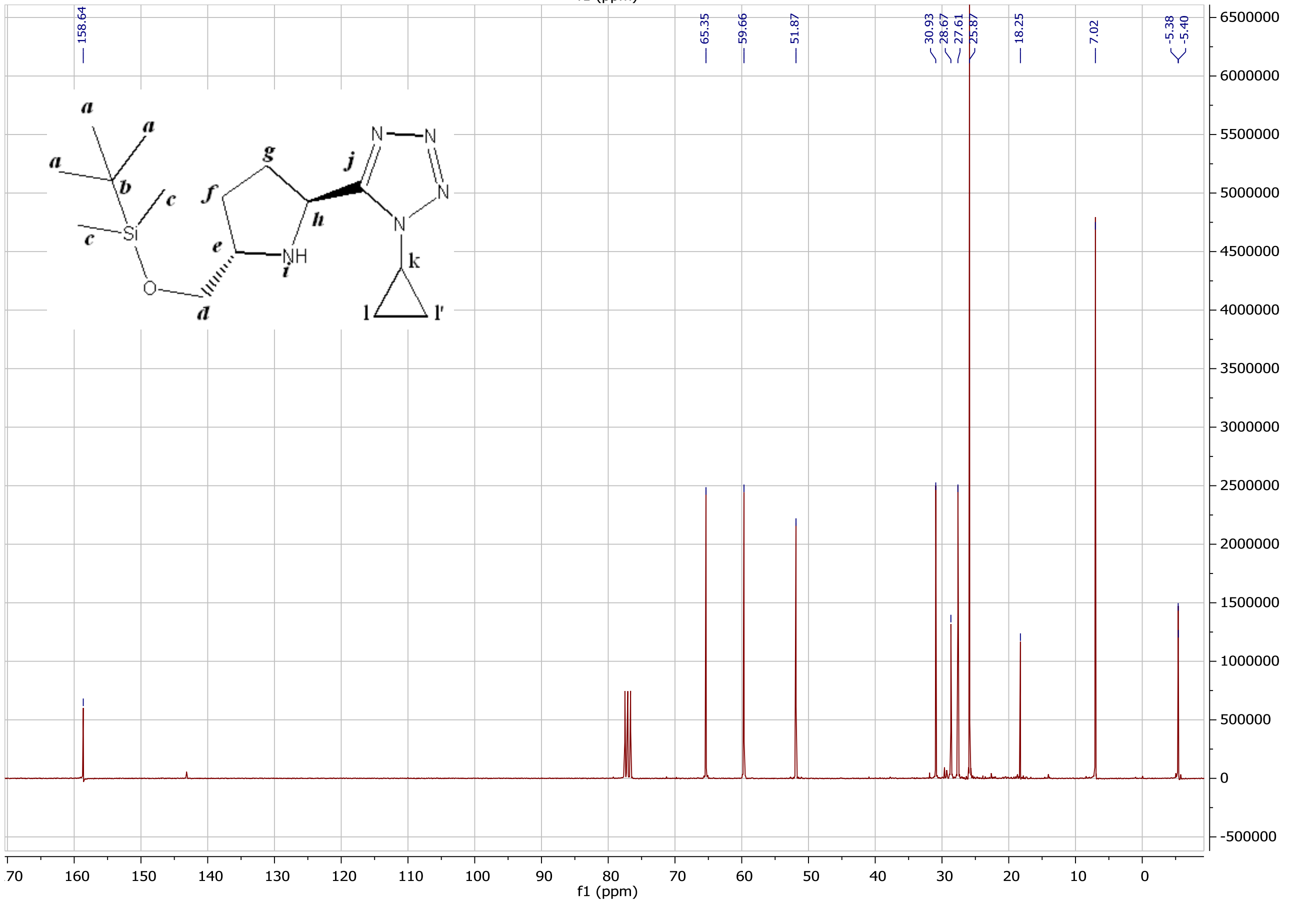

4b – *cis* isomer

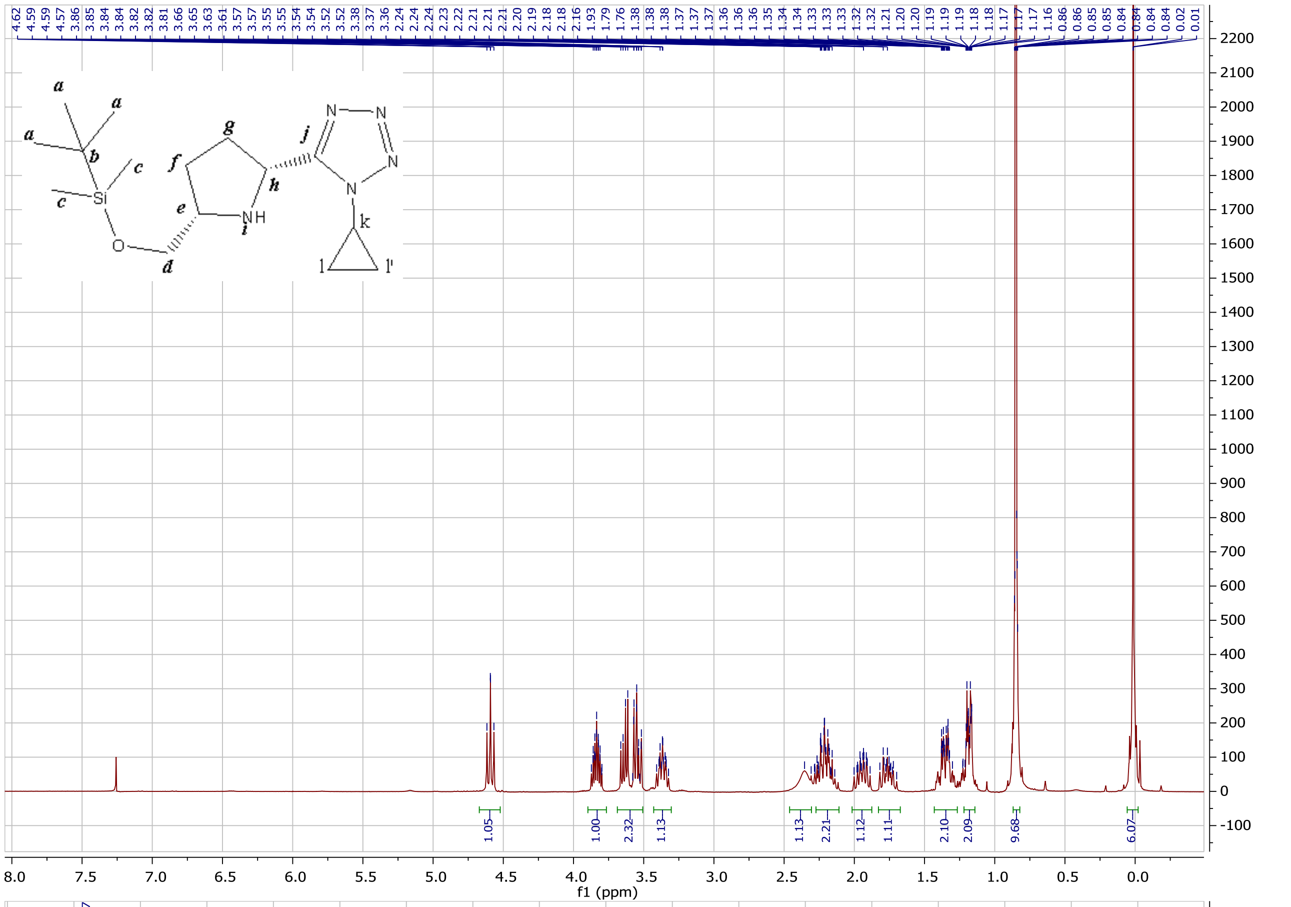

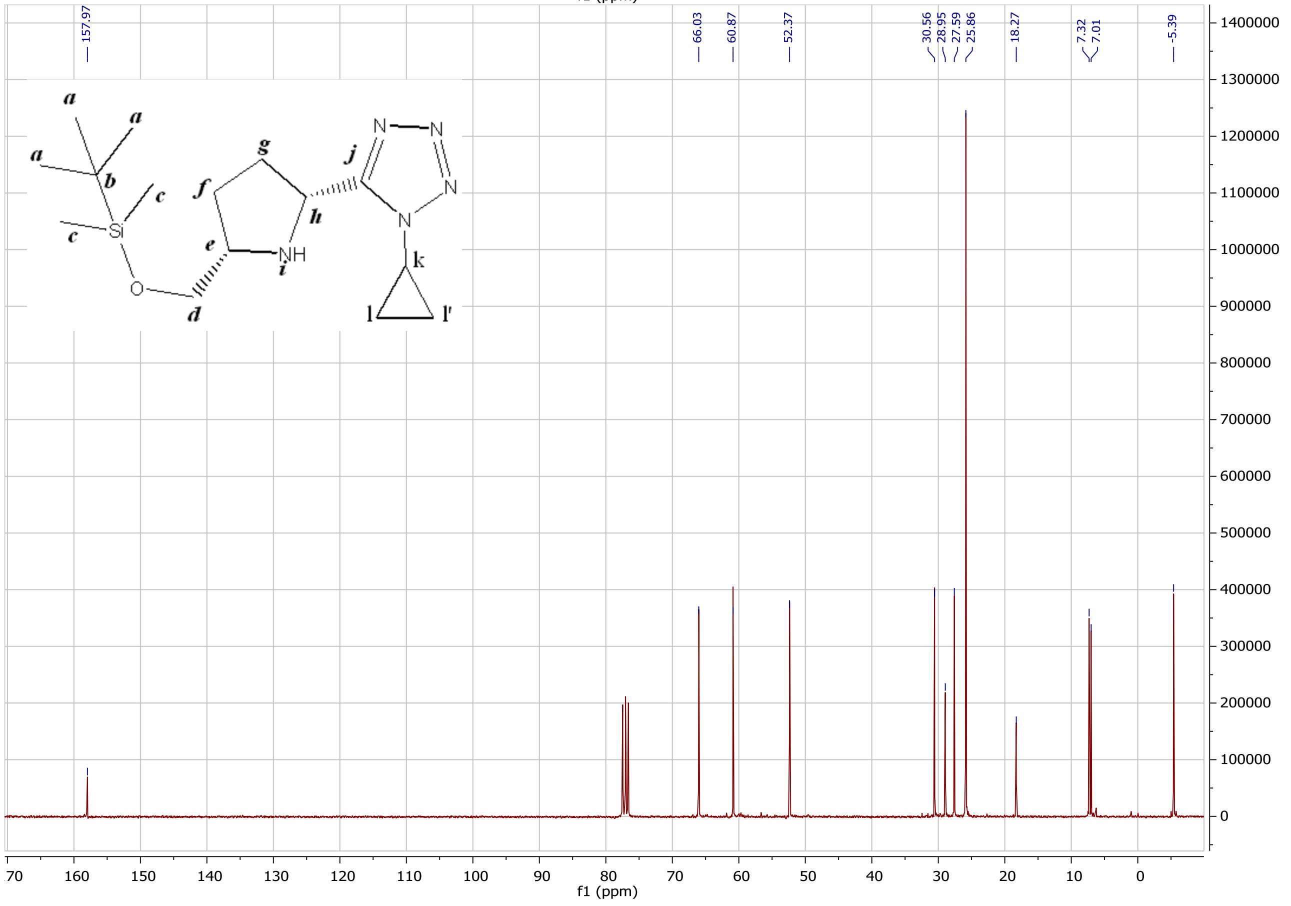

4b – NOESY 2D of *trans* and *cis* isomers

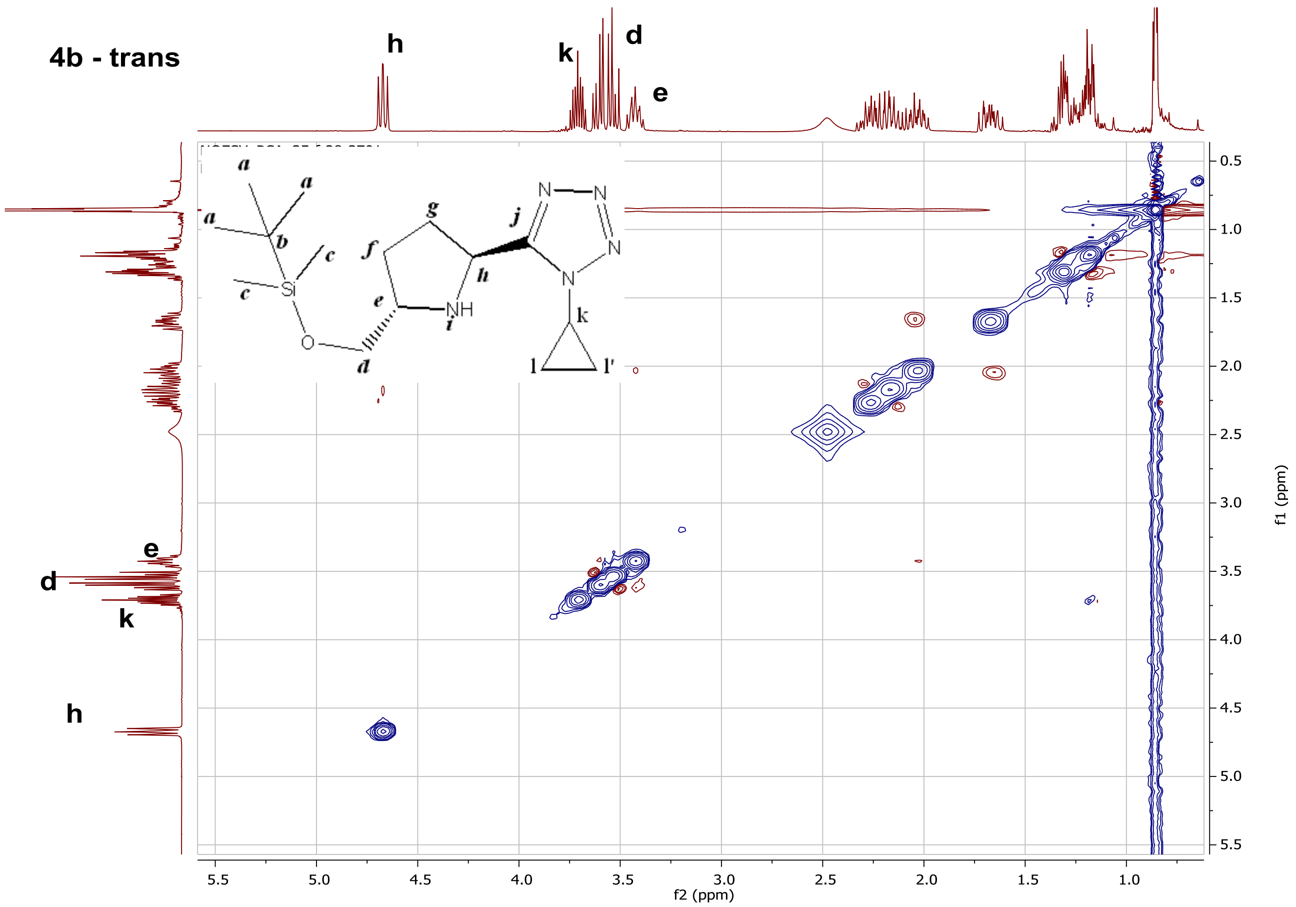

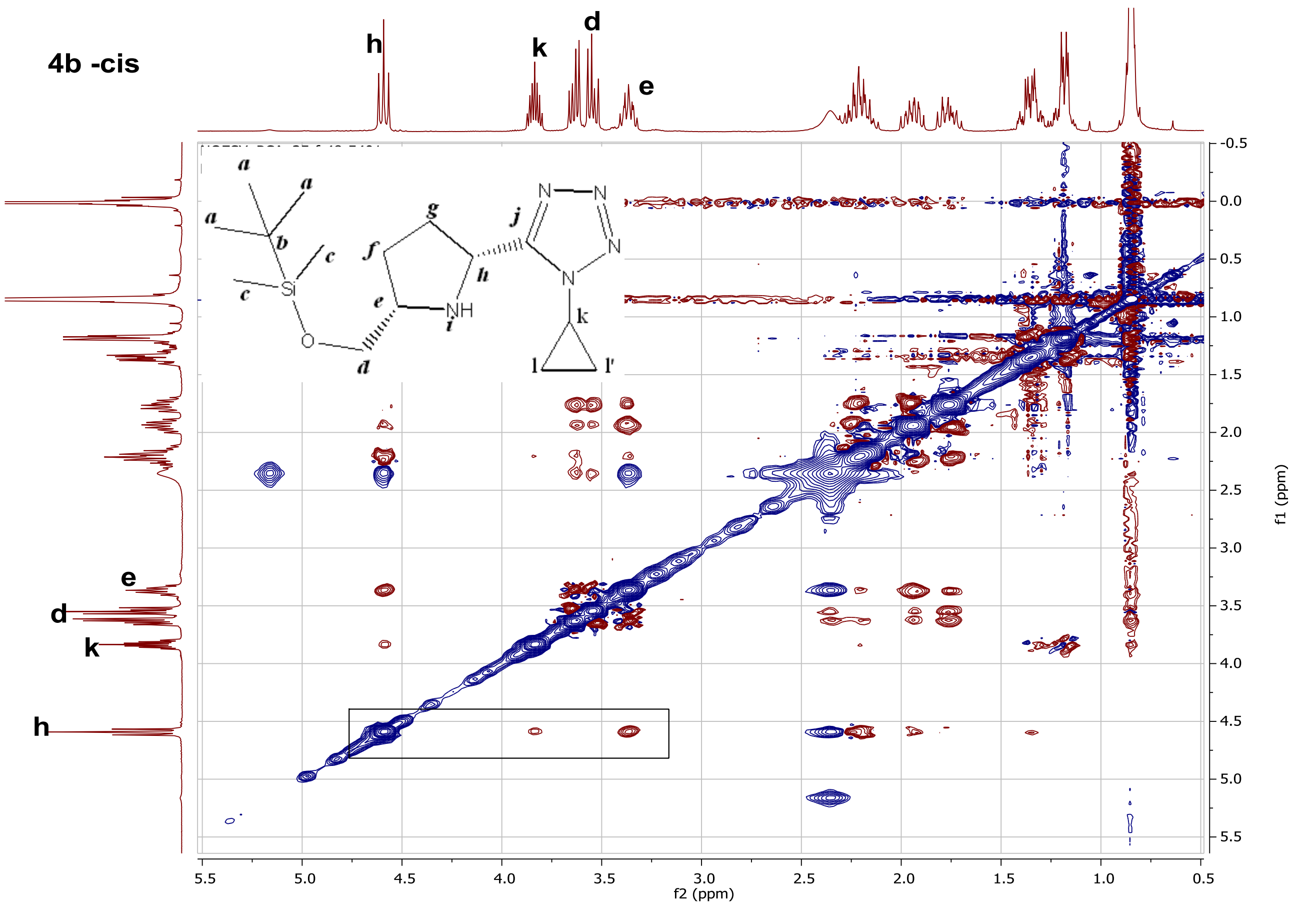

4c – *trans* isomer

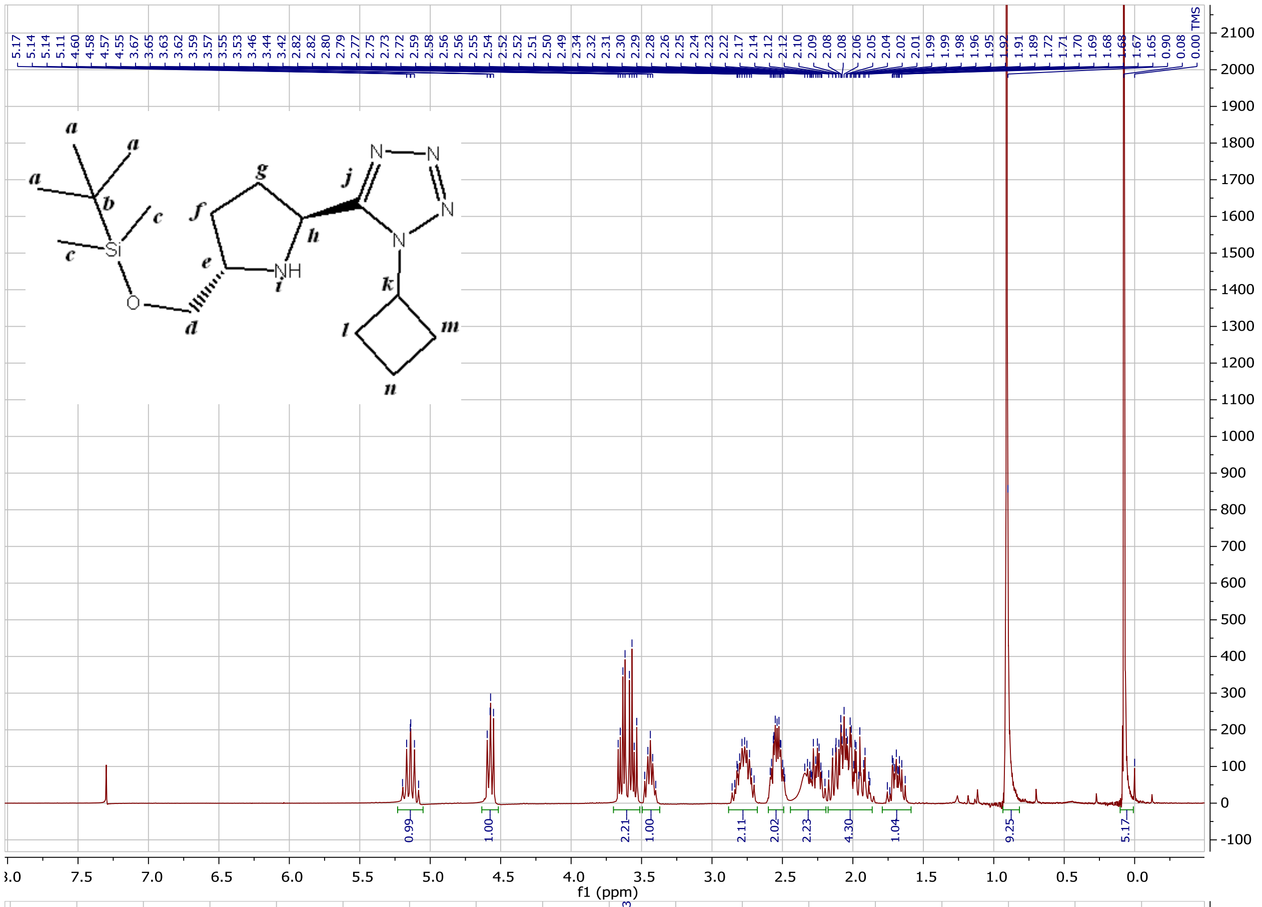

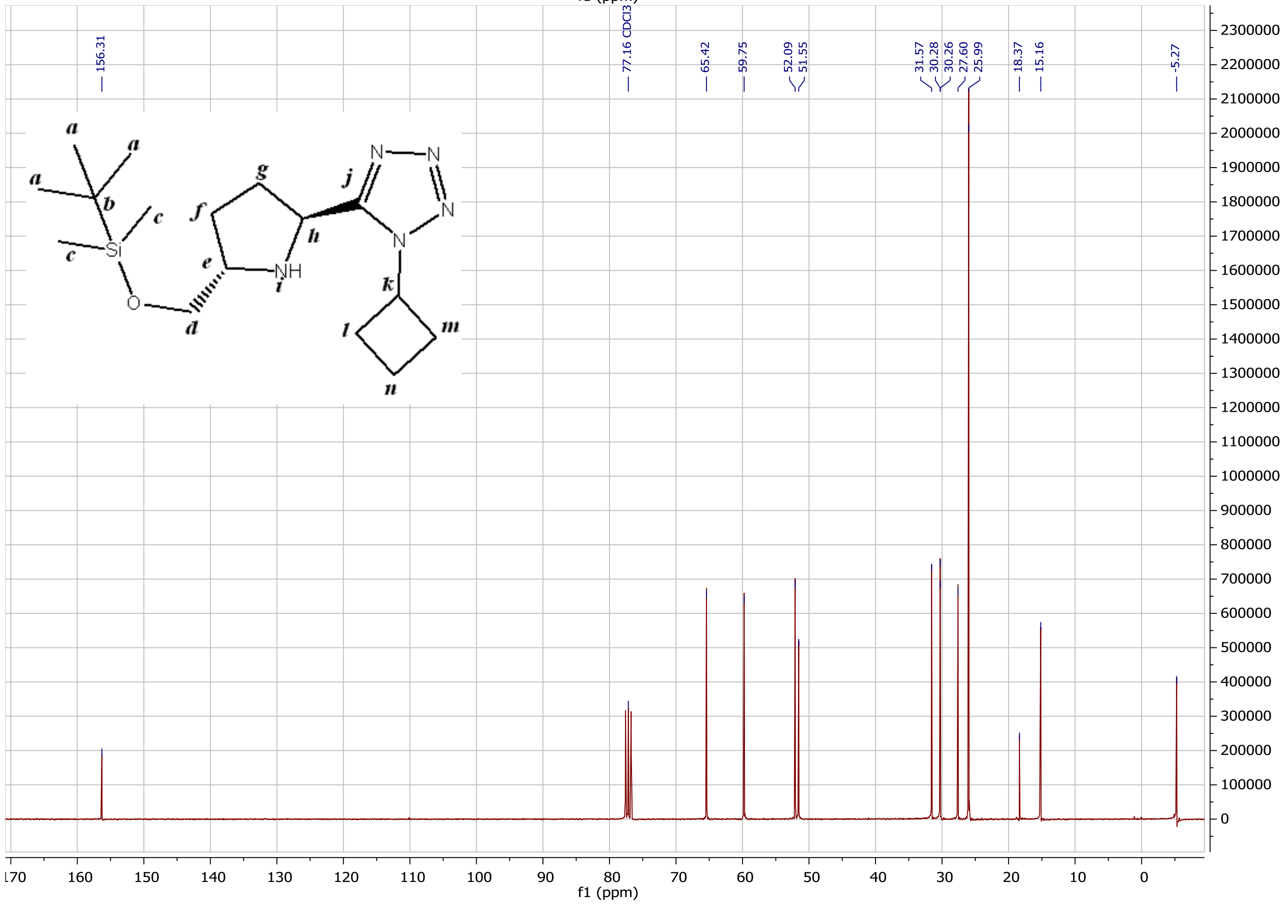

4c – *cis* isomer

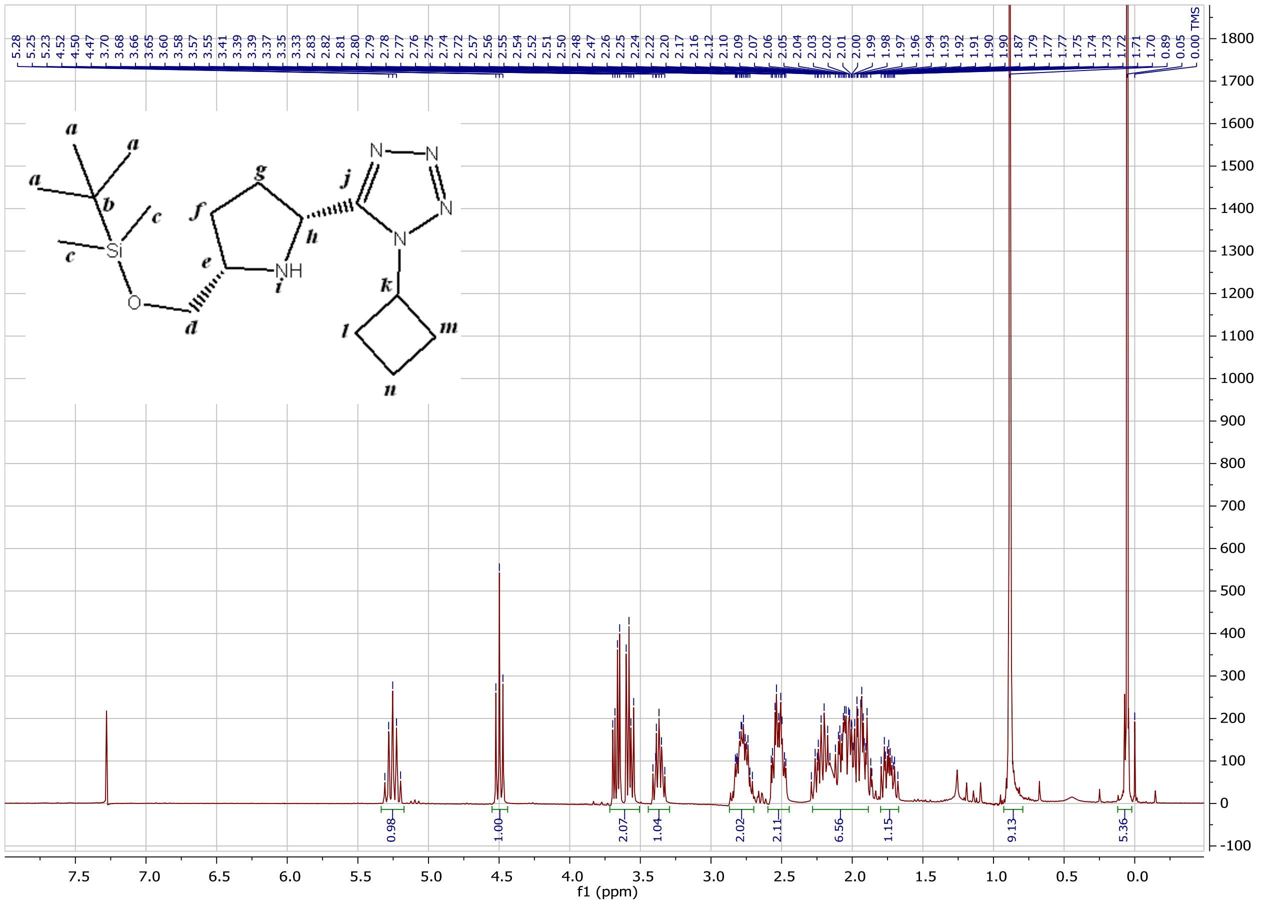

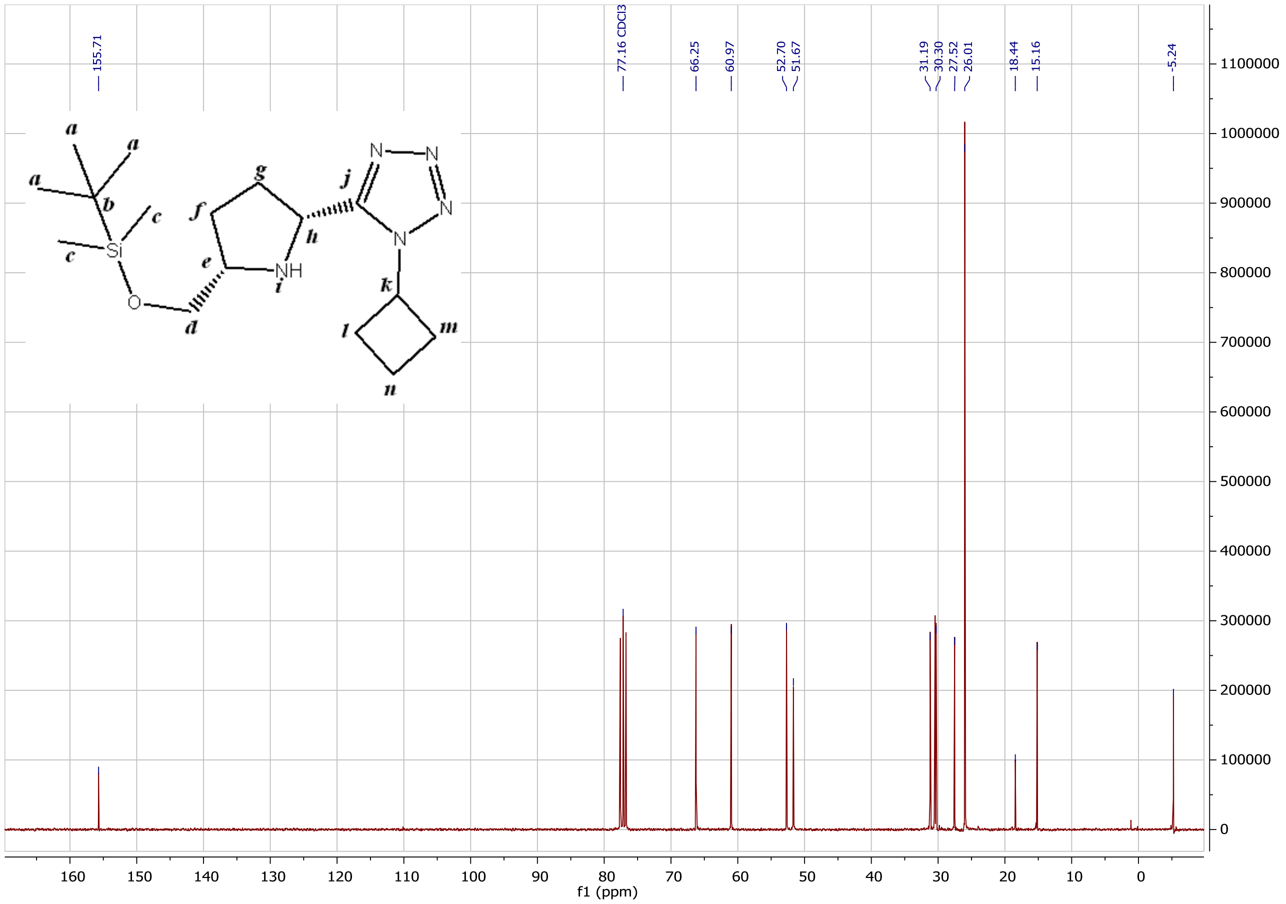

4c – NOESY 2D of *trans* and *cis* isomers

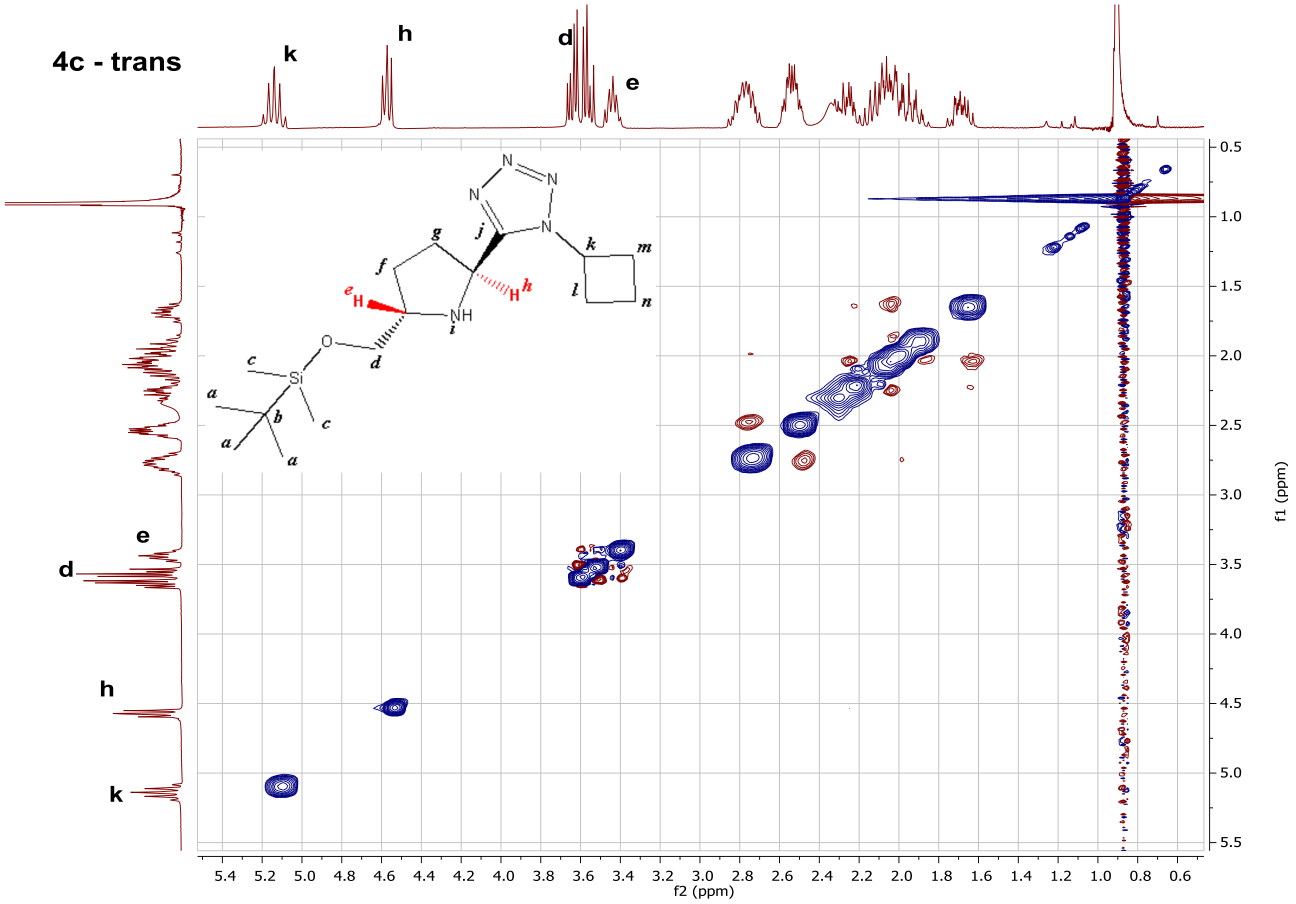

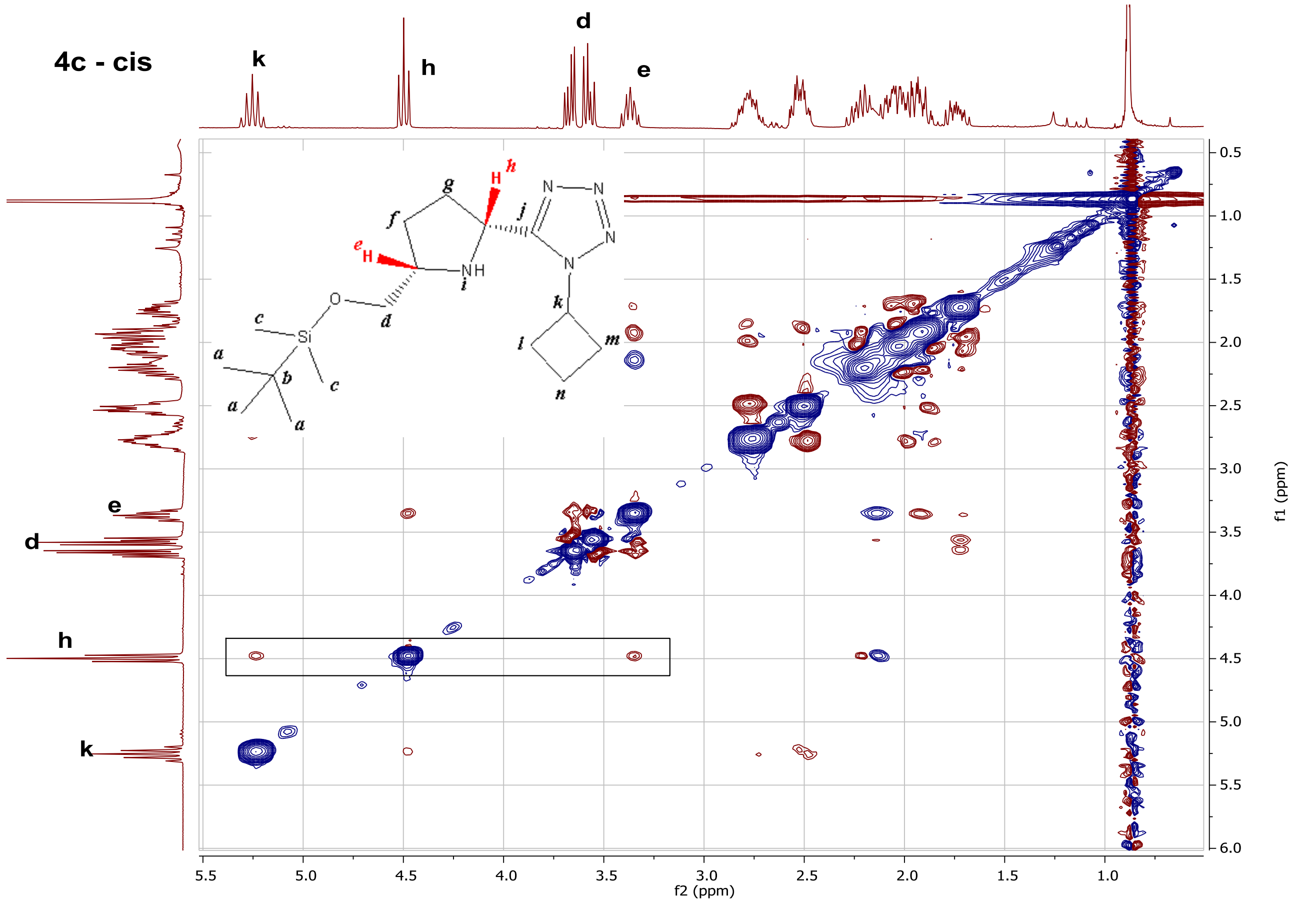

4d – *trans* isomer

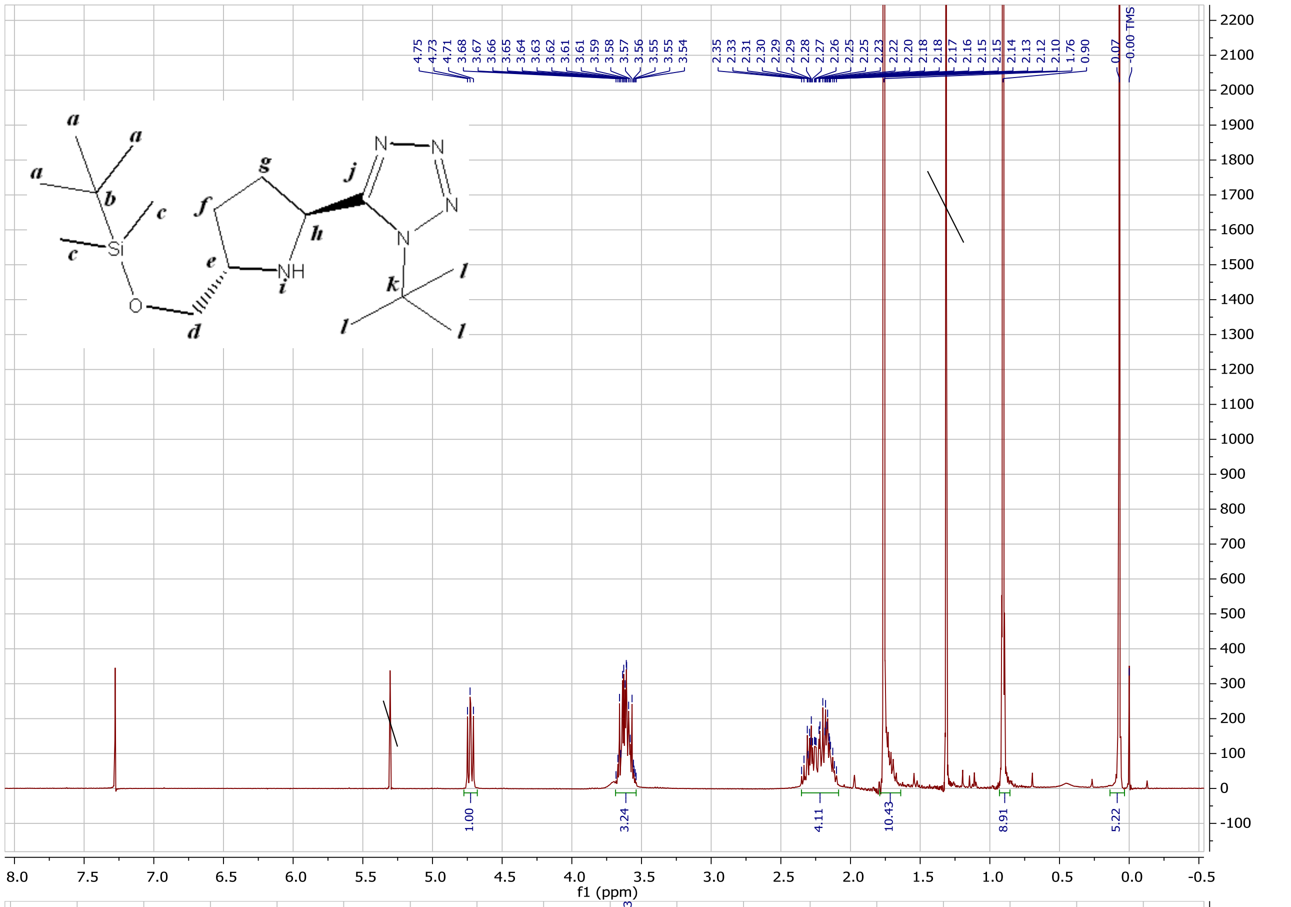

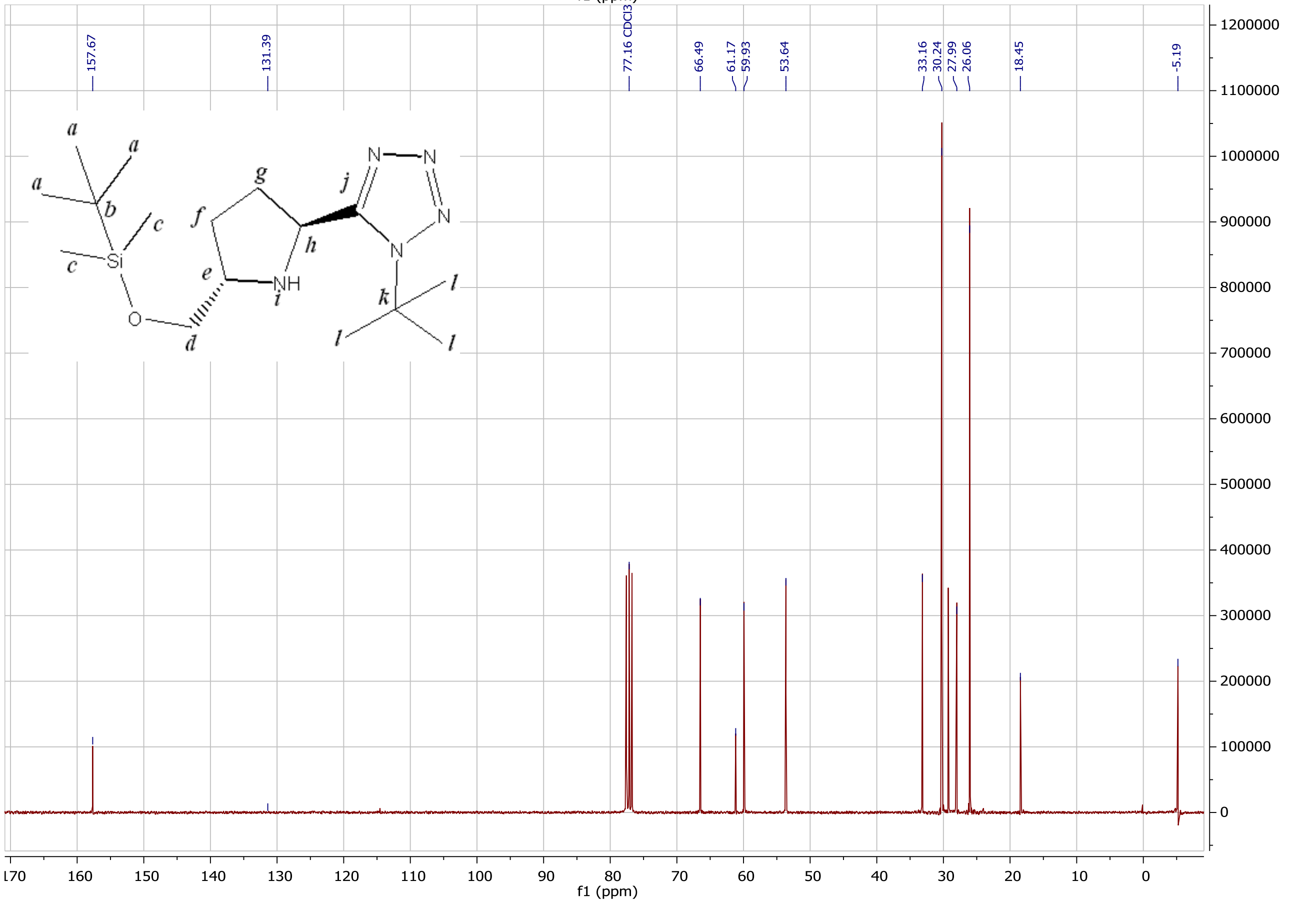

4d – *cis* isomer

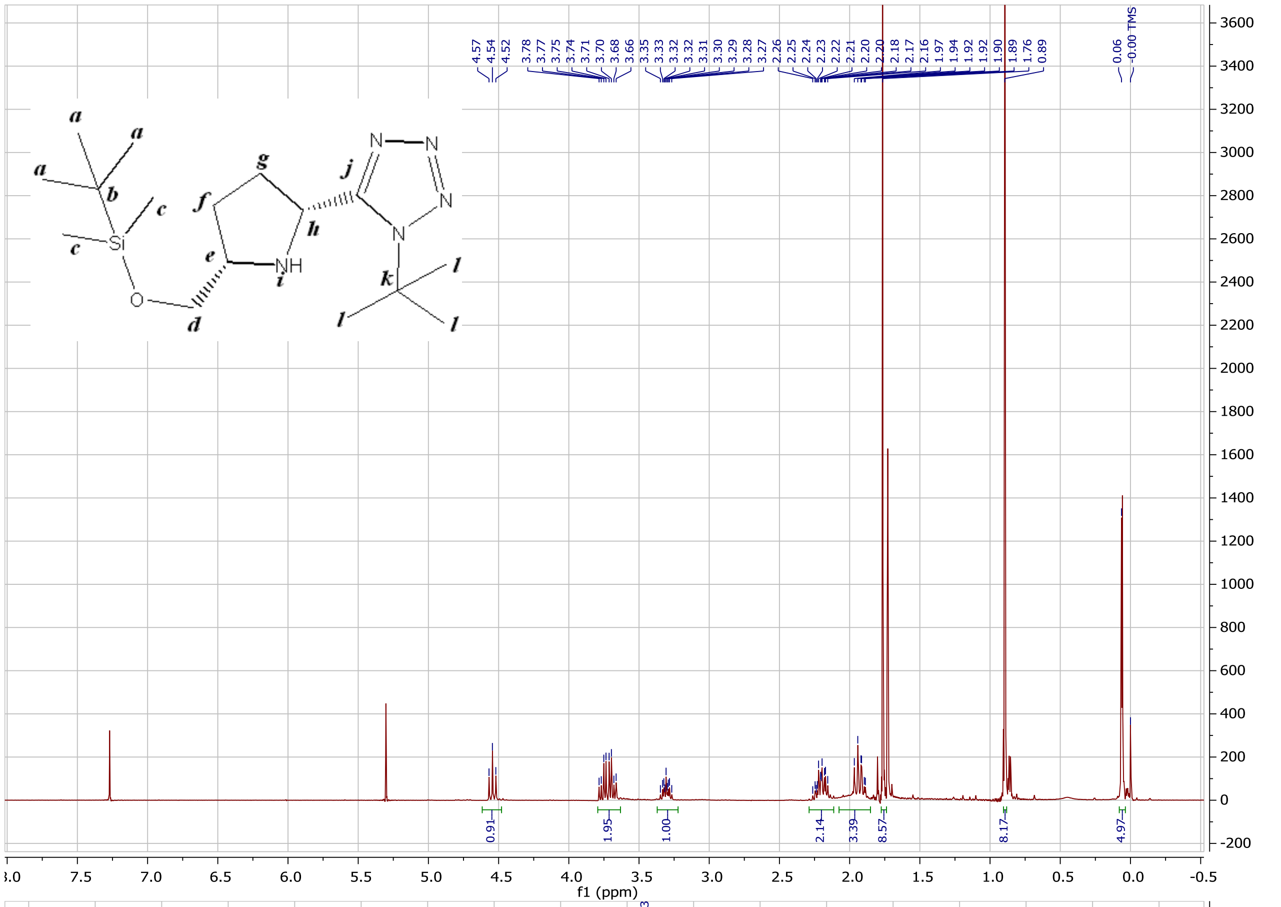

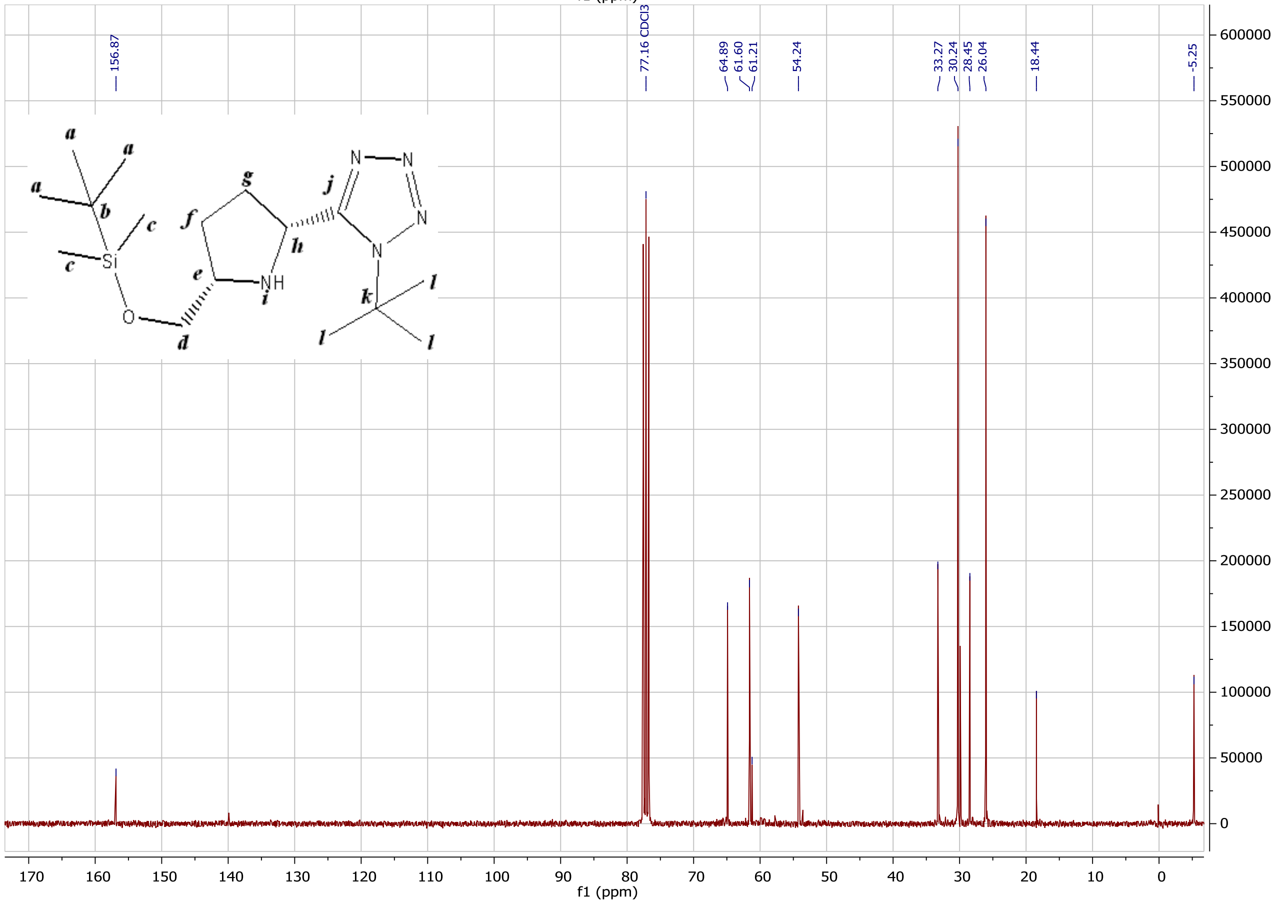

4d – NOESY 2D of *trans* and *cis* isomers

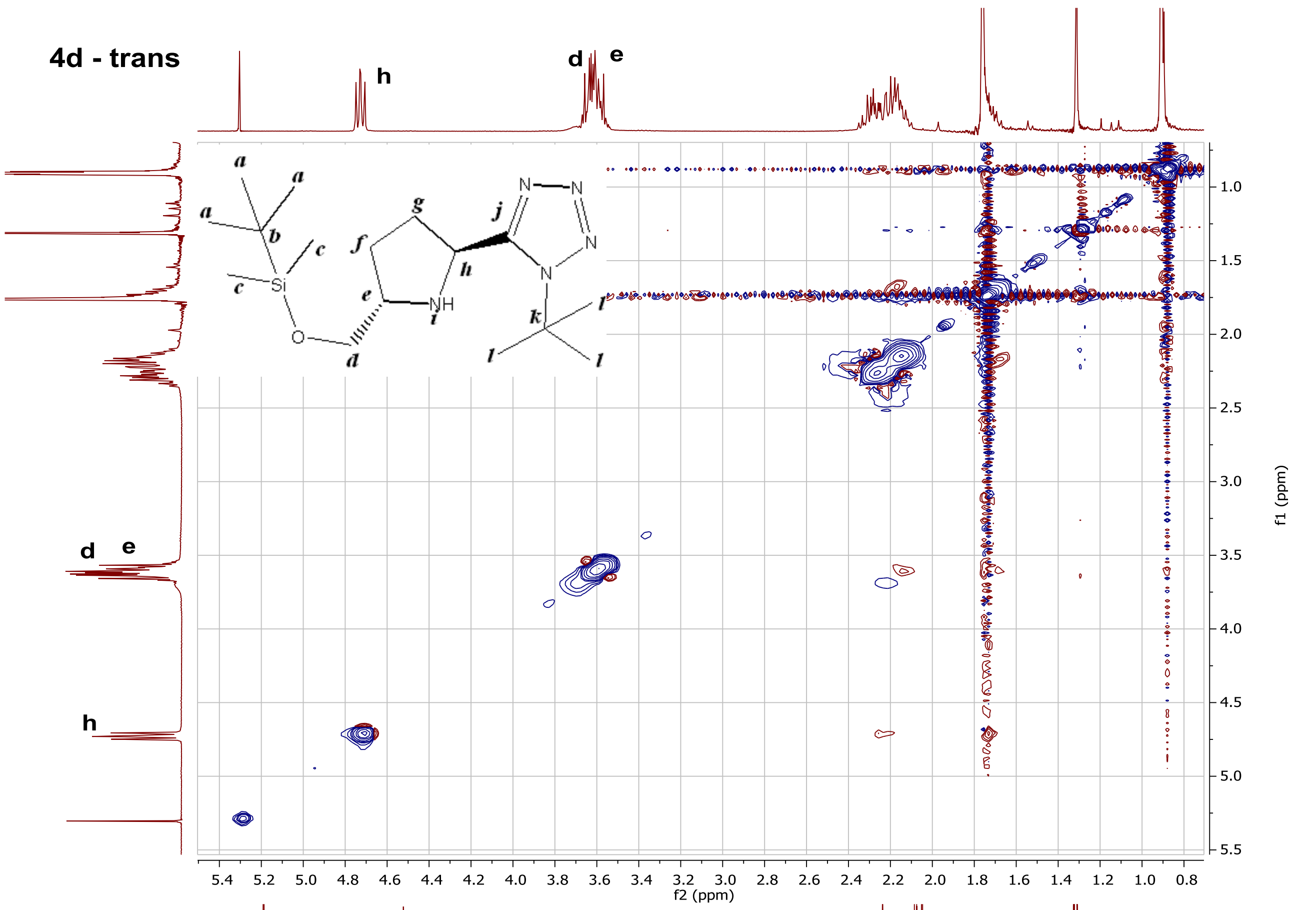

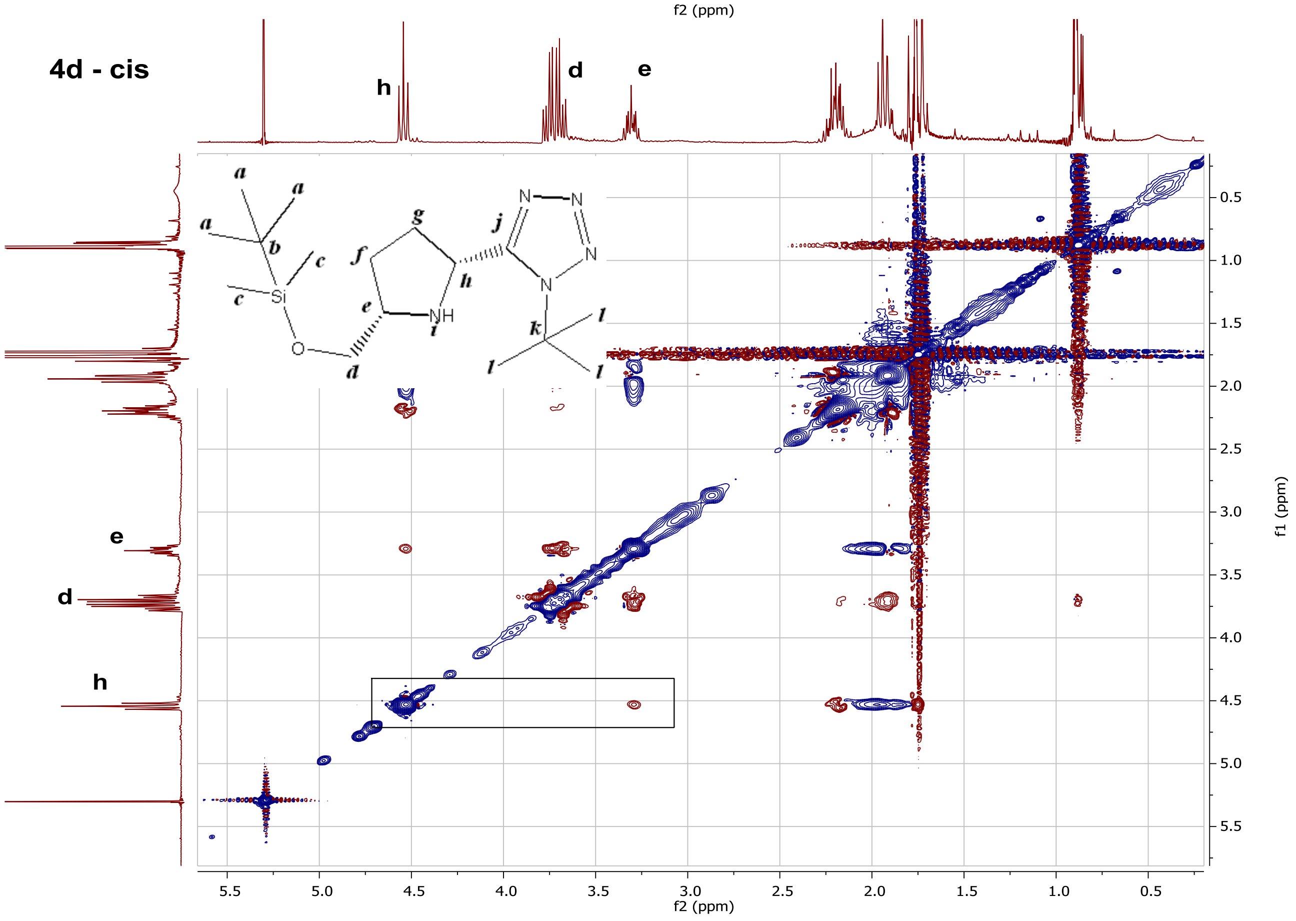

4e – *trans* isomer

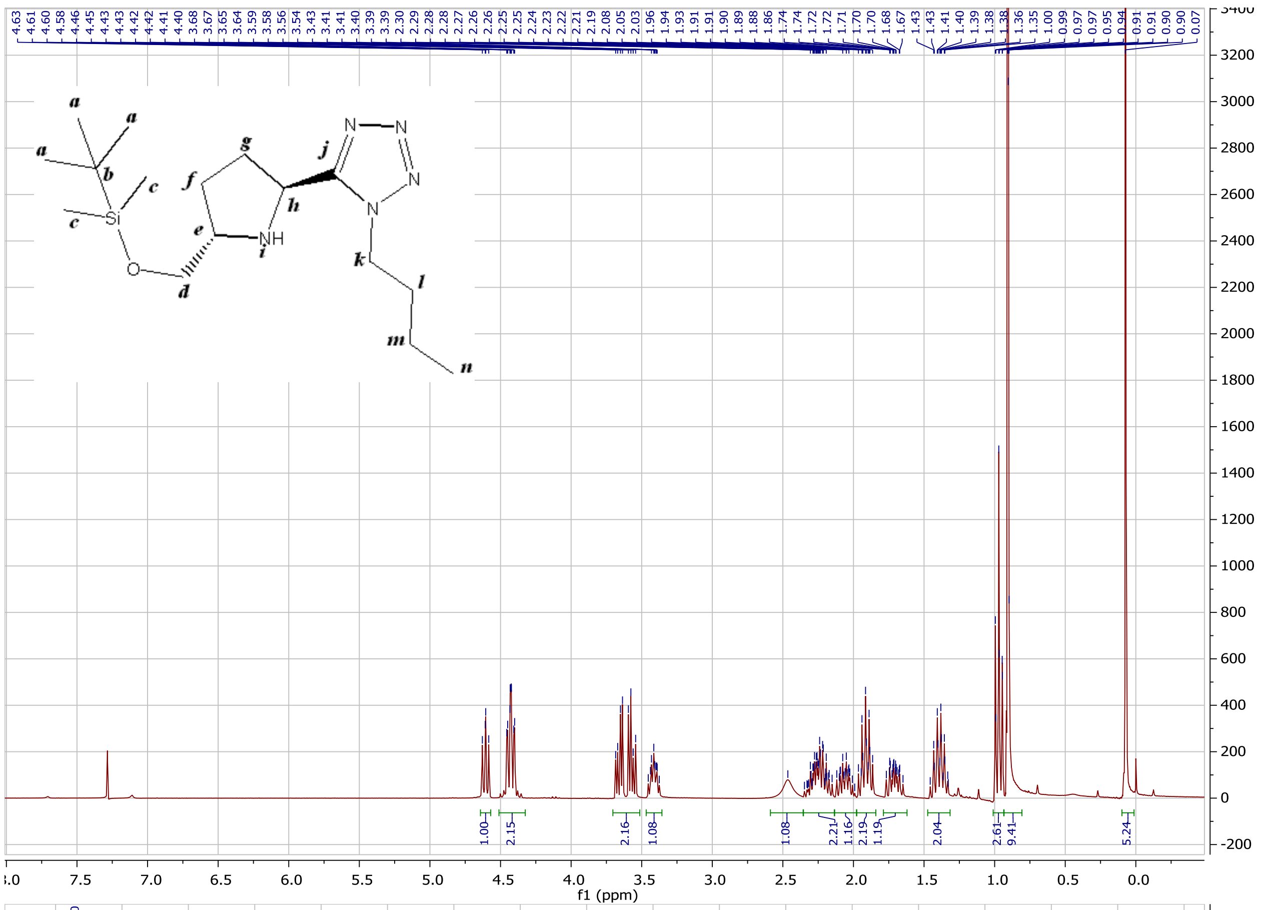

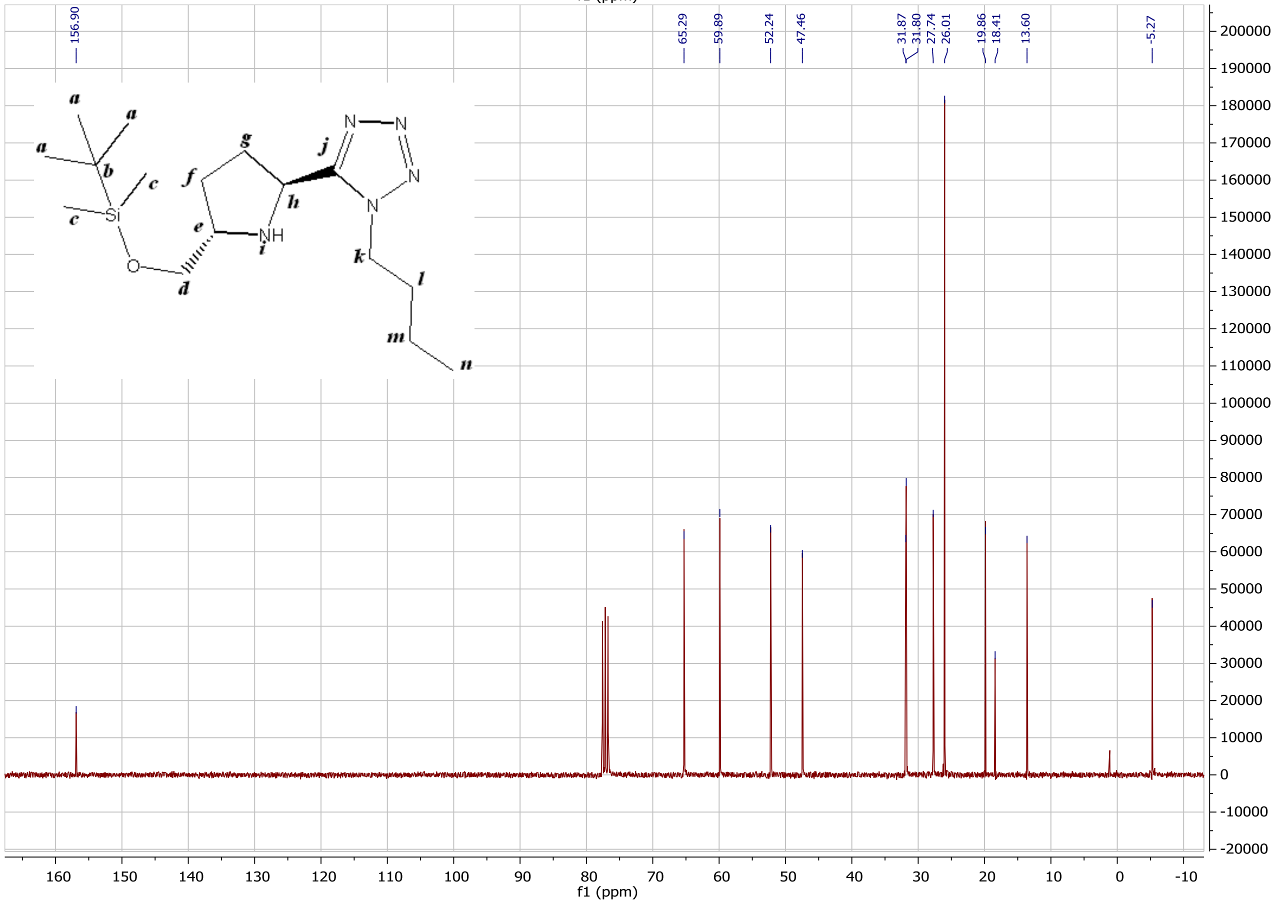

4e – *cis* isomer

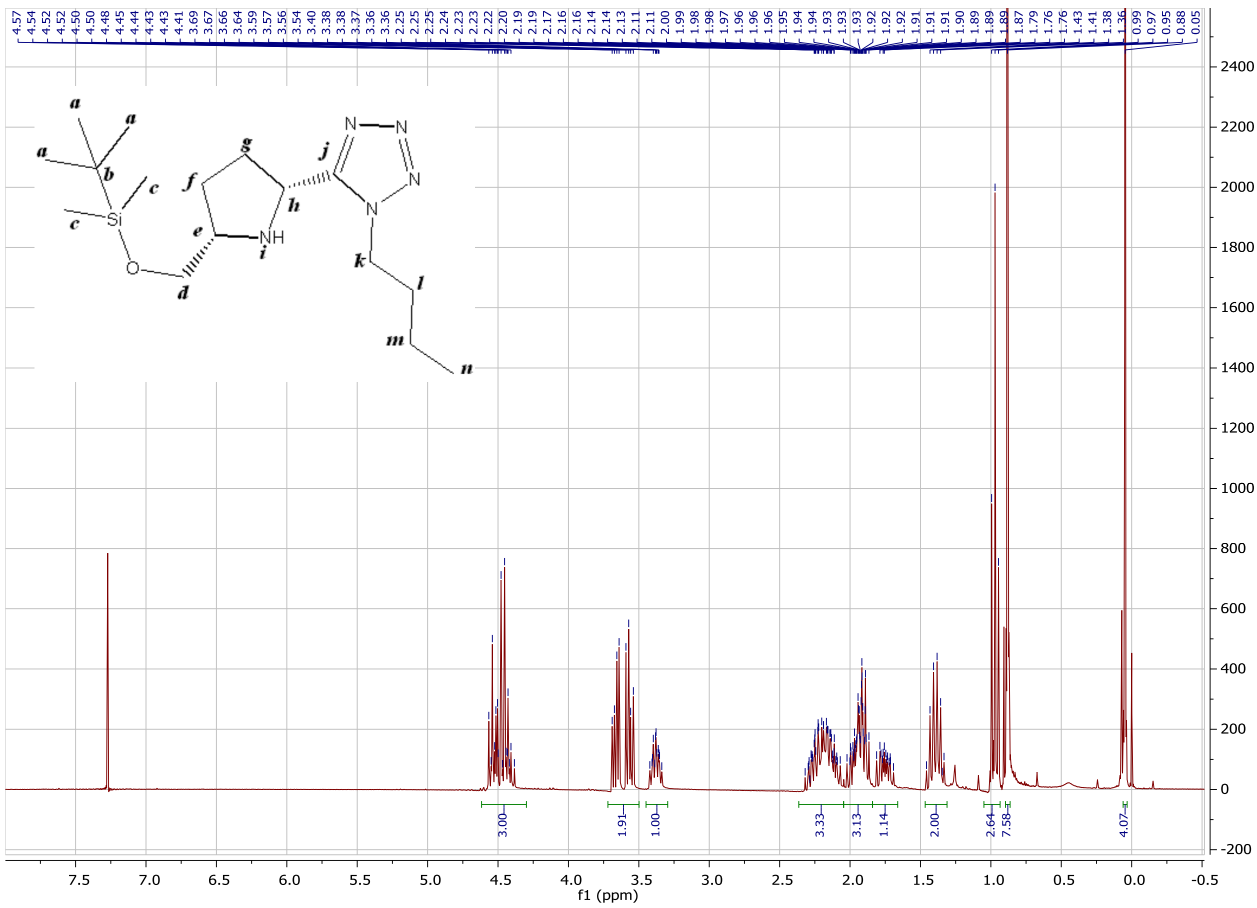

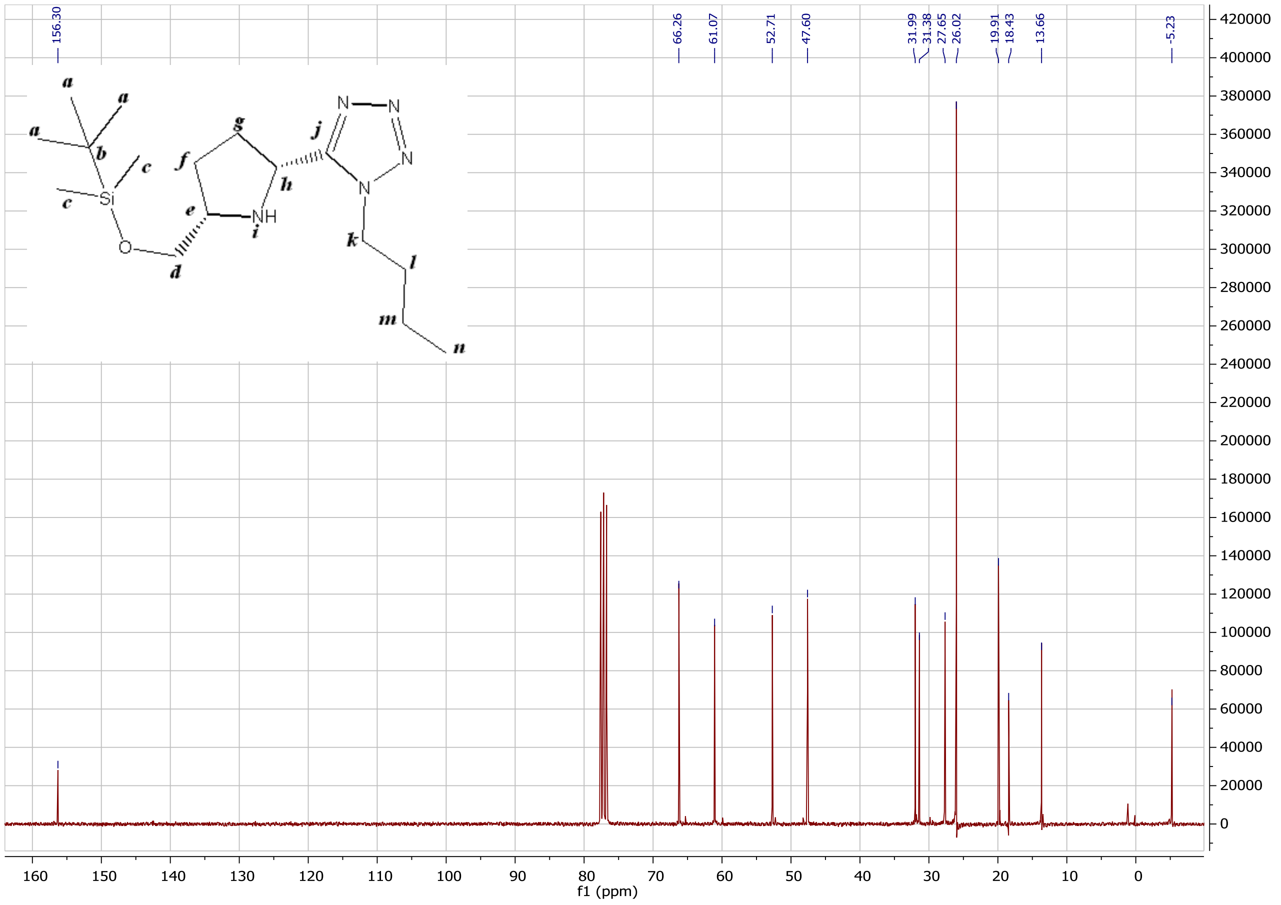

4e – NOESY 1D of *trans* and *cis* isomers

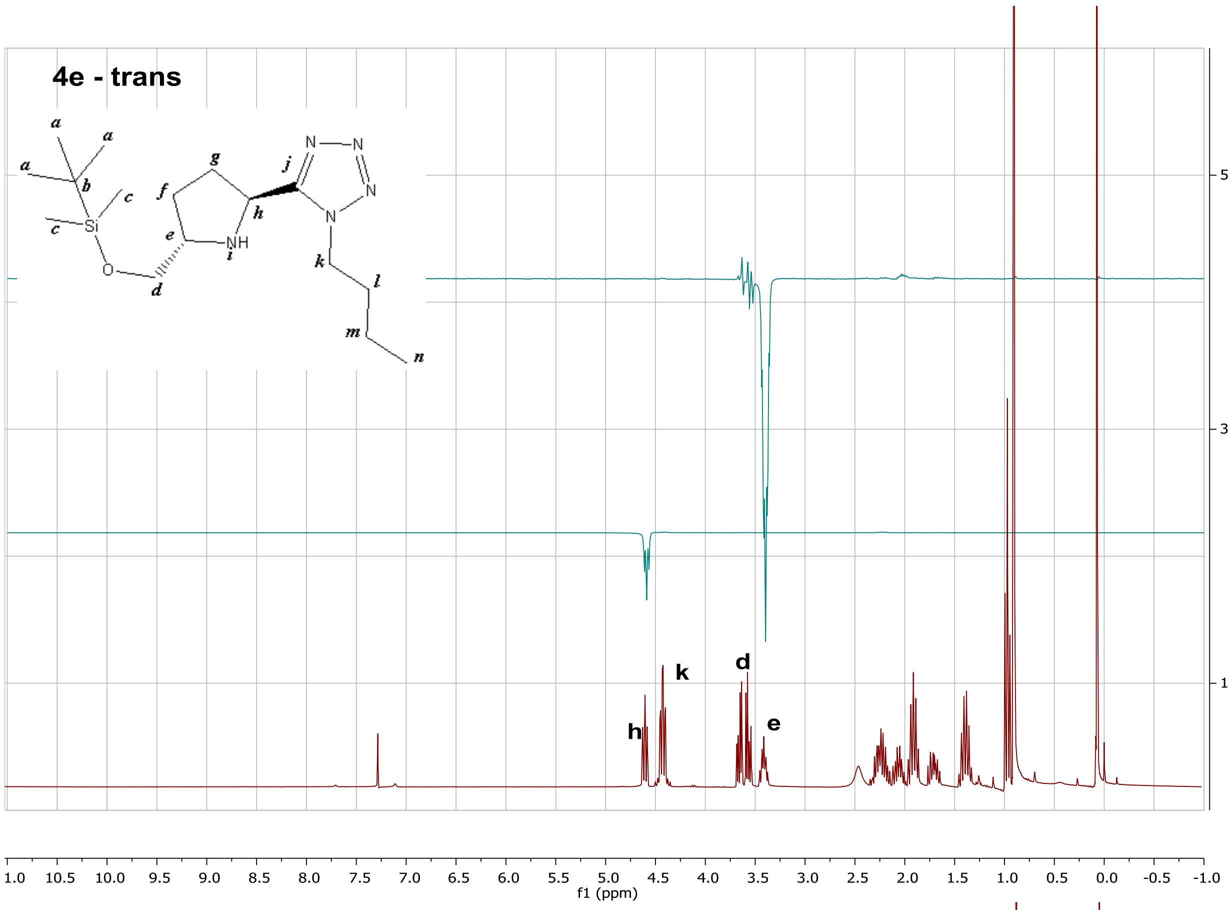

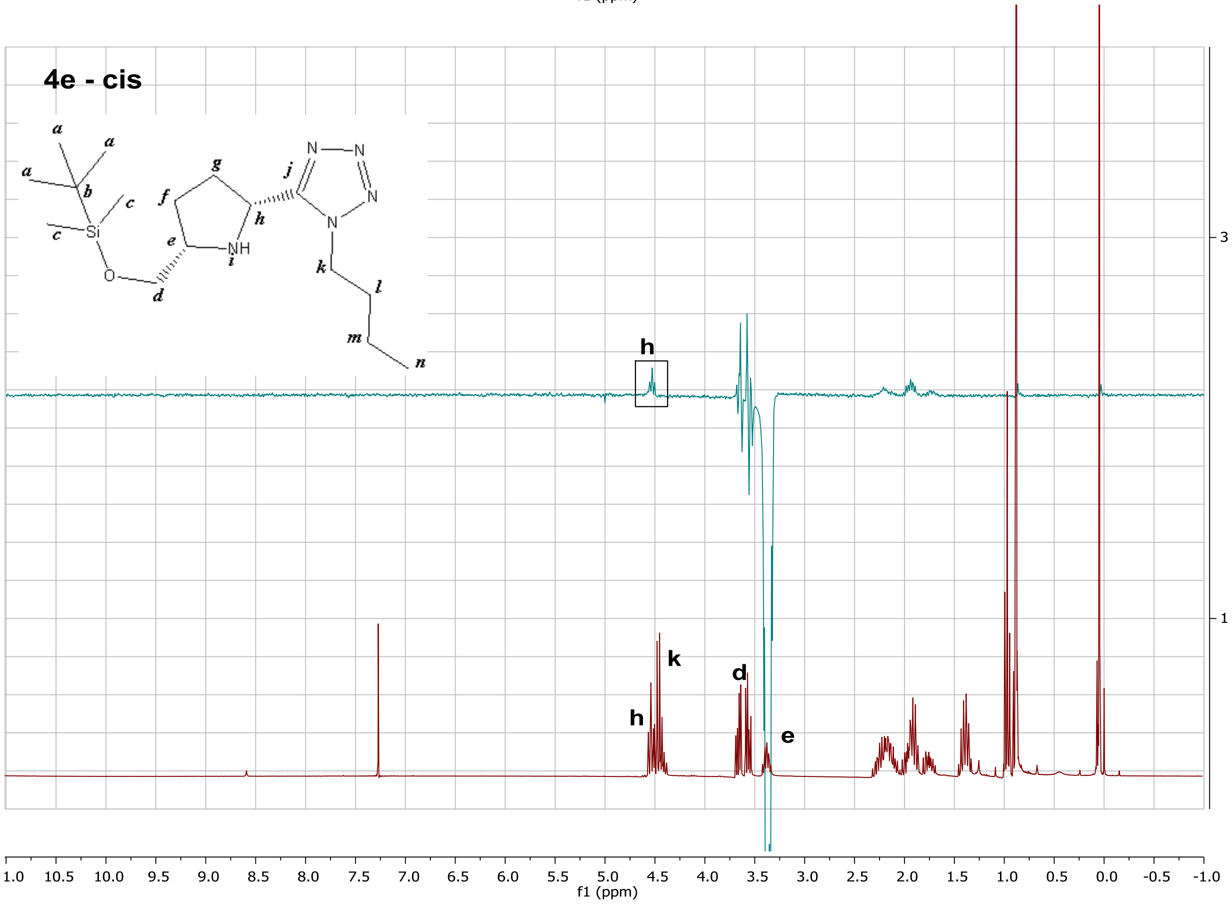

4f – *trans* isomer

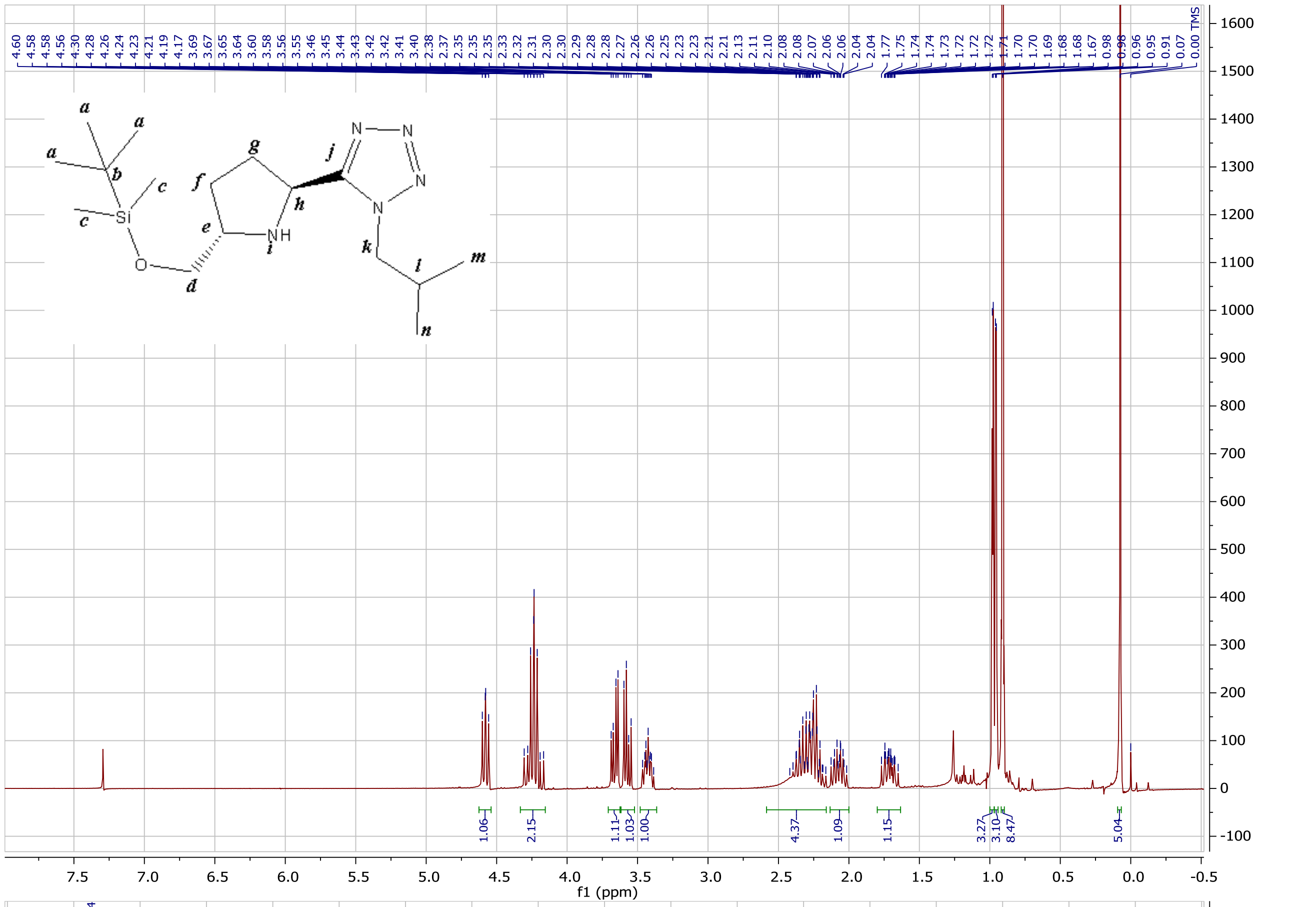

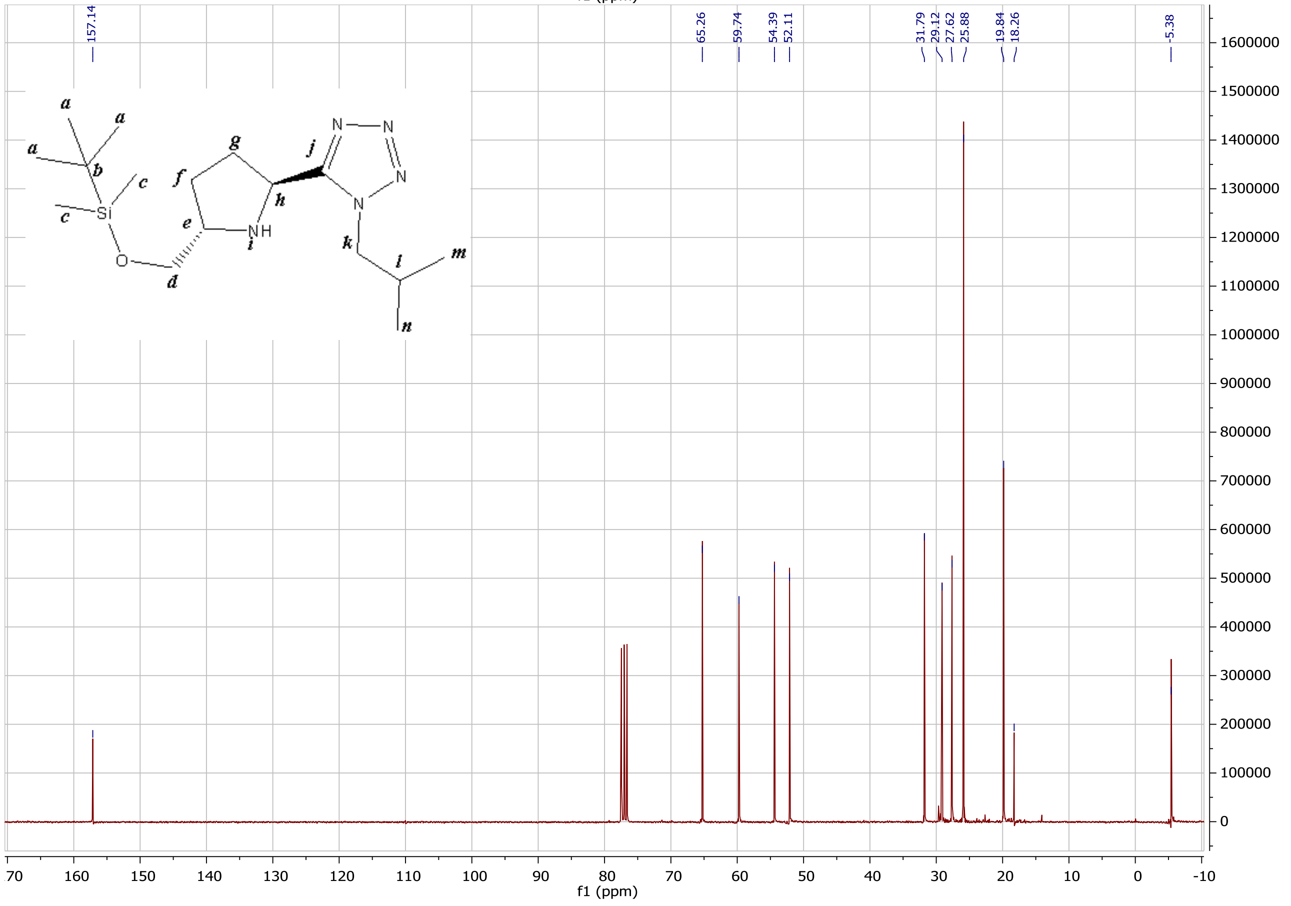

4f – *cis* isomer

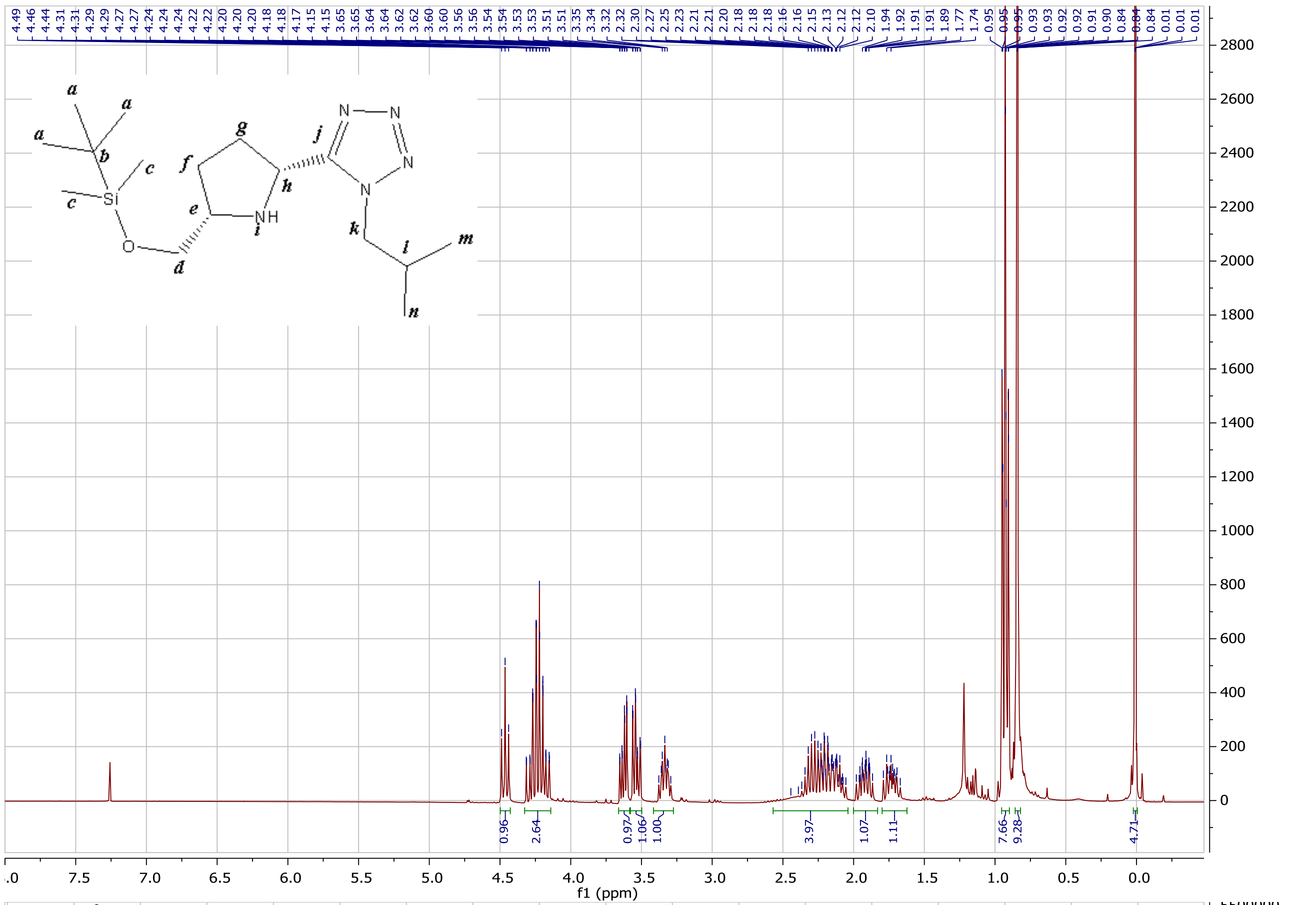

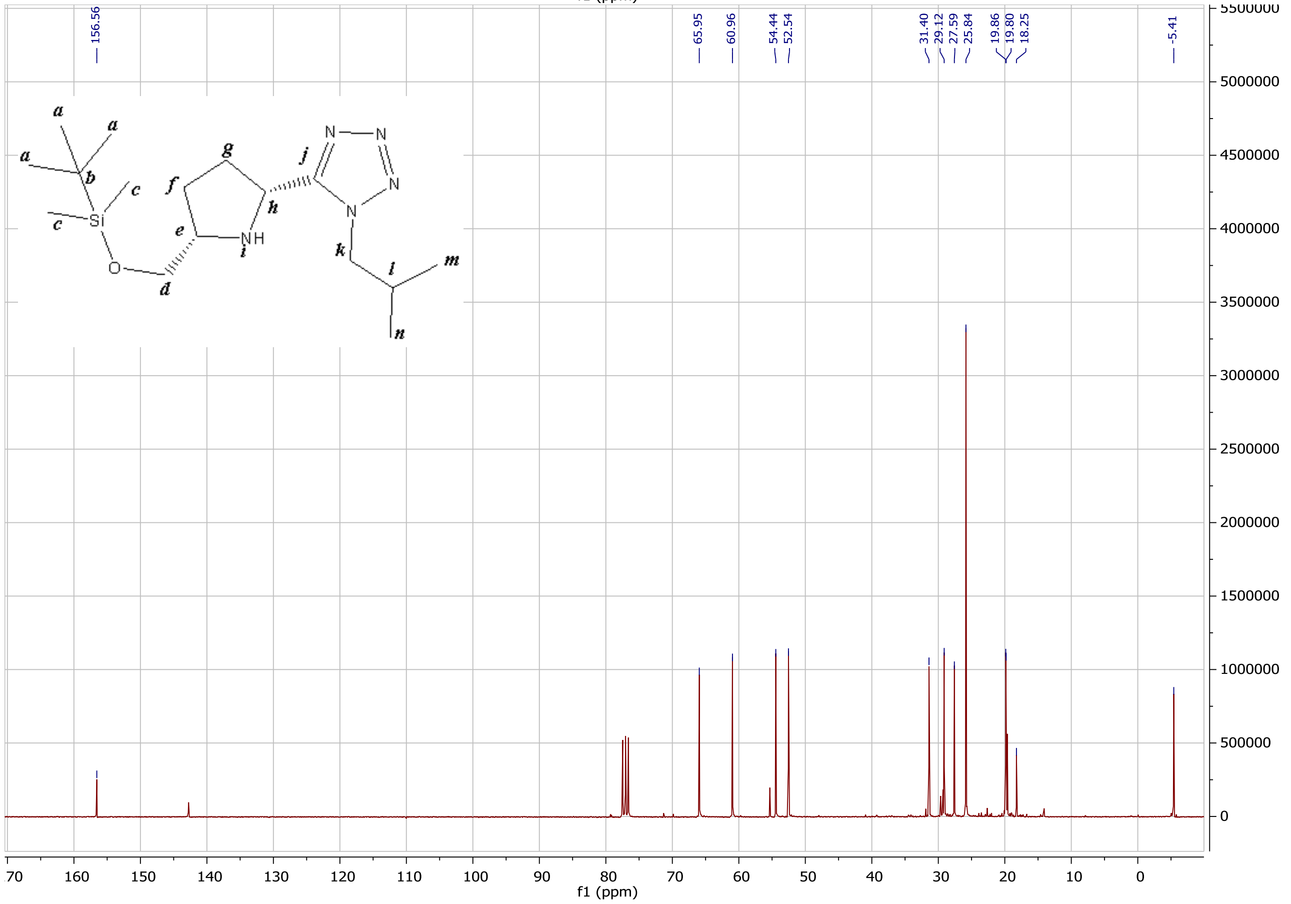

4f – NOESY 2D of *trans* and *cis* isomers

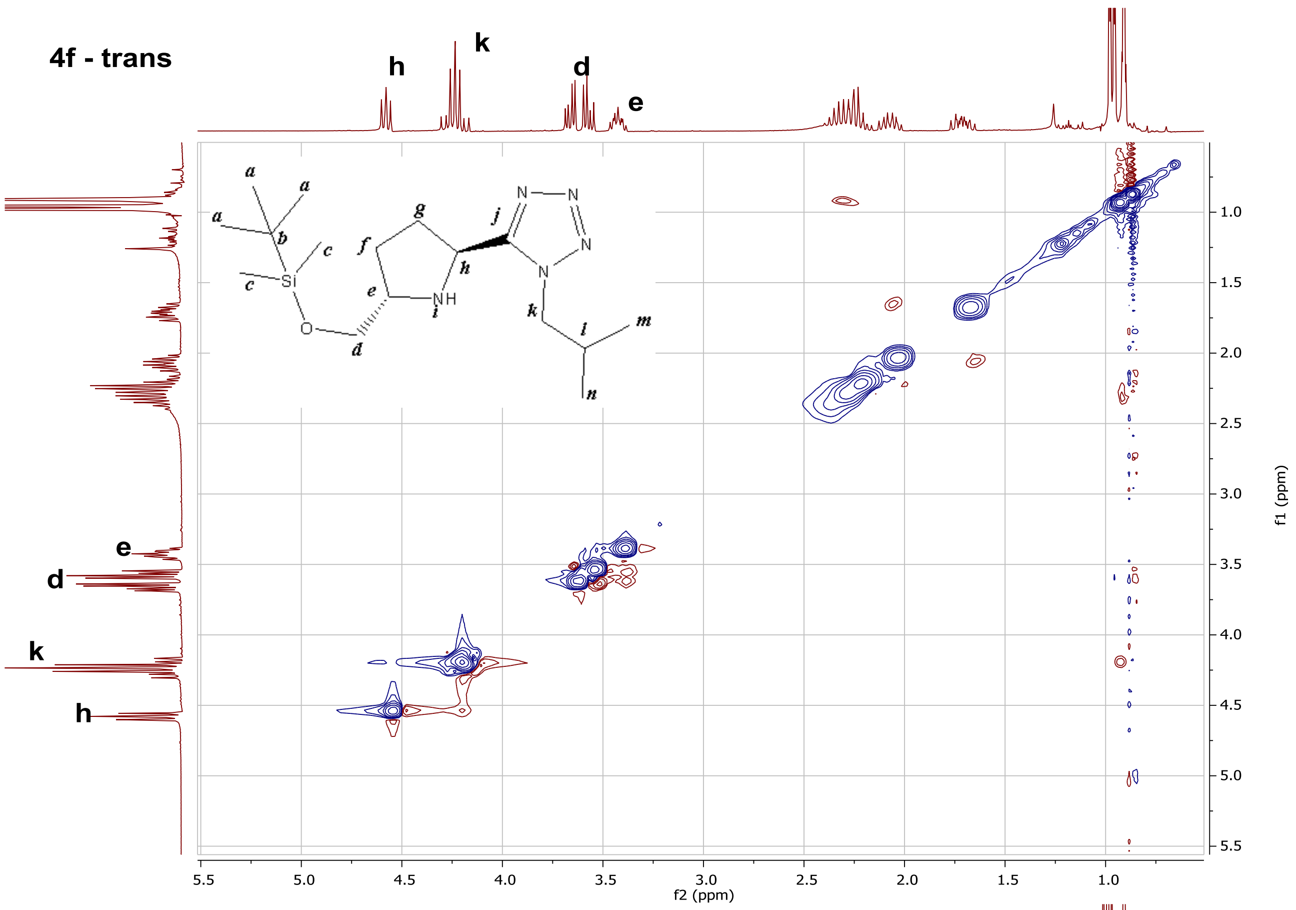

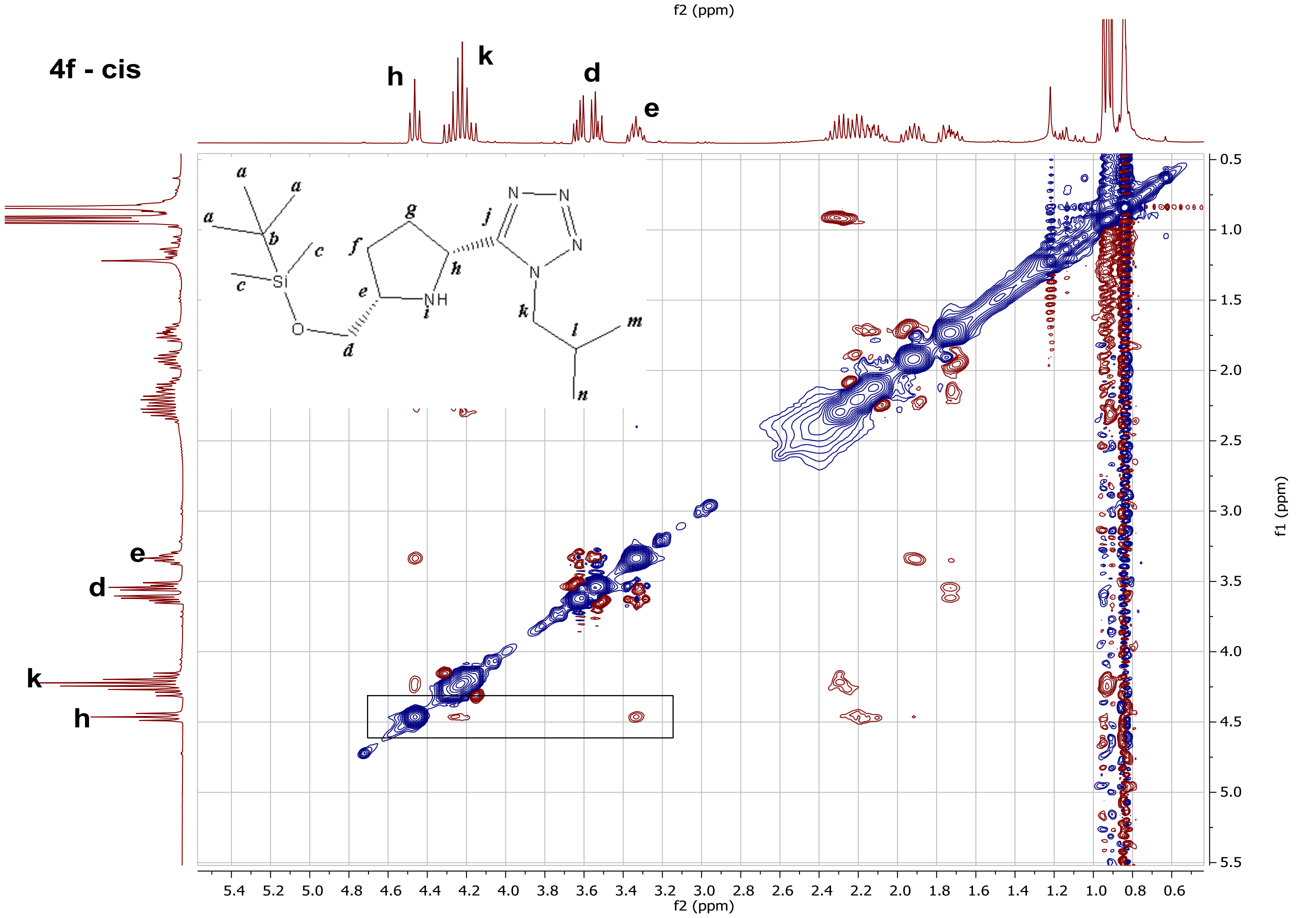

4g – *trans* isomer

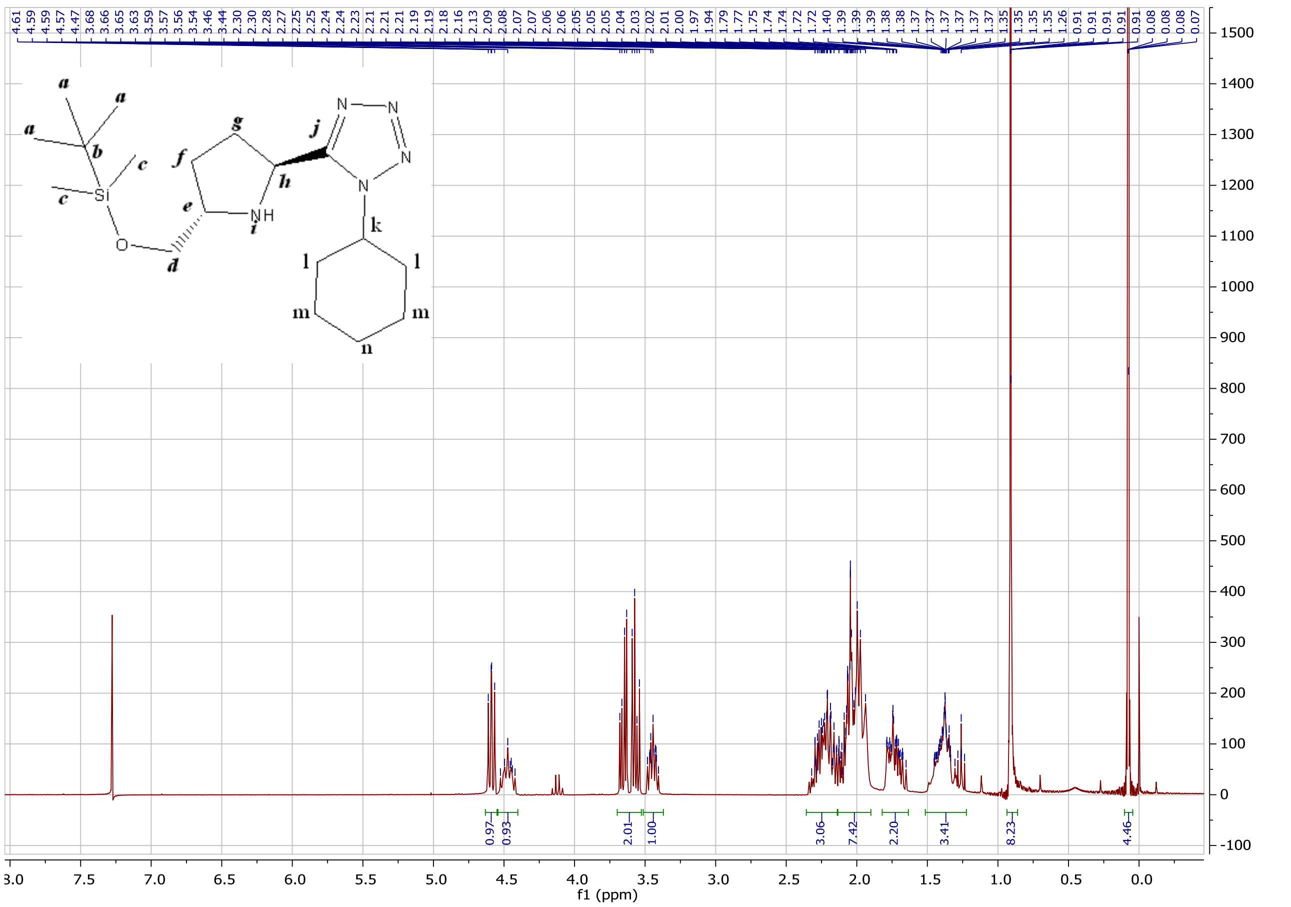

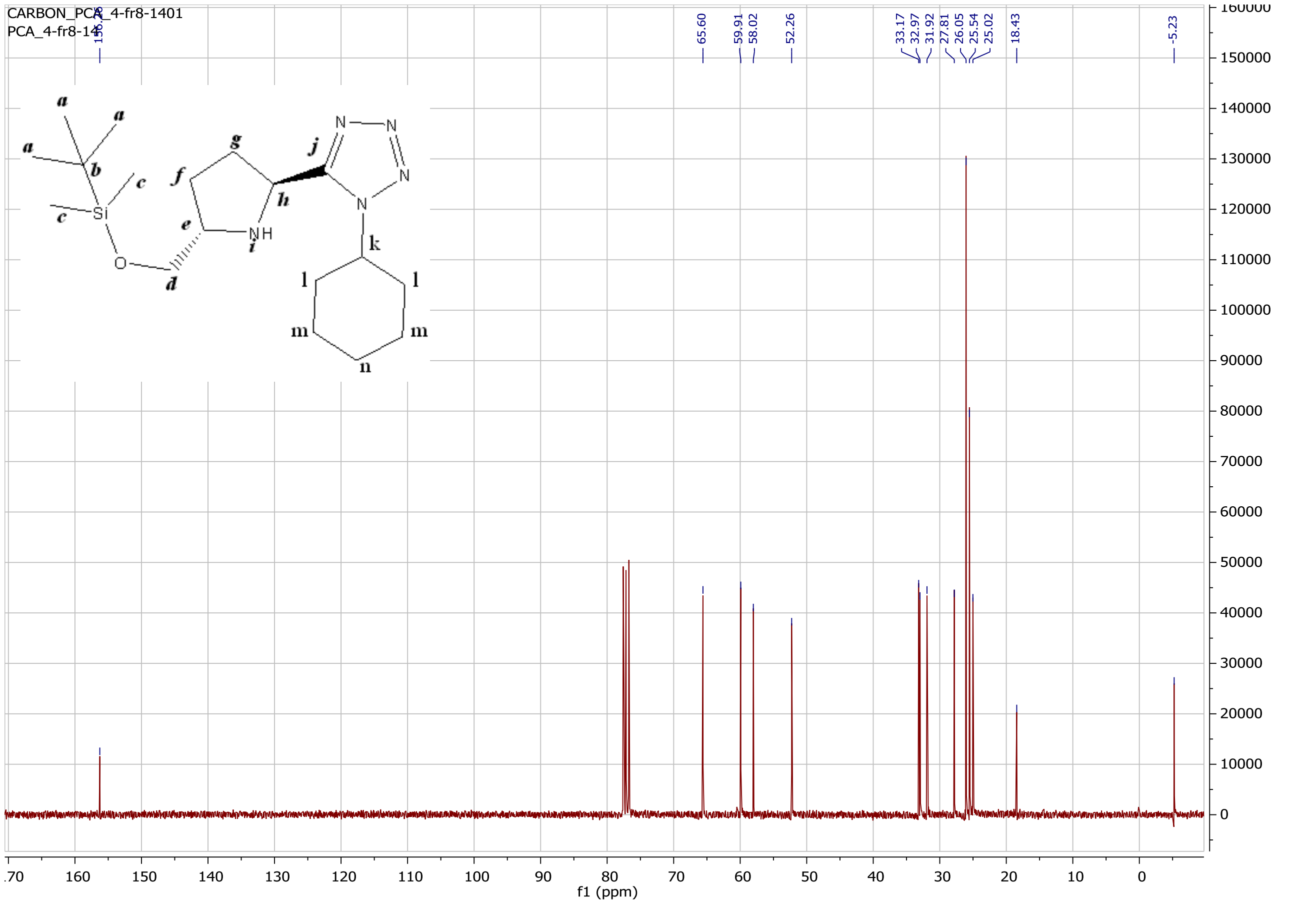

4g – *cis* isomer

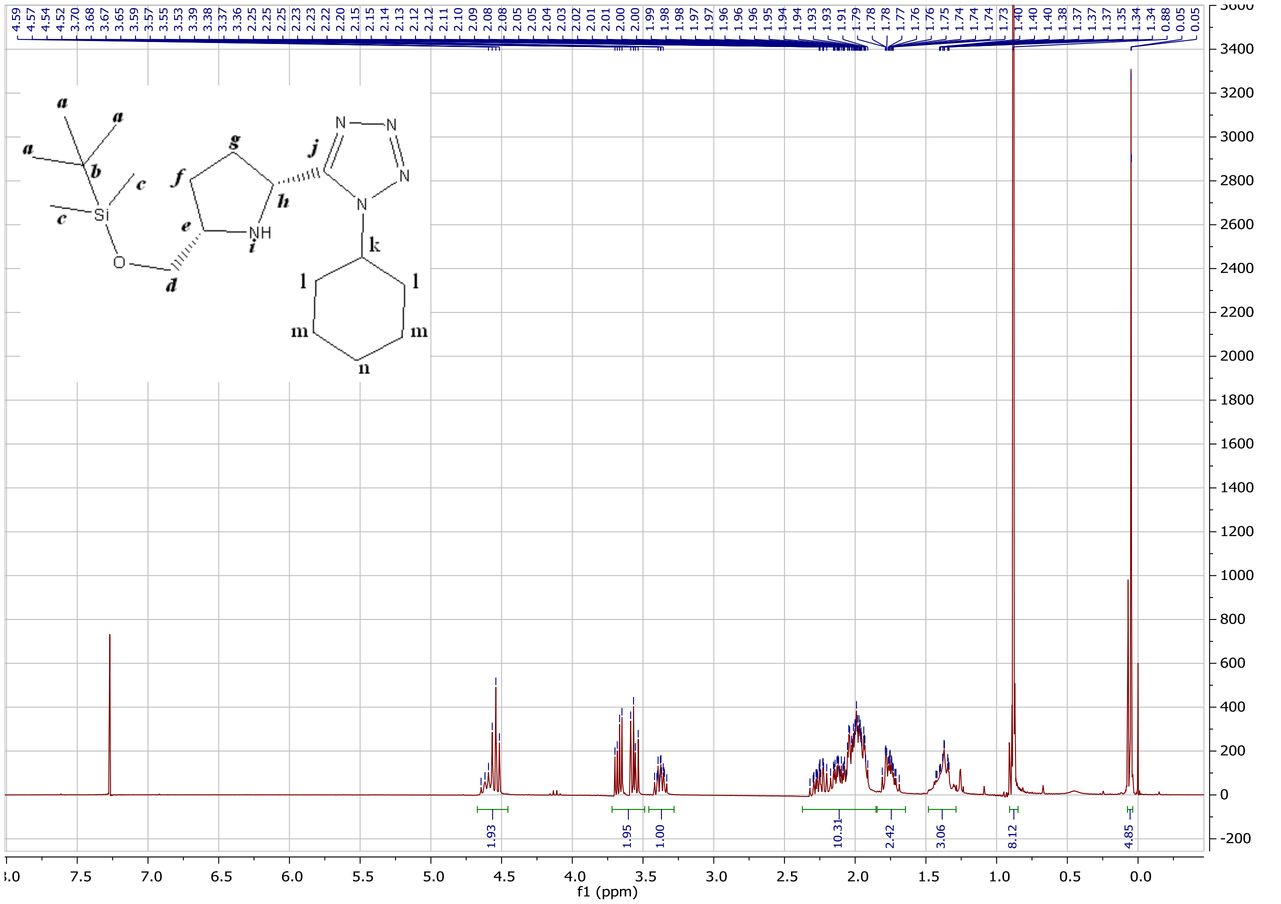

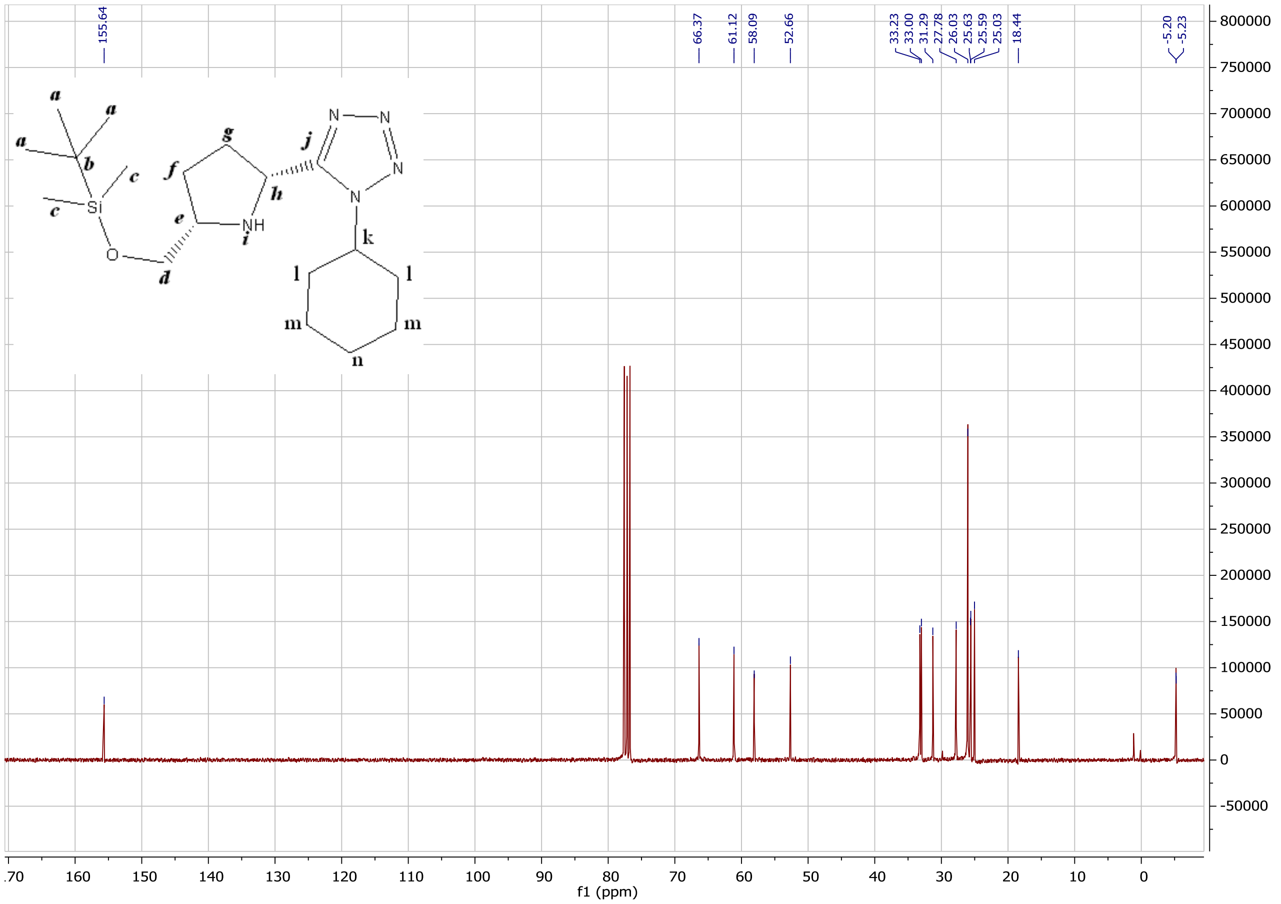

4g – NOESY 1D of *trans* and *cis* isomers

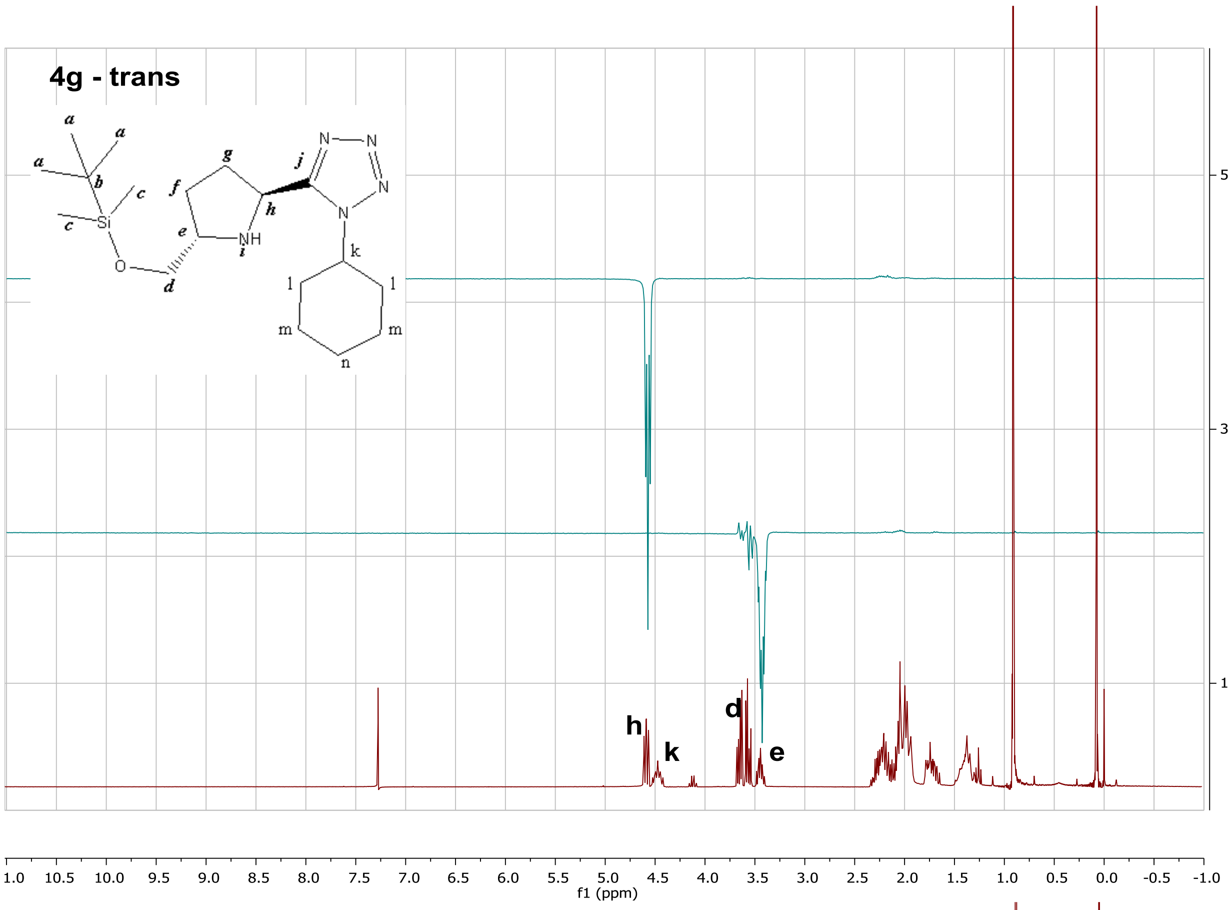

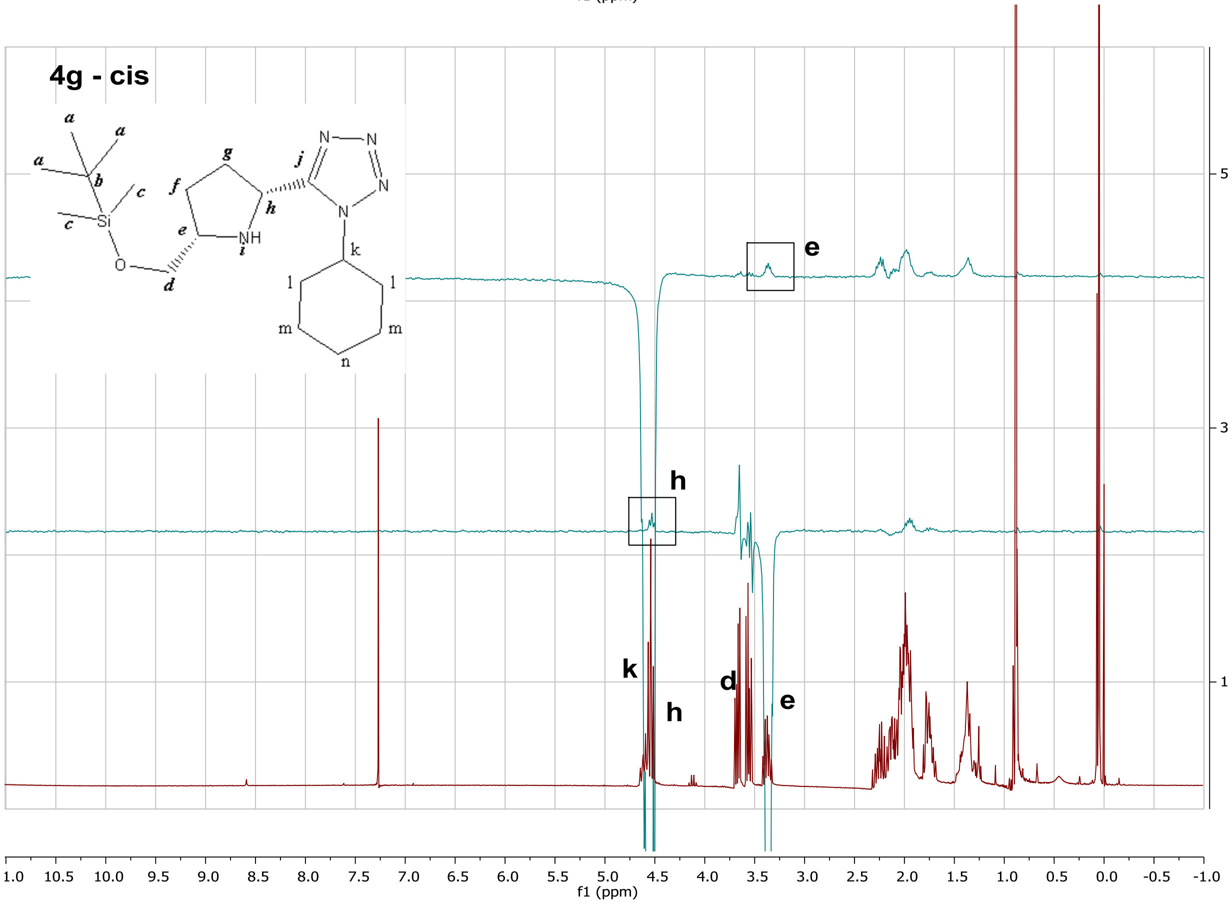

4h – *trans* isomer

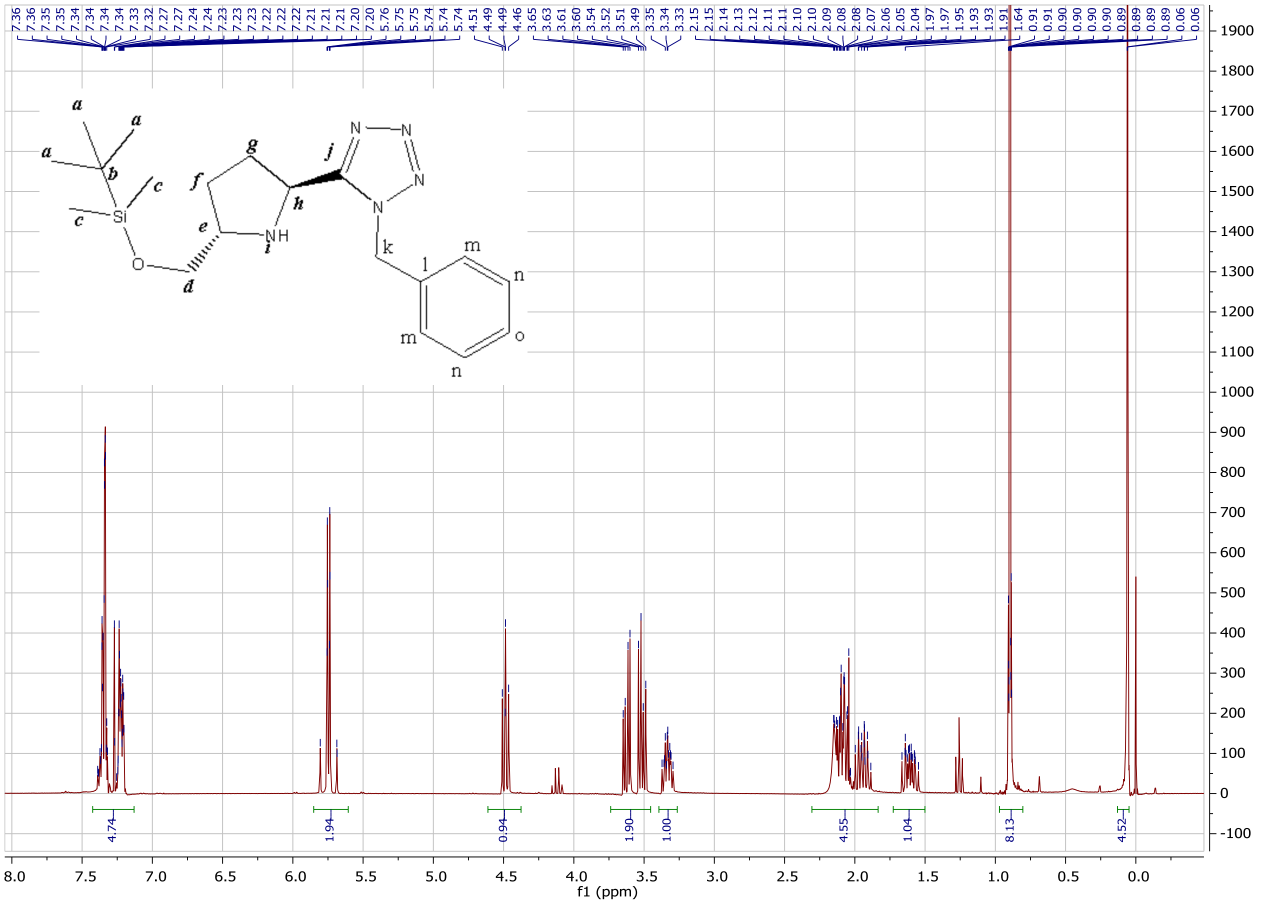

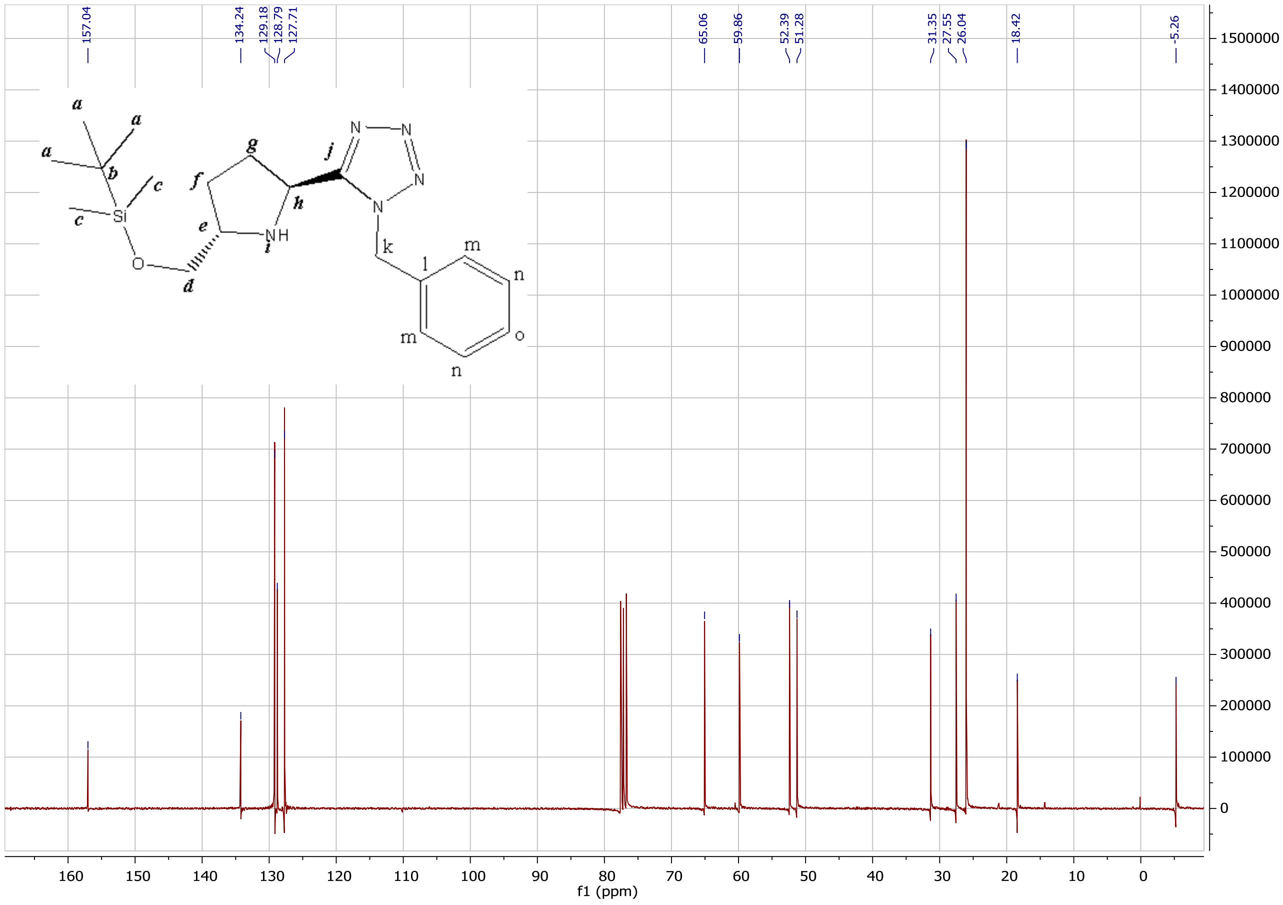

4h – *cis* isomer

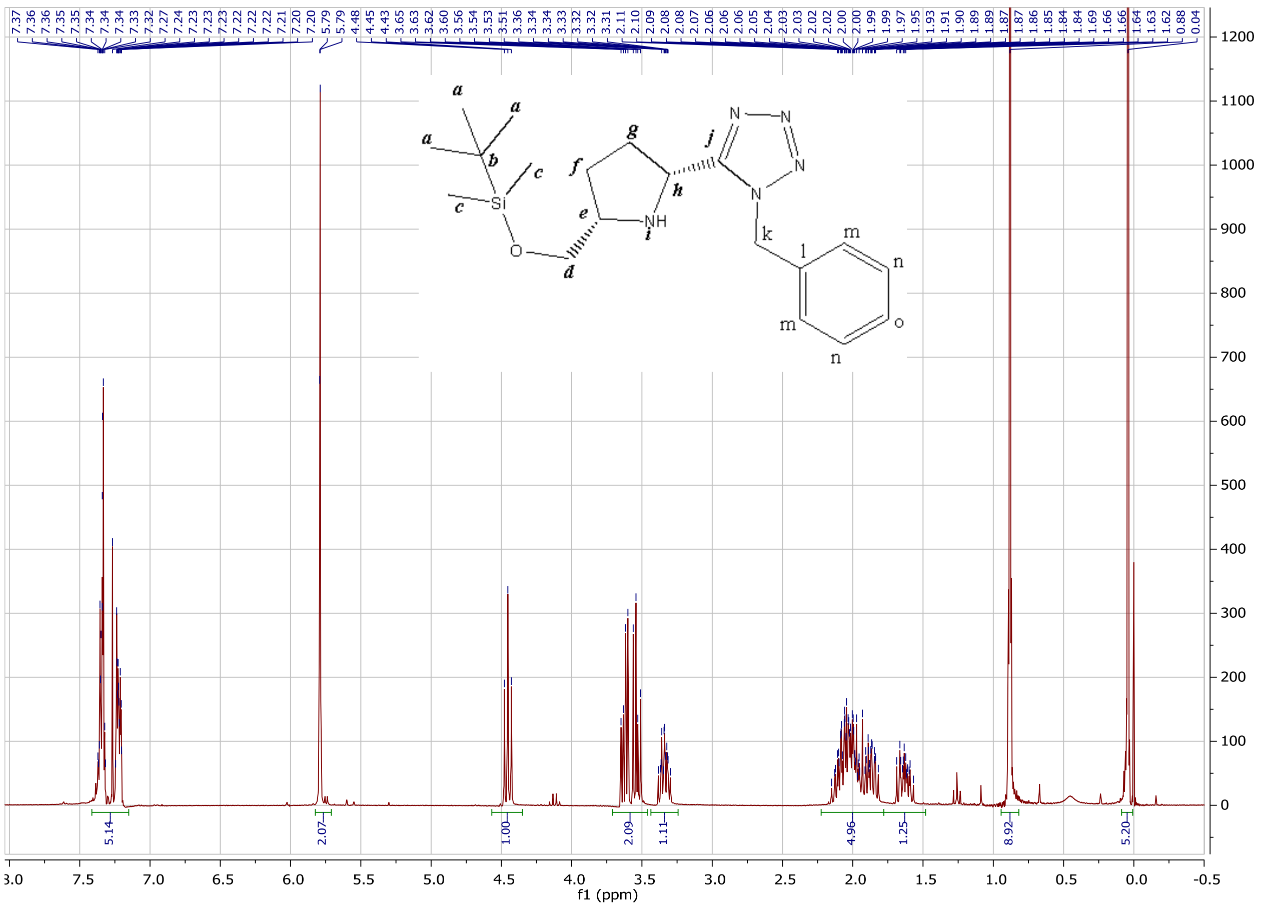

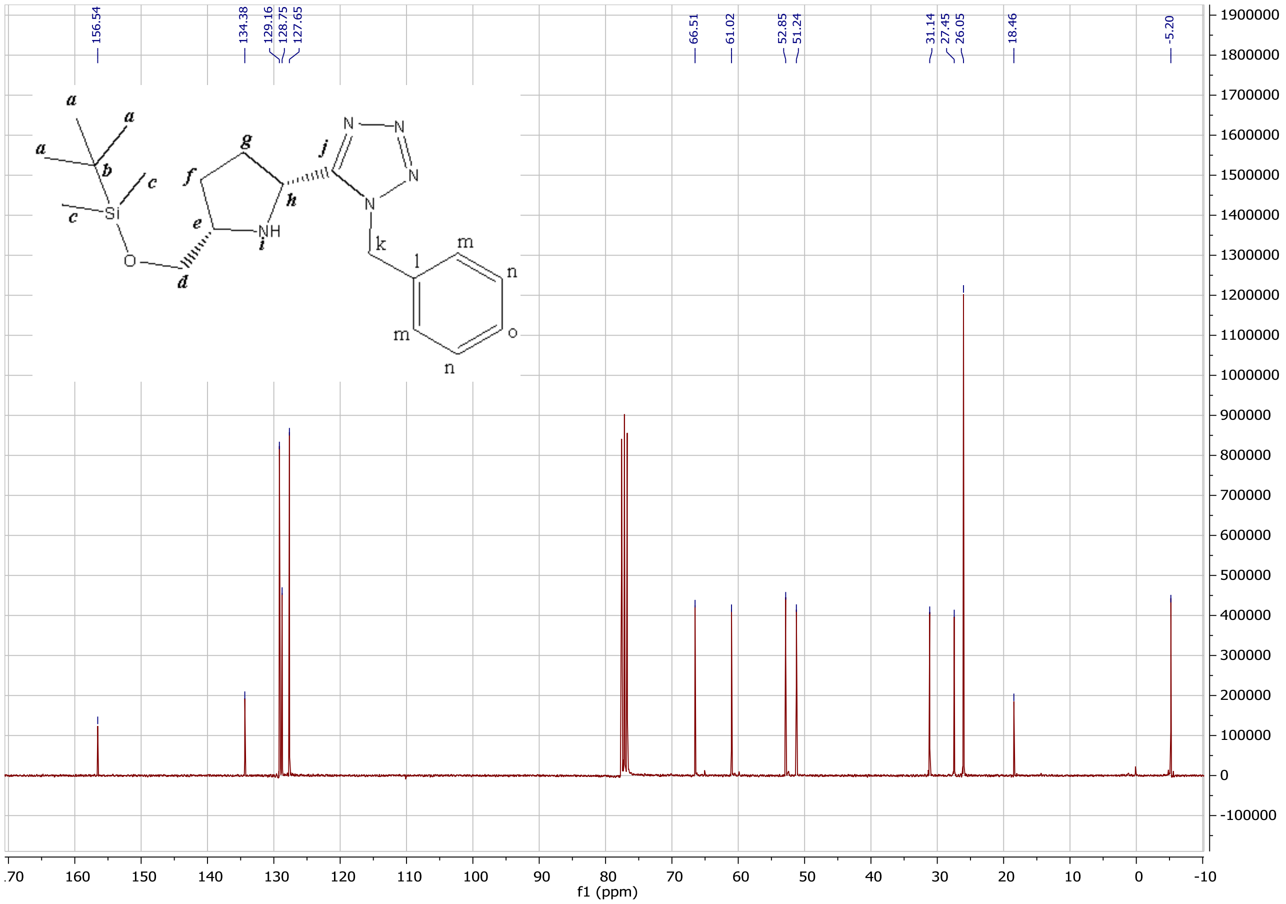

4h – NOESY 1D and 2D of *trans* and *cis* isomers

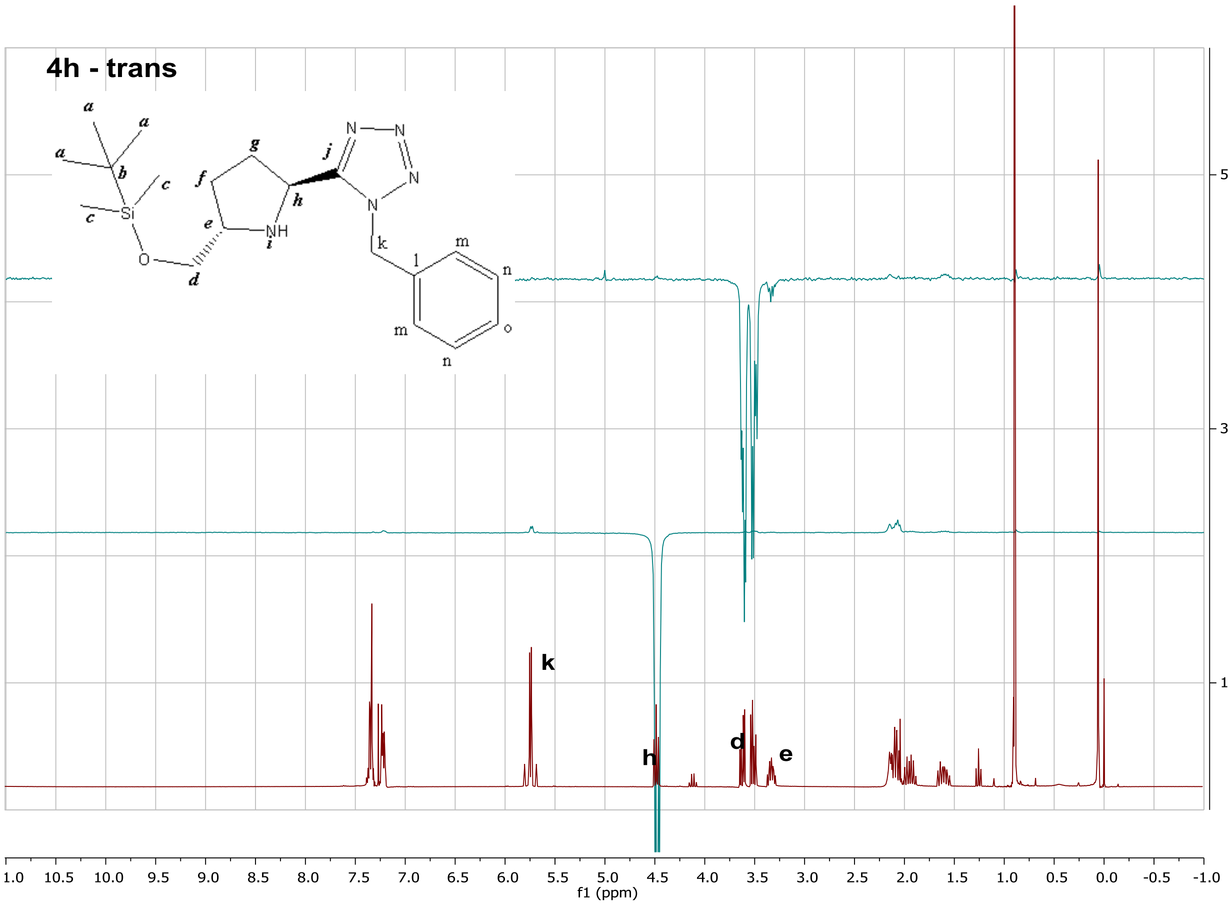

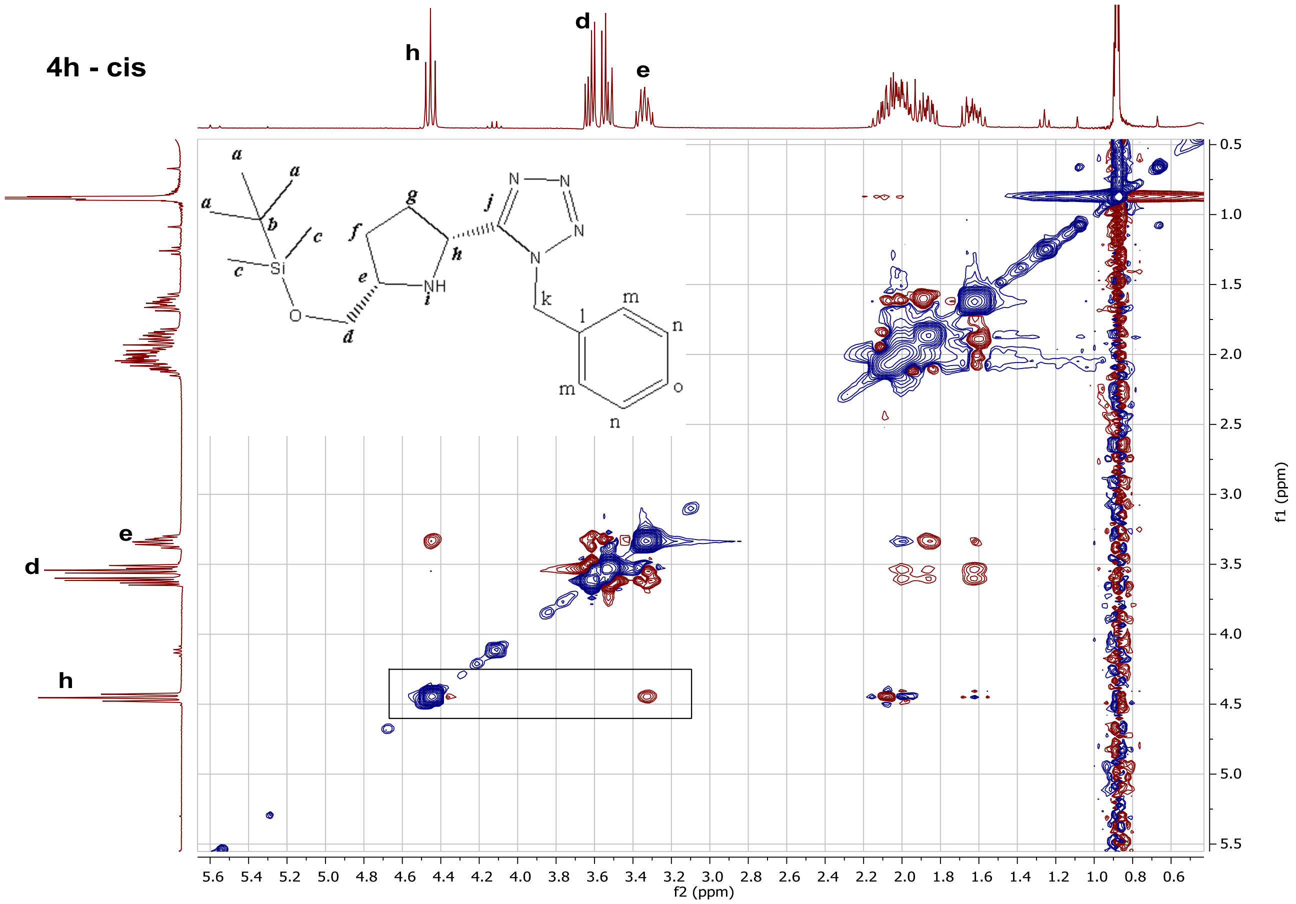

4i – *trans* isomer

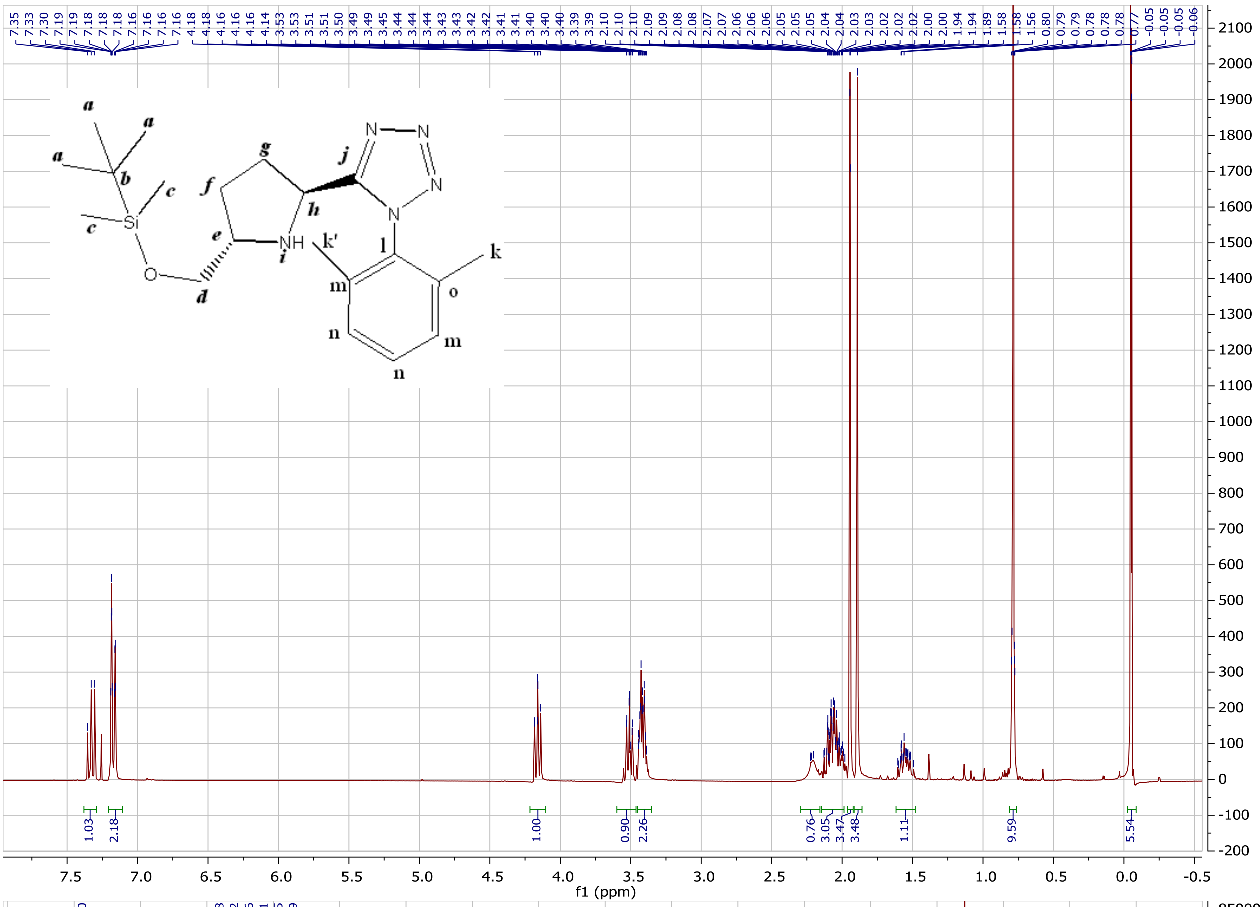

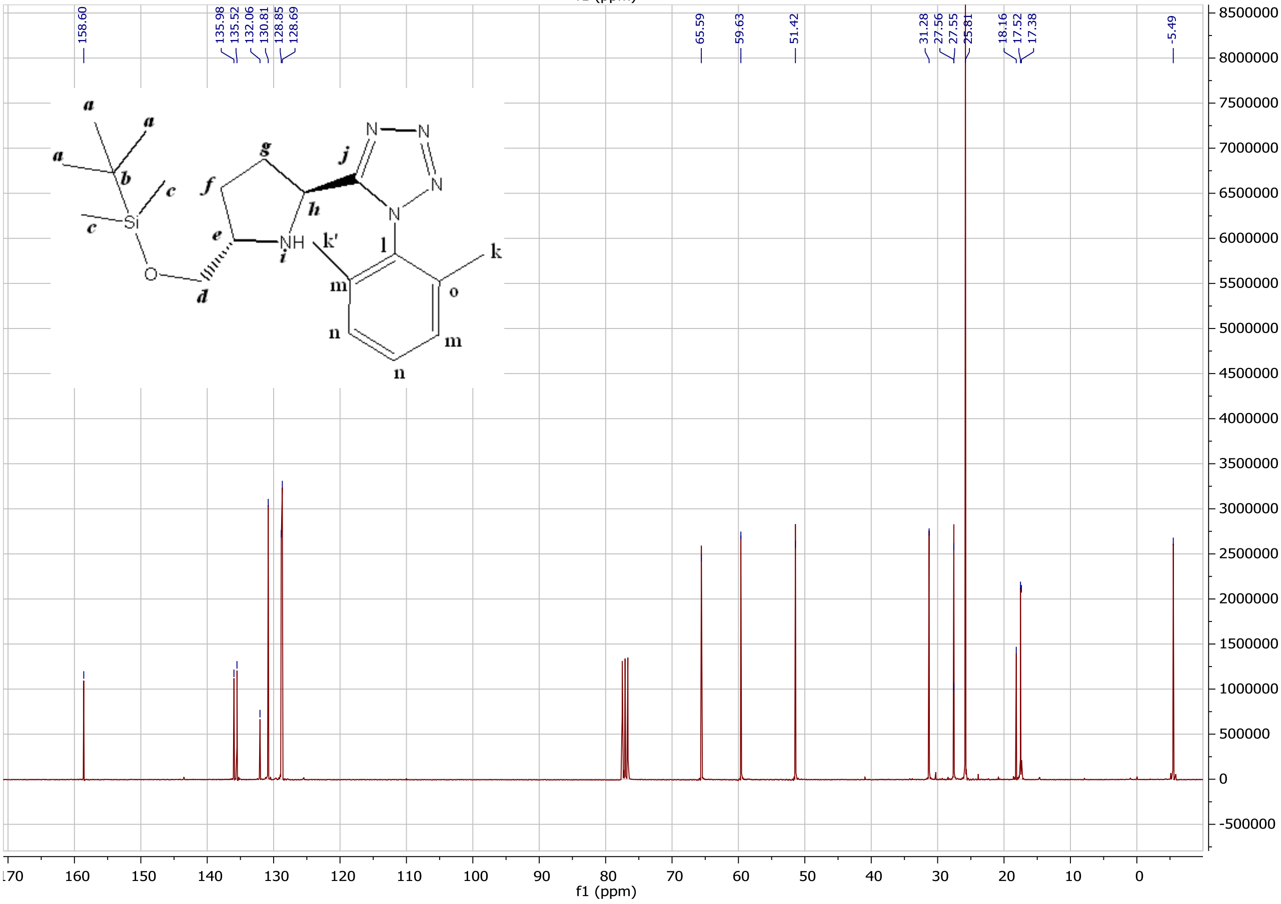

4i – *cis* isomer

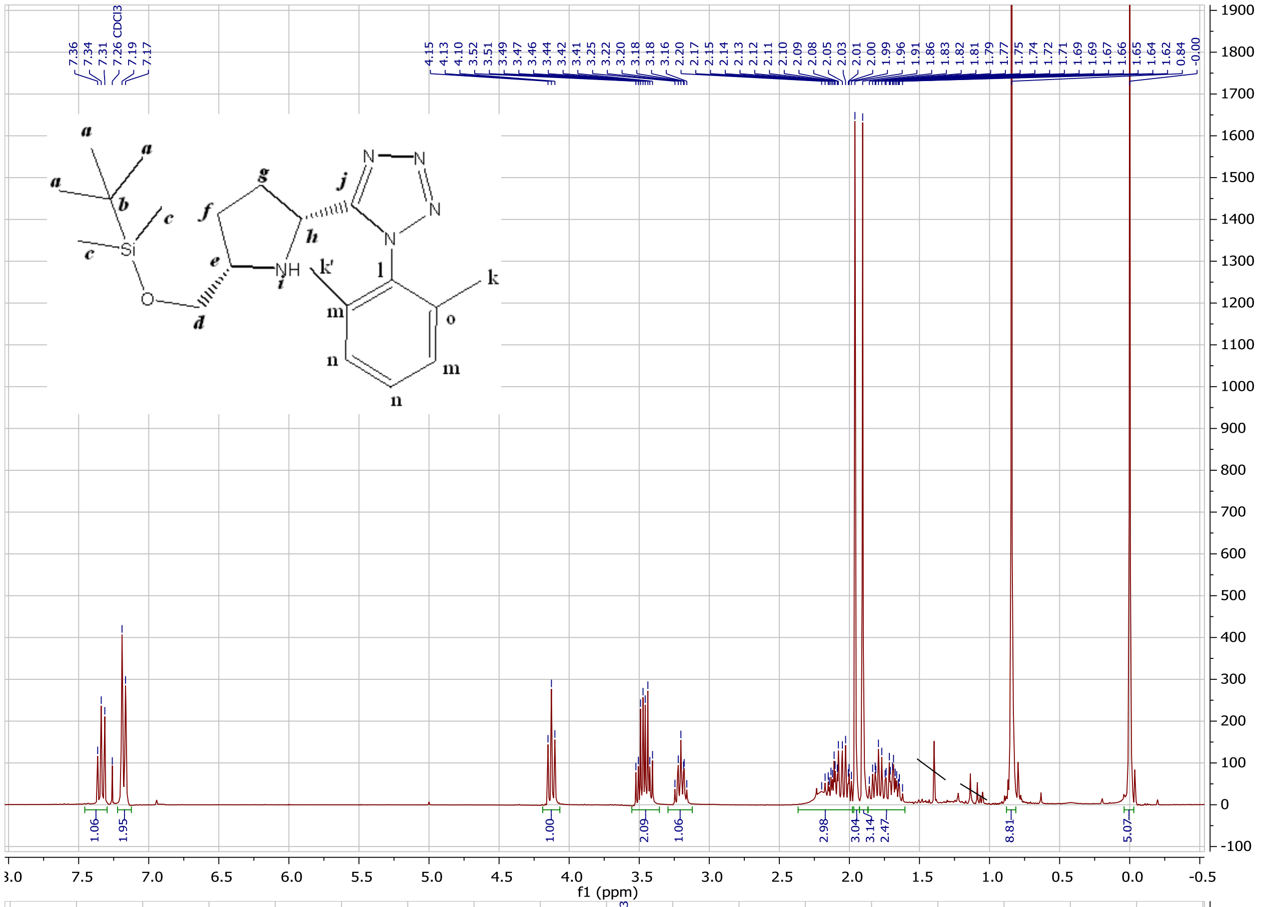

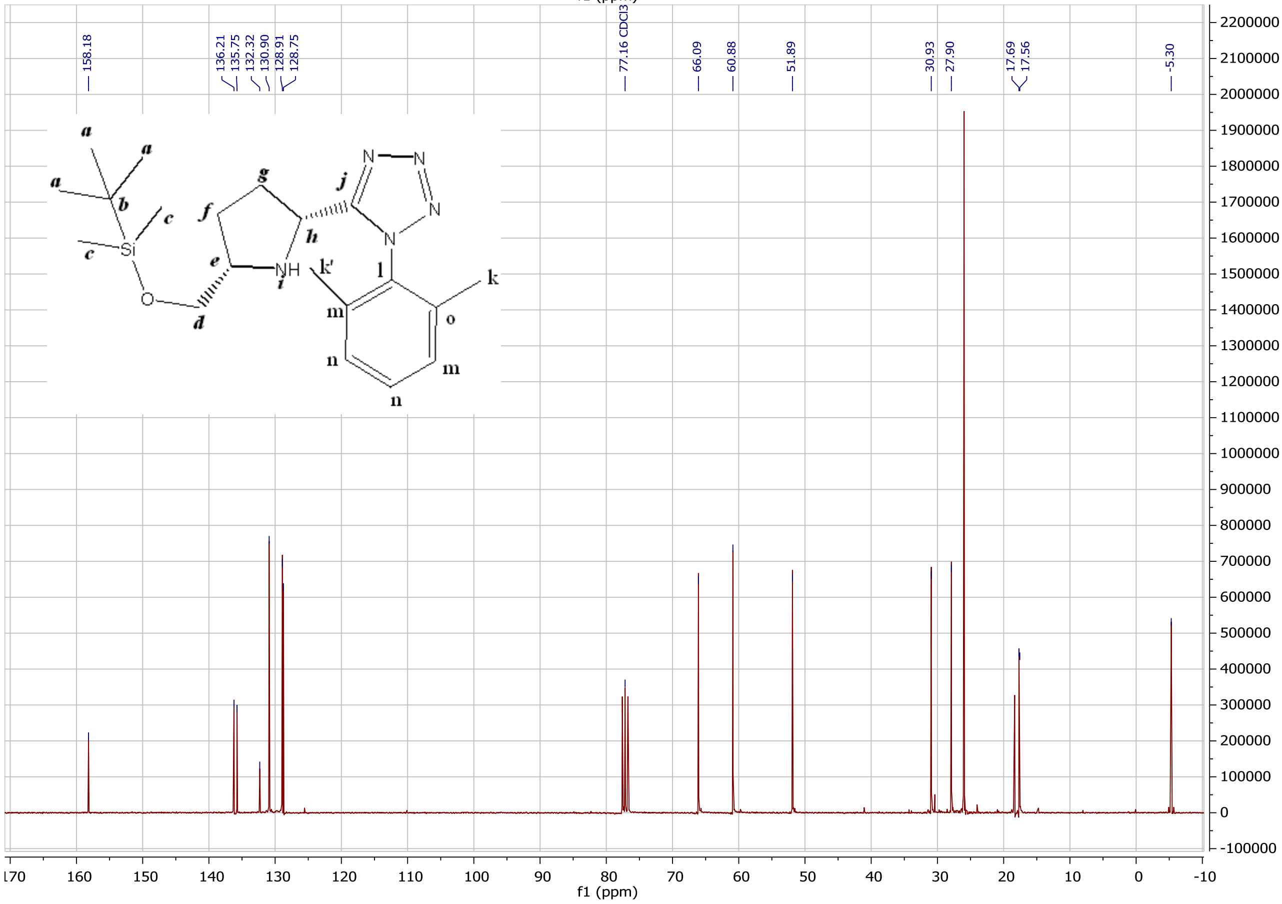

4i – NOESY 2D of *cis* isomer

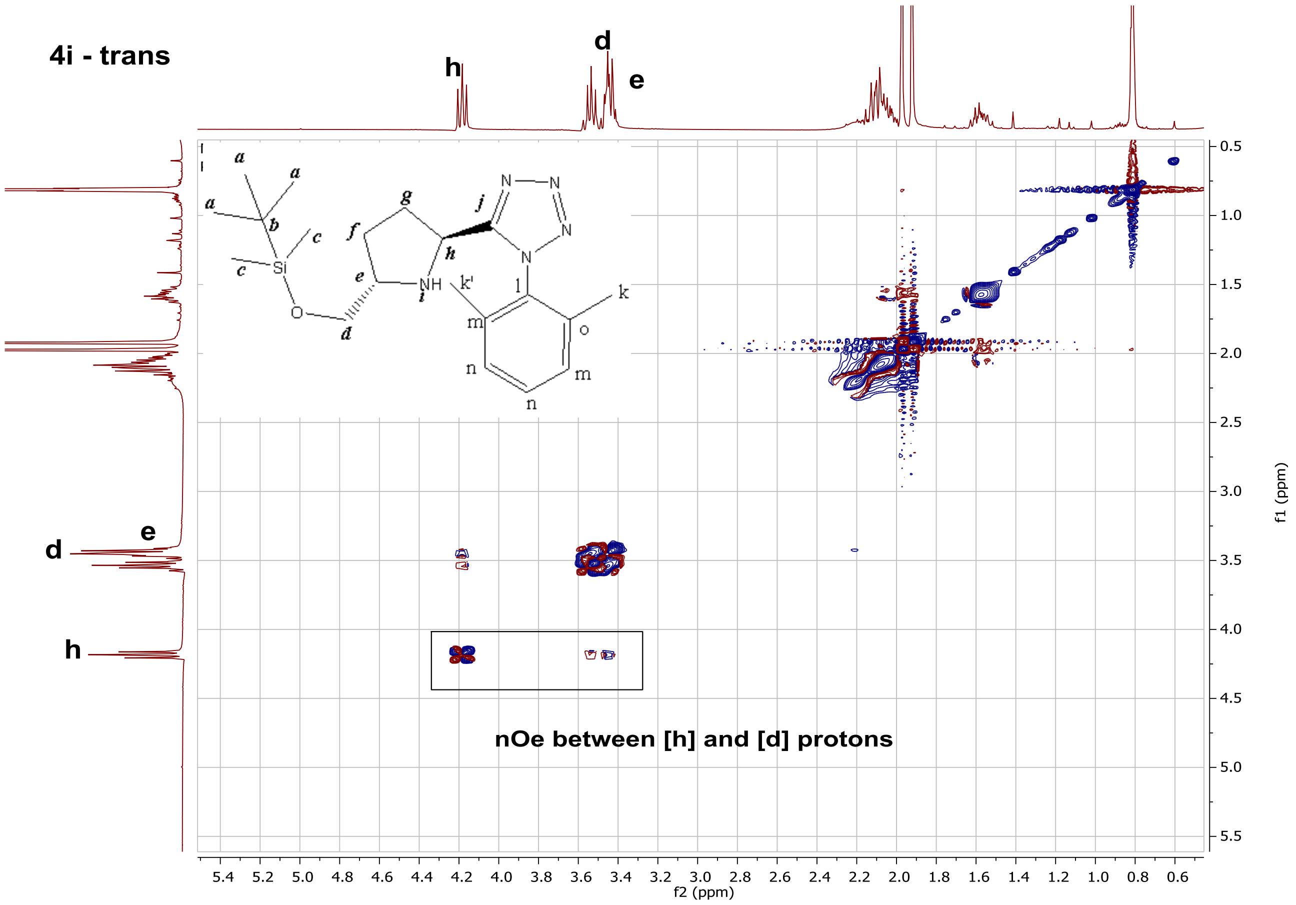

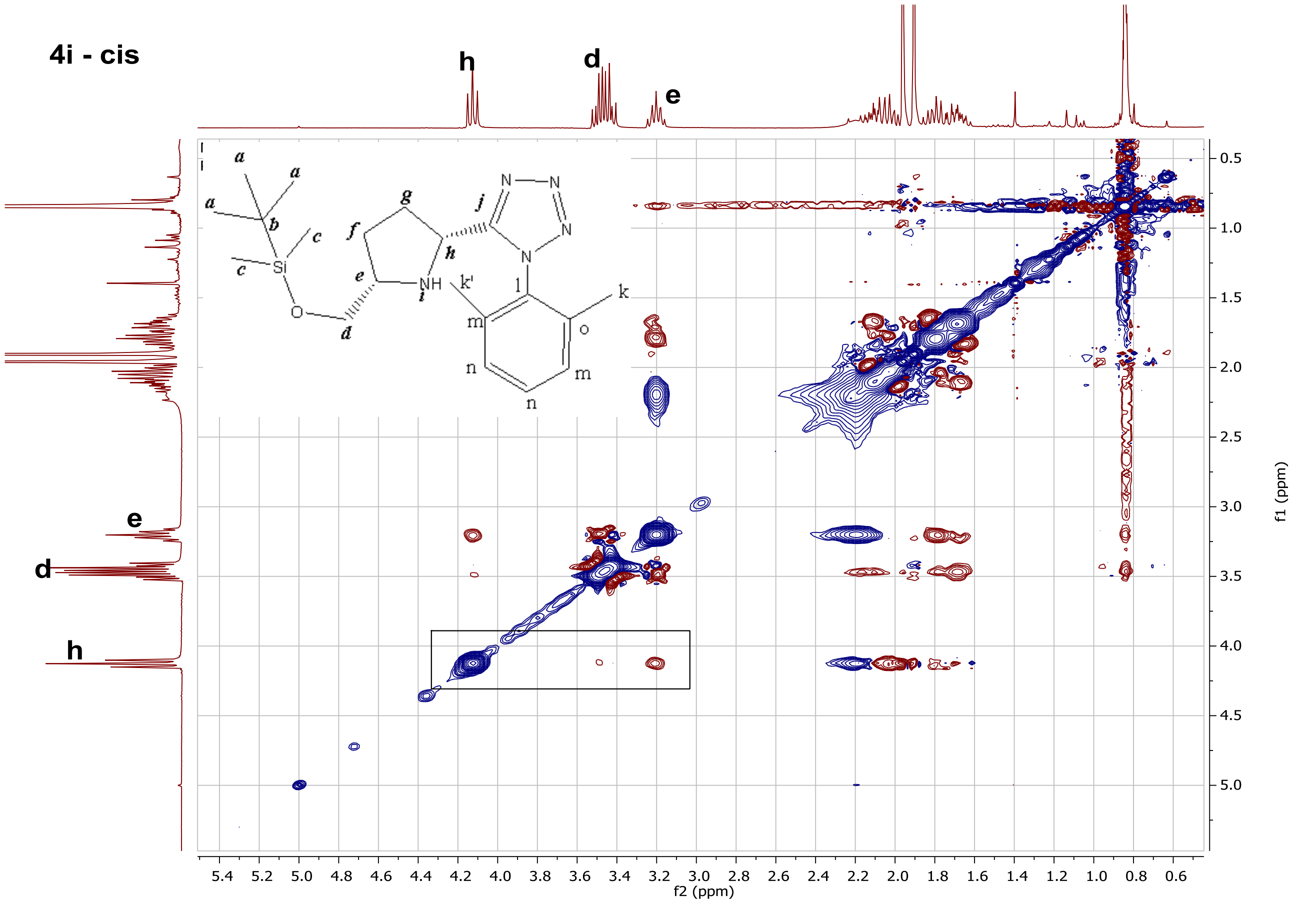

4j – *trans* isomer

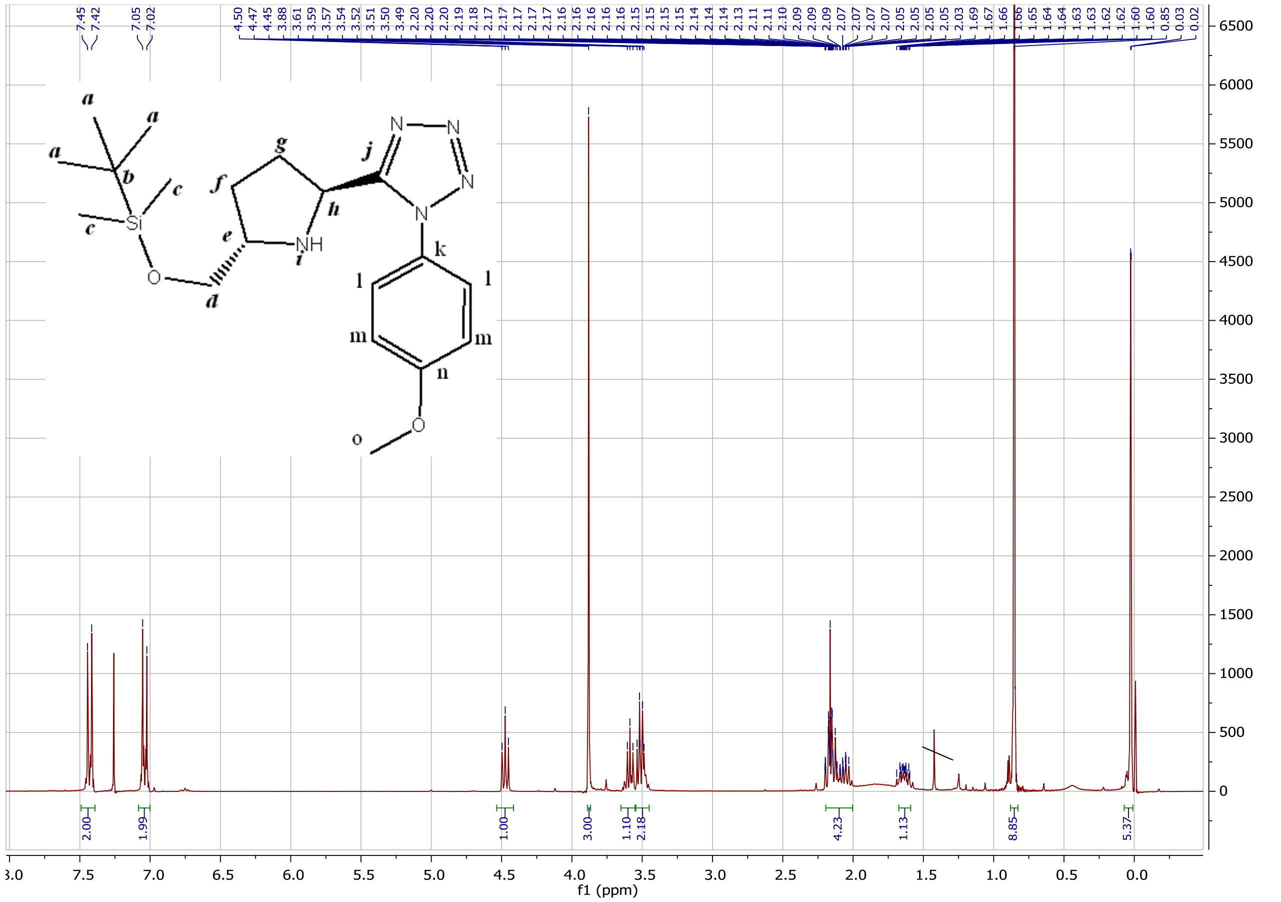

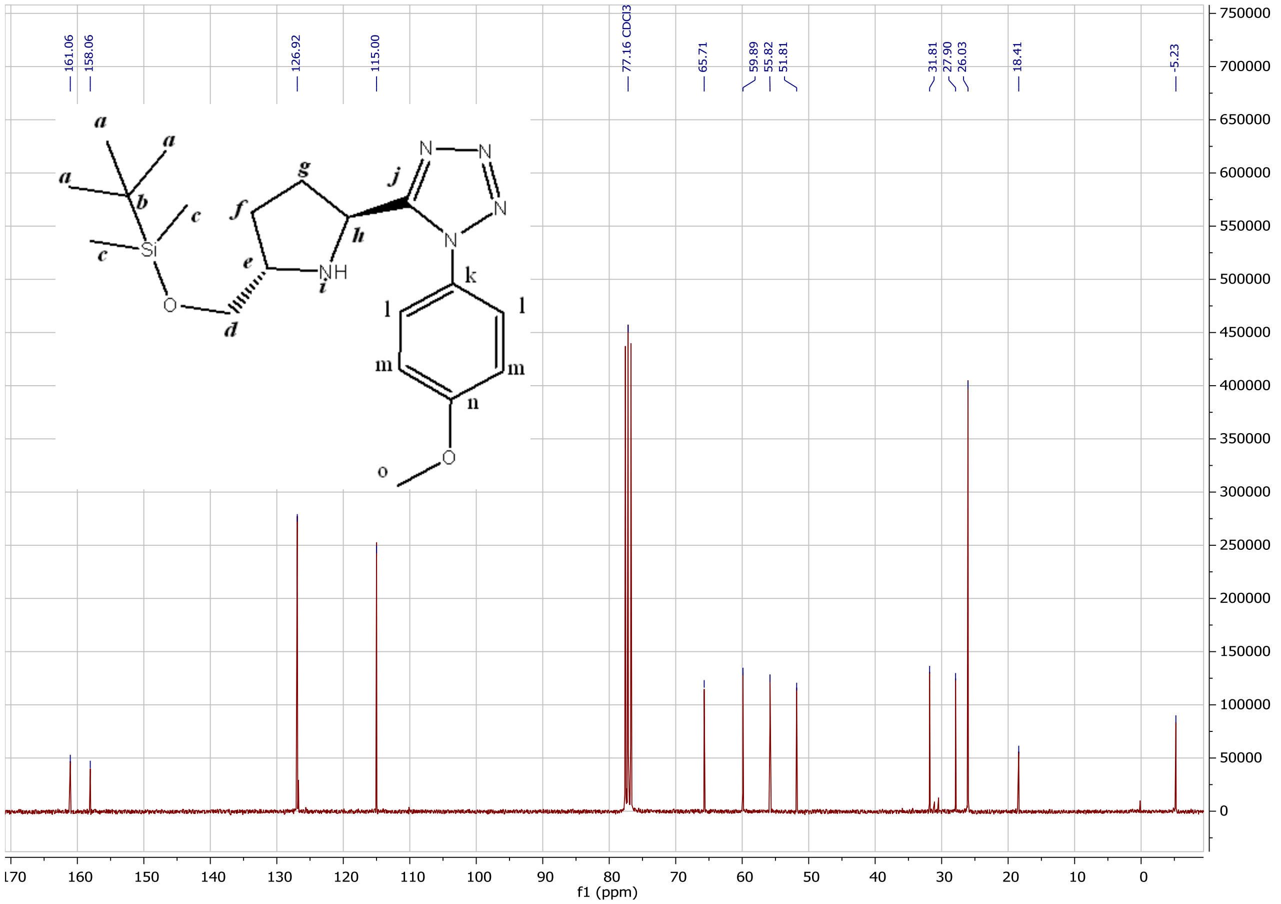

4j – *cis* isomer

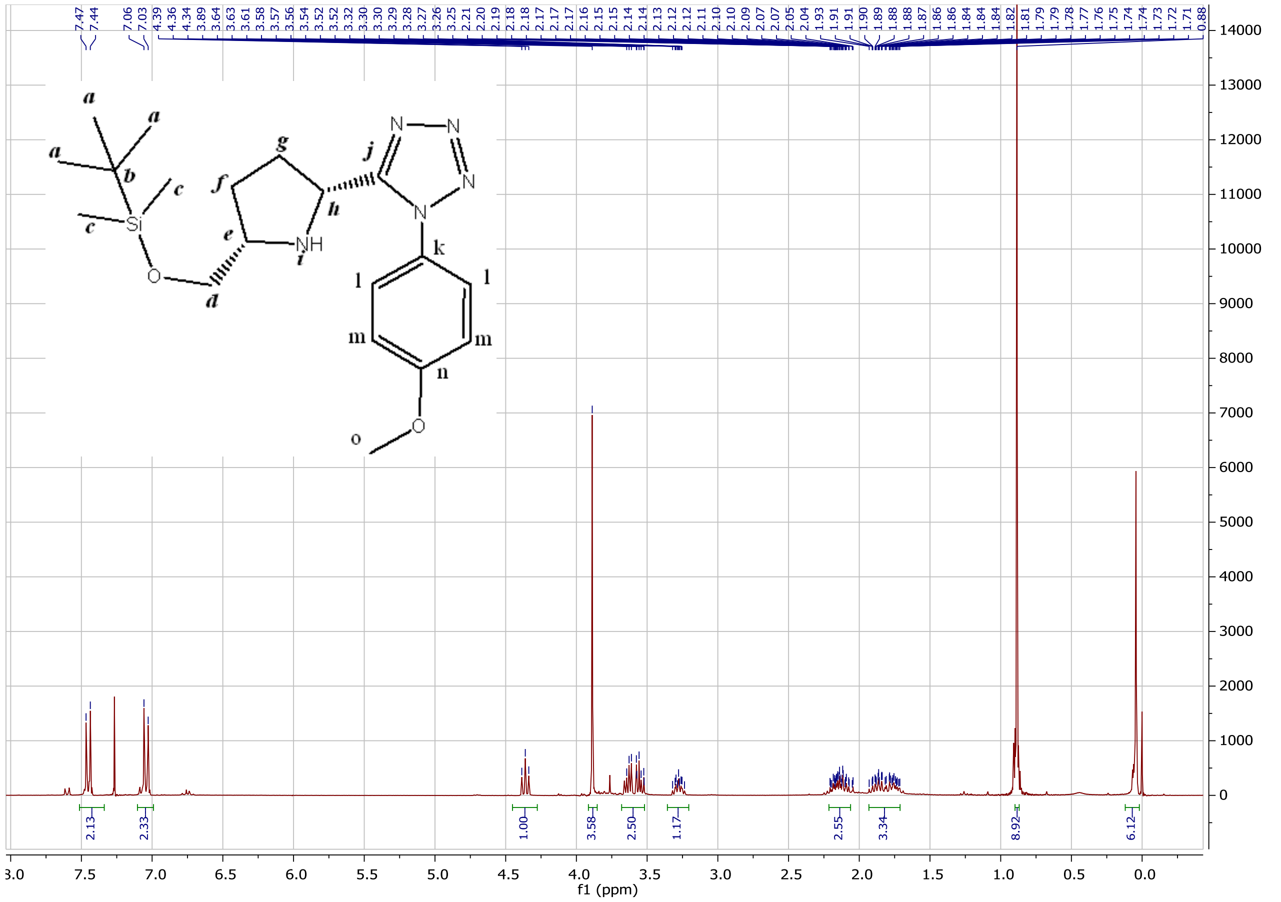

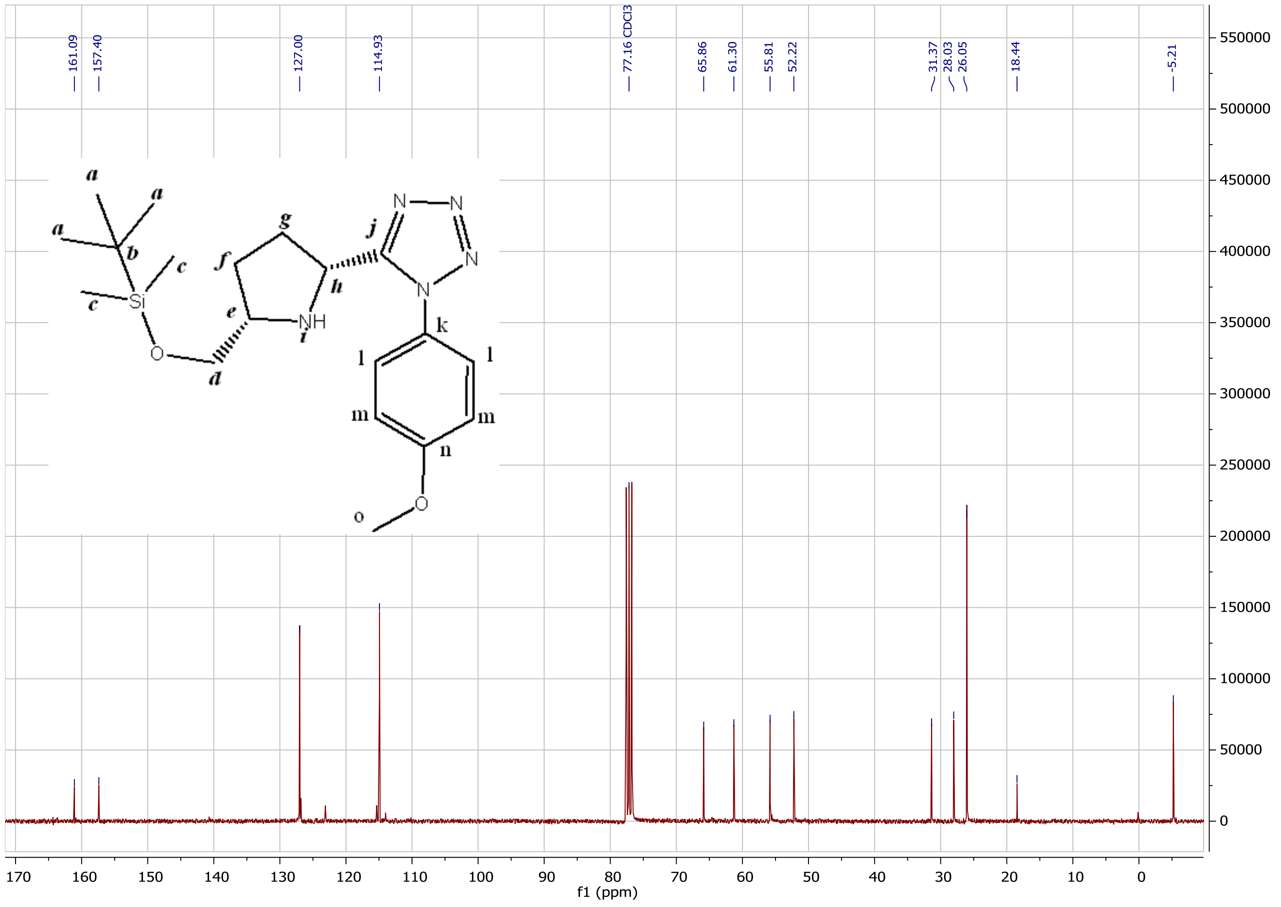

4j – NOESY 2D of *trans* and *cis* isomers

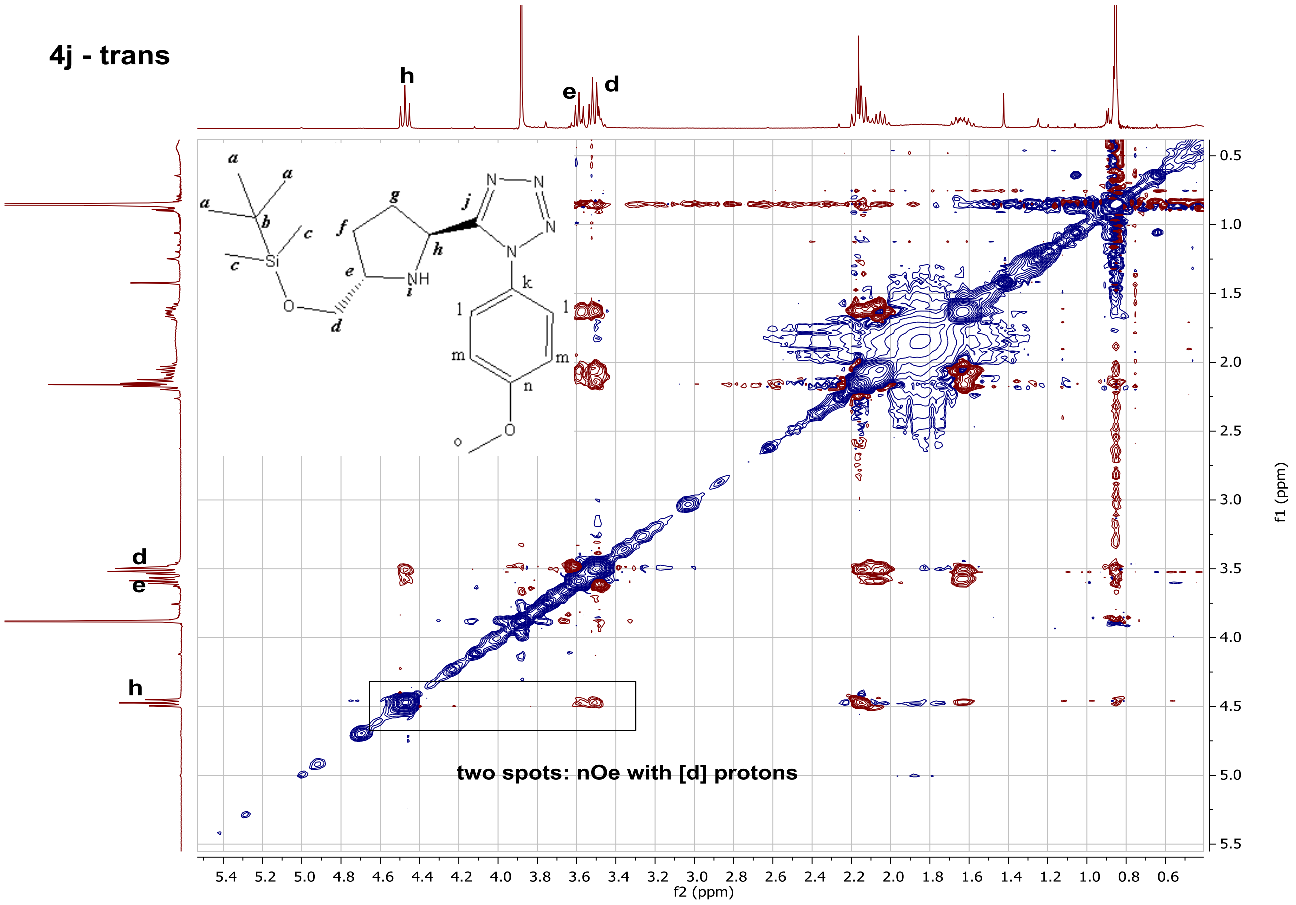

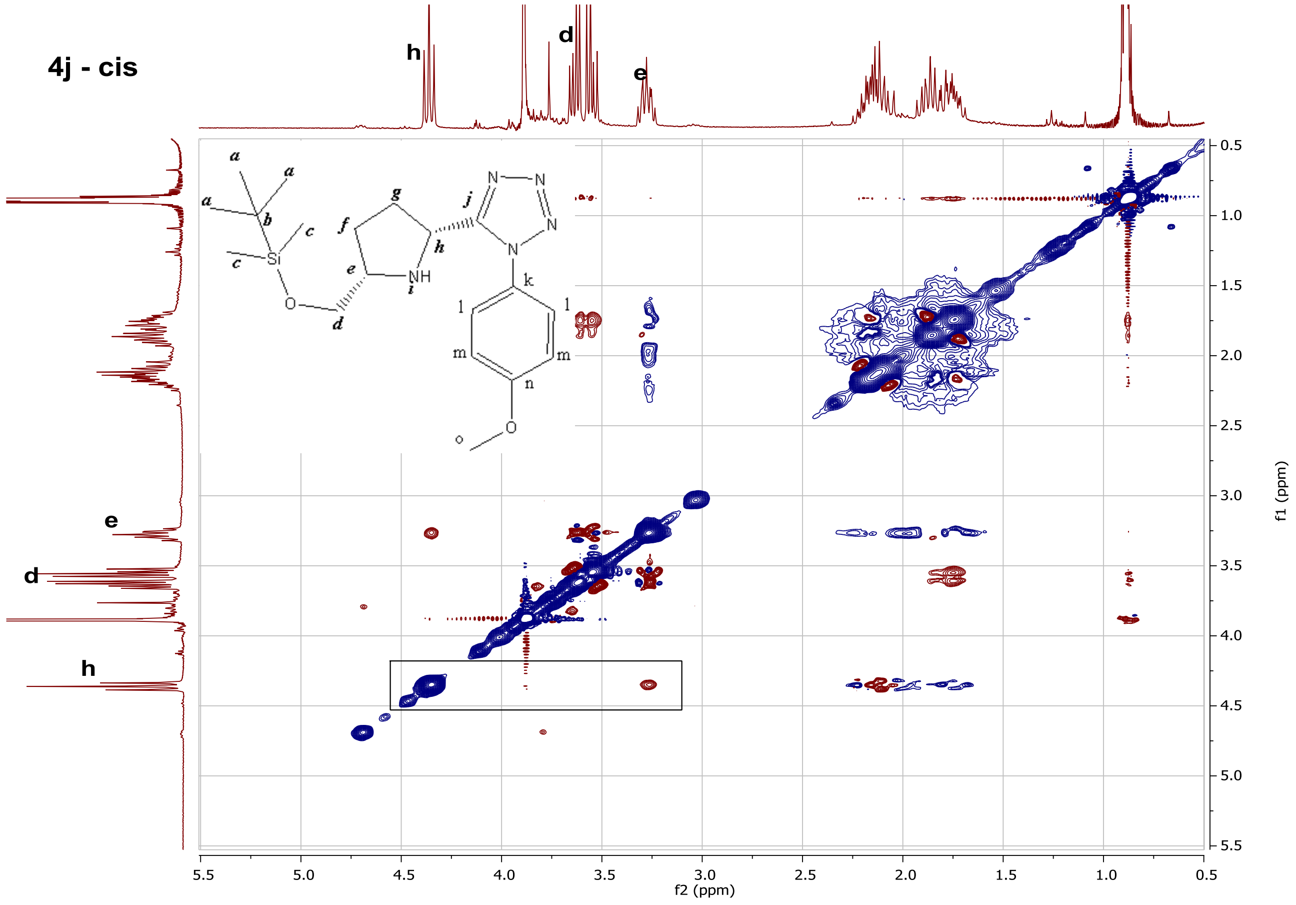

4k – *trans* isomer

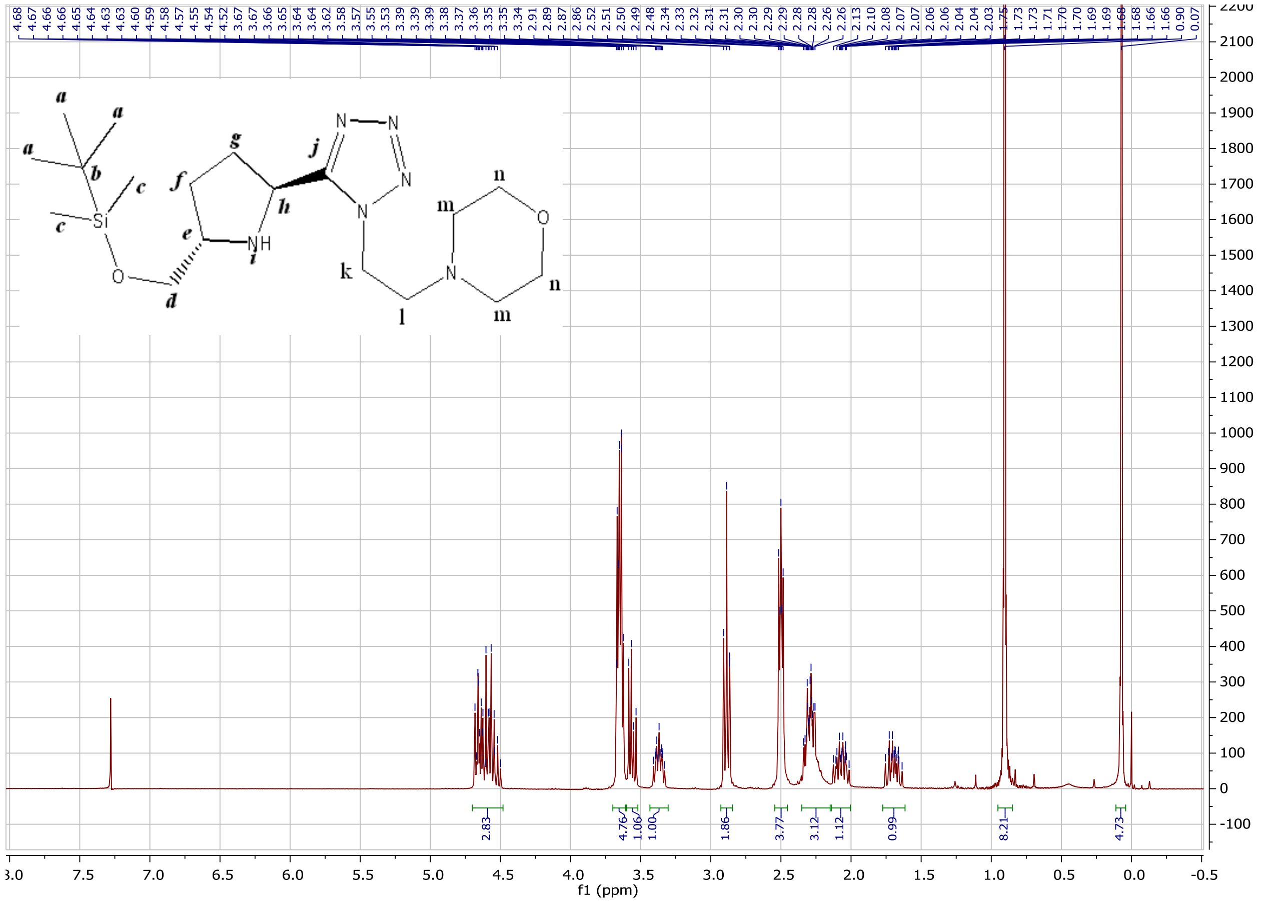

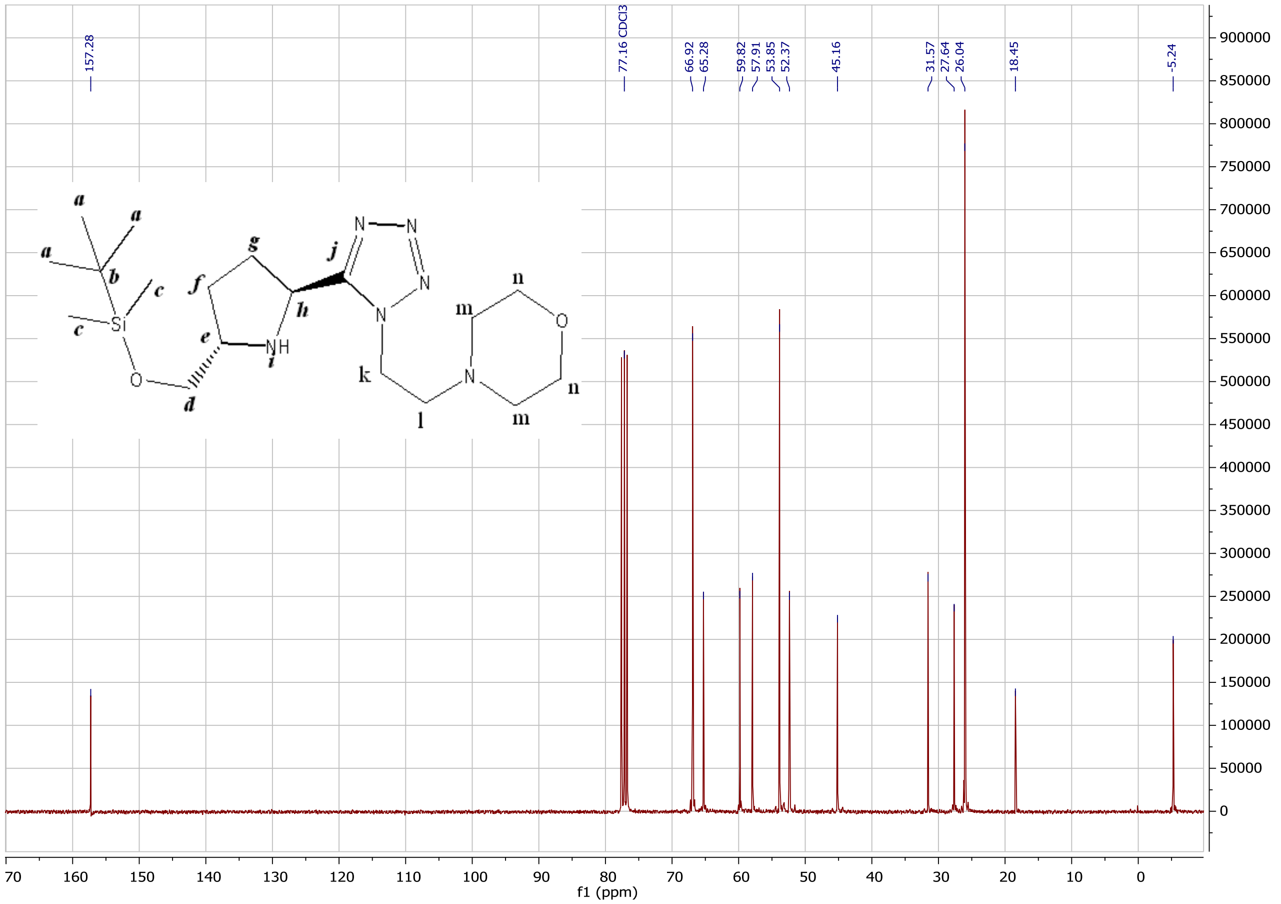

4k – *cis* isomer

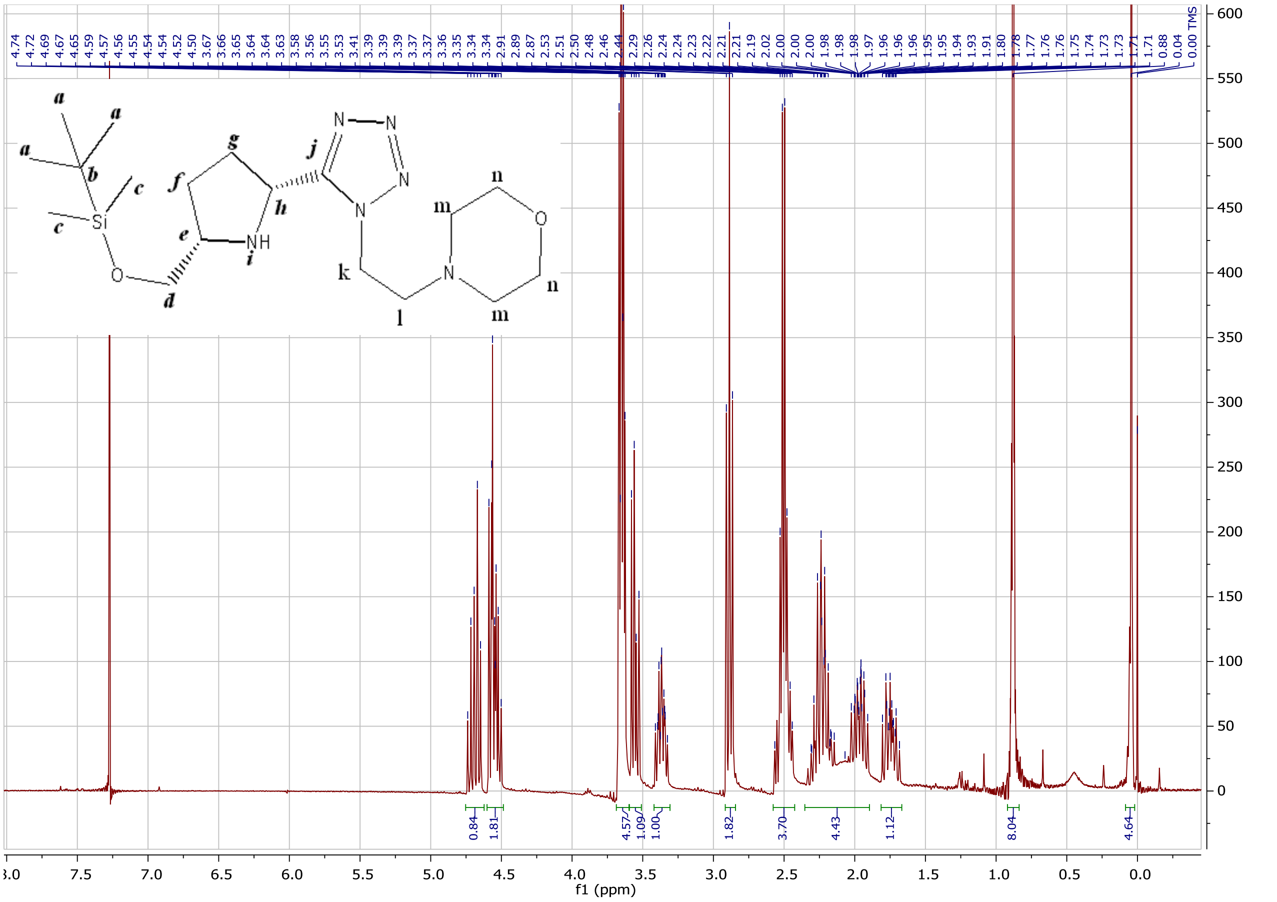

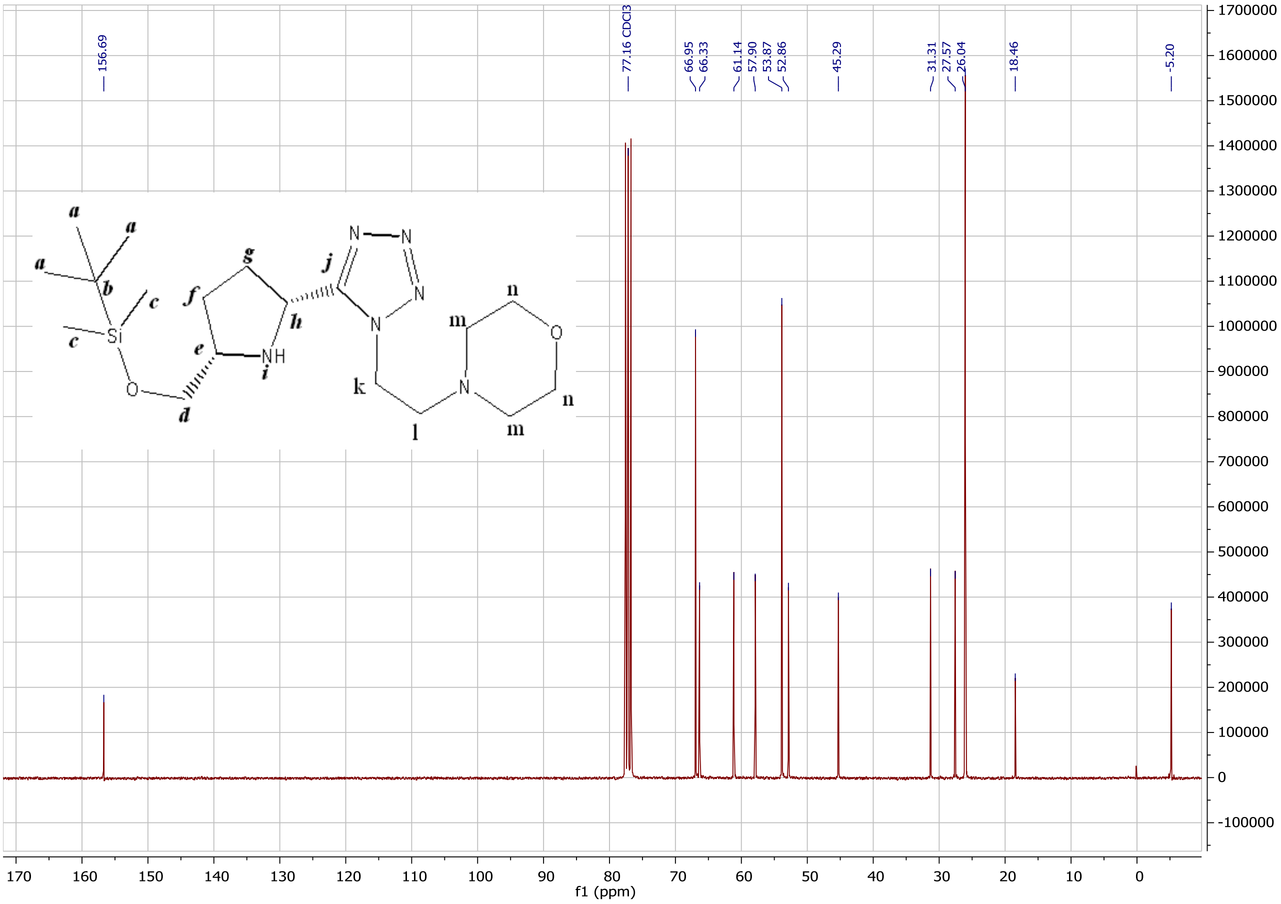

4k – NOESY 2D of *trans* and *cis* isomers

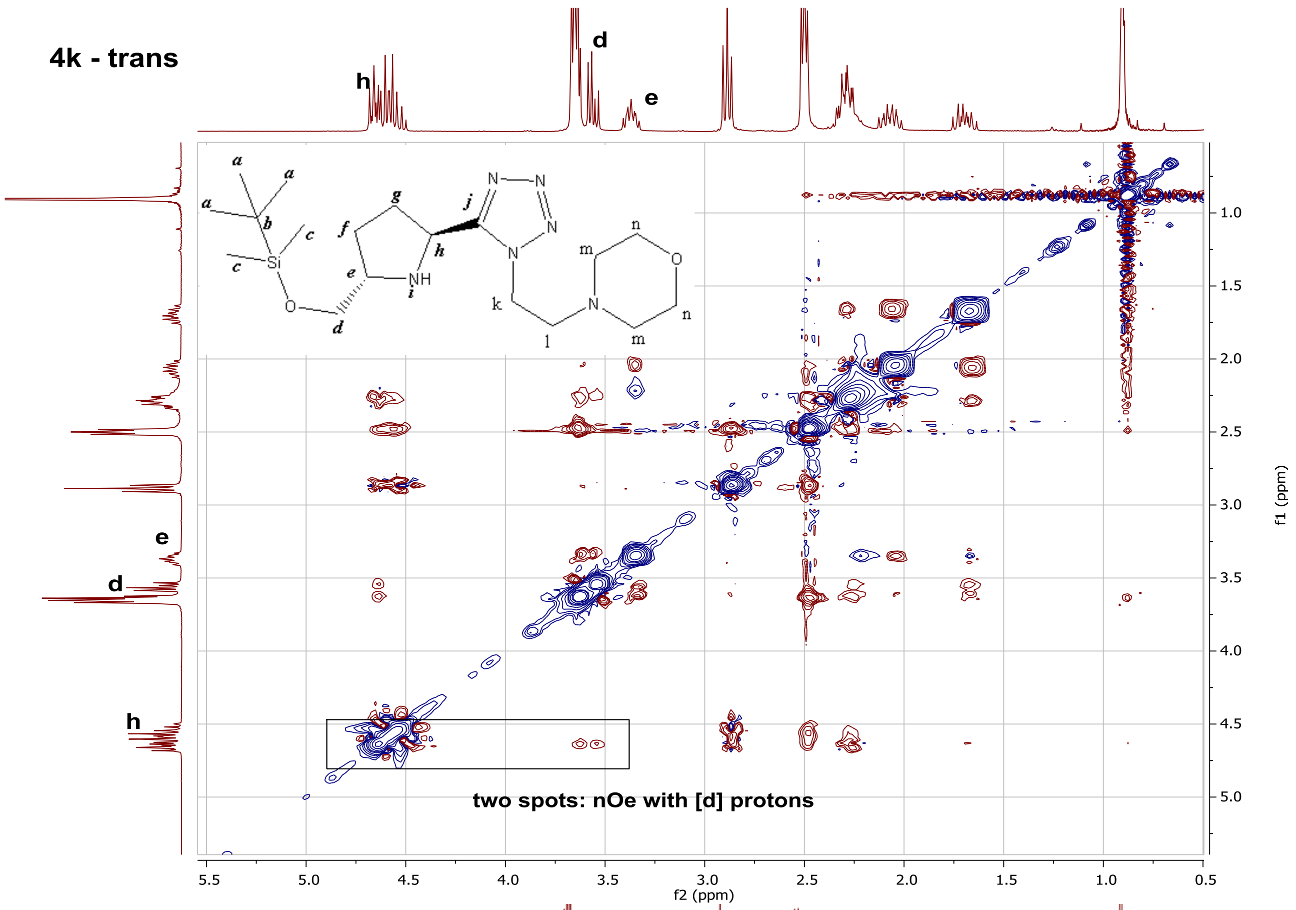

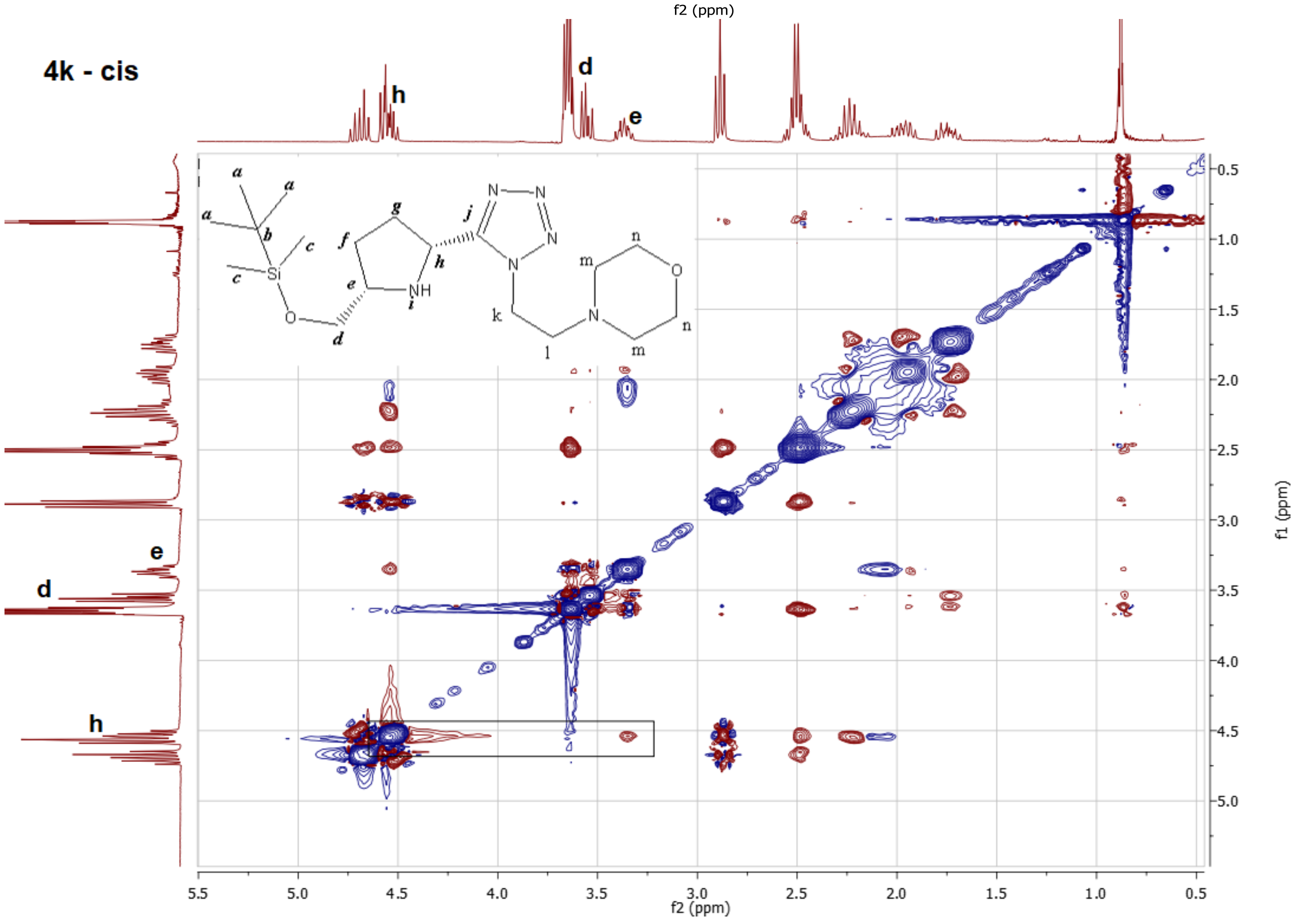

Supplement: Supplementary file 1 [file molecules-23-02758-s001.pdf]
